# The HSP40 chaperone Ydj1 drives amyloid beta 42 toxicity

**DOI:** 10.15252/emmm.202113952

**Published:** 2022-04-04

**Authors:** Julia Ring, Jelena Tadic, Selena Ristic, Michael Poglitsch, Martina Bergmann, Nemanja Radic, Dirk Mossmann, YongTian Liang, Marta Maglione, Andrea Jerkovic, Roozbeh Hajiraissi, Marcel Hanke, Victoria Küttner, Heimo Wolinski, Andreas Zimmermann, Lana Domuz Trifunović, Leonie Mikolasch, Daiana N Moretti, Filomena Broeskamp, Julia Westermayer, Claudia Abraham, Simon Schauer, Christopher Dammbrueck, Sebastian J Hofer, Mahmoud Abdellatif, Guido Grundmeier, Guido Kroemer, Ralf J Braun, Niklas Hansen, Cornelia Sommer, Mirjana Ninkovic, Sandra Seba, Patrick Rockenfeller, Friederike‐Nora Vögtle, Jörn Dengjel, Chris Meisinger, Adrian Keller, Stephan J Sigrist, Tobias Eisenberg, Frank Madeo

**Affiliations:** ^1^ Institute of Molecular Biosciences NAWI Graz University of Graz Graz Austria; ^2^ Field of Excellence BioHealth University of Graz Graz Austria; ^3^ Institute for Biochemistry and Molecular Biology ZBMZ, Medical Faculty and CIBSS ‐ Centre for Integrative Biological Signalling Studies University of Freiburg Freiburg Germany; ^4^ NeuroCure Charité Berlin Berlin Germany; ^5^ Institute for Biology Freie Universität Berlin Berlin Germany; ^6^ Technical and Macromolecular Chemistry Paderborn University Paderborn Germany; ^7^ Department of Dermatology Medical Center Freiburg Institute for Advanced Studies (FRIAS) University of Freiburg Freiburg Germany; ^8^ Institute of Biochemistry and Molecular Biology ZBMZ Faculty of Medicine University of Freiburg Freiburg Germany; ^9^ Faculty of Biology University of Freiburg Freiburg Germany; ^10^ Department of Cardiology Medical University of Graz Graz Austria; ^11^ Centre de Recherche des Cordeliers Equipe labellisée par la Ligue contre le cancer Université de Paris Sorbonne Université, Inserm U1138, Institut Universitaire de France Paris France; ^12^ Metabolomics and Cell Biology Platforms Institut Gustave Roussy Villejuif France; ^13^ Institut du Cancer Paris CARPEM Department of Biology Hôpital Européen Georges Pompidou, AP‐HP Paris France; ^14^ Research Division for Neurodegenerative Diseases Center for Biosciences Department of Medicine Faculty of Medicine and Dentistry Danube Private University Krems Austria; ^15^ Cell Biology University of Bayreuth Bayreuth Germany; ^16^ Chair of Biochemistry and Molecular Medicine Center for Biomedical Education and Research (ZBAF) University of Witten/Herdecke (UW/H) Witten Germany; ^17^ Center for Molecular Biology of Heidelberg University (ZMBH) DKFZ‐ZMBH Alliance Heidelberg Germany; ^18^ Network Aging Research Heidelberg University Heidelberg Germany; ^19^ Department of Biology University of Fribourg Fribourg Switzerland

**Keywords:** Alzheimer’s disease, amyloid beta 42, heat shock proteins, HSP40, oligomers, Molecular Biology of Disease, Neuroscience

## Abstract

Amyloid beta 42 (Abeta42) is the principal trigger of neurodegeneration during Alzheimer’s disease (AD). However, the etiology of its noxious cellular effects remains elusive. In a combinatory genetic and proteomic approach using a yeast model to study aspects of intracellular Abeta42 toxicity, we here identify the HSP40 family member Ydj1, the yeast orthologue of human DnaJA1, as a crucial factor in Abeta42‐mediated cell death. We demonstrate that Ydj1/DnaJA1 physically interacts with Abeta42 (in yeast and mouse), stabilizes Abeta42 oligomers, and mediates their translocation to mitochondria. Consequently, deletion of *YDJ1* strongly reduces co‐purification of Abeta42 with mitochondria and prevents Abeta42‐induced mitochondria‐dependent cell death. Consistently, purified DnaJ chaperone delays Abeta42 fibrillization *in vitro*, and heterologous expression of human DnaJA1 induces formation of Abeta42 oligomers and their deleterious translocation to mitochondria *in vivo*. Finally, downregulation of the Ydj1 fly homologue, Droj2, improves stress resistance, mitochondrial morphology, and memory performance in a *Drosophila melanogaster* AD model. These data reveal an unexpected and detrimental role for specific HSP40s in promoting hallmarks of Abeta42 toxicity.

## Graphical abstract

This study reports a causal link between the heat shock protein 40 (HSP40) family member Ydj1/DnaJA1 and amyloid beta 42 (Abeta42) toxicity with potential implications to Alzheimer’s disease (AD). By using AD models, Ydj1/DnaJA1 was found to drive Abeta42 pathology cascades.

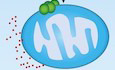

**The paper explained**

**Problem**

Alzheimer’s disease (AD) is the most prevalent age‐associated neurodegenerative disorder, worldwide. Currently, AD lacks treatment. AD is diagnosed at the time a patient already experiences progressive memory decline. Nevertheless, its molecular pathology starts several decades before symptoms onset, which is one of the challenges in research that, finally, resulted in drug development failure. The principal pathophysiological mechanism of this malady is still poorly understood. According to the amyloid hypothesis, amyloid brain burden, in particular constituted by the Amyloid beta 42 peptide (Abeta42), is central to this disease, which can hardly be studied in humans. Thus, in order to design prospering therapeutics, more extensive research using tractable model systems is needed to comprehend the mechanisms underlying AD. Even though cell and animal models per definition cannot mirror the full complexity, they offer helpful tools in dissecting conserved molecular mechanisms underlying this human disease.

**Results**

Combining yeast and fly *in vivo* AD models with an *in vitro* approach, we identified the Hsp40 chaperone Ydj1/DnaJA1 as a crucial player in Abeta42‐mediated toxicity. Expression of Abeta42 in yeast cells induced oxidative stress, reduced ATP production, and triggered mitochondria‐dependent cell death, recapitulating events occurring in AD patient brain tissue. DnaJA1 (and the respective homologues) showed physical interaction with Abeta, altered its oligomerization properties and influenced its localization within the cell. Downregulation of the DnaJA1 homologues in yeast and flies reduced Abeta42 toxicity in both model systems, which was accompanied by reduced Abeta42 translocation to mitochondria and improvement of mitochondrial morphology in yeast and flies, respectively. *Vice versa* upregulation of this Hsp40 aggravated toxicity phenotypes and favored Abeta 42 oligomerization. Using *ex vivo* analysis of brain homogenates obtained from 3xTg AD model mice and using *post mortem* brain tissue from human patients, we confirm the interaction of Abeta with mammalian DnaJA1 and its deregulation in human AD.

**Impact**

The Hsp40 chaperone DnaJA1 could be a new key player and thus a novel drug target in Abeta42 mediated toxicity. By investigating the molecular and cellular mechanisms using different model approaches, we demonstrate that DnaJA1 may favor formation or stabilization of the toxic forms of Abeta42 oligomers and influence their subcellular localization required for toxicity. Our findings challenge a dogma, which describes a purely beneficial role of HSPs counteracting neurodegeneration. Thus, our study emphasizes the importance of carefully dissecting individual Hsps functions in proteinopathies, as some chaperones might have a Janus face when it comes to Abeta toxicity.

## Introduction

Alzheimer’s disease (AD) is the most prevalent age‐associated neurodegenerative disorder and the most common cause of dementia, which affects over 55 million people worldwide according to the 2021 World Alzheimer Report (https://www.alzint.org/). The pathophysiological mechanisms underlying AD are still poorly understood and so far, this has hindered from developing efficient treatment strategies. It is generally accepted that amyloid beta (Abeta) peptides and their various aggregation forms contribute to neuronal cell death during AD. Abeta peptides arise from sequential proteolysis of a transmembrane amyloid precursor protein (APP) and vary in length, with the 42 (Abeta42)‐ and 40 (Abeta40)‐amino‐acid‐long variants being the most frequent forms (LaFerla *et al*, 2007). These peptides are highly prone to aggregate and can form low‐n oligomers (dimers, trimers, and tetramers), high‐n oligomers, protofibrils, and fibrils, as well as deposit outside neurons as large aggregates, known as extracellular senile plaques (Larson & Lesné, 2012). Abeta42 and Abeta40 aggregate via distinct pathways, impacting their degree of cytotoxicity (Bernstein *et al*, 2009). Although traditionally viewed as causing only extracellular pathology (senile plaques), the past two decades have provided increasing evidence for a critical role of intracellular Abeta42 aggregation (Haass & Selkoe, 2007; Makin, 2018; Welikovitch *et al*, 2020).

Although a large part of Abeta is released extracellularly after proteolytic processing of mature APP at the plasma membrane, it can be taken up by cells again (Umeda *et al*, 2011; Chen *et al*, 2017; Ma & Qian, 2019). Abeta has also been reported to be produced by proteolysis of APP from membranes inside the cell such as the ER or the trans‐Golgi network (Hartmann *et al*, 1997; Greenfield *et al*, 1999; Wilson *et al*, 1999). As a consequence, although still speculative, Abeta may be able to escape the secretory pathway (Bückig *et al*, 2002; Umeda *et al*, 2011), ending up in the cytosol. Intracellular Abeta has been found all over the cytoplasm, including endosomes, multivesicular bodies, lysosomes, mitochondria, ER, Golgi, and the cytosol, where it interferes with the function of diverse organelles (Skovronsky *et al*, 1998; Gouras *et al*, 2000; Goldstein *et al*, 2003; Hansson Petersen *et al*, 2008; Takahashi *et al*, 2017).

Intracellular Abeta42 oligomers, which can be detected in brain homogenates of AD patients and typically range from di‐ to dodecamers, may thus represent predominant neurotoxic assemblies of the Abeta peptides (Shankar *et al*, 2008; Sokolow *et al*, 2012; Lesné *et al*, 2013; Chen *et al*, 2017; Ono, 2018). Notably, intracellular Abeta42‐induced neurotoxicity is associated with mitochondrial dysfunction and increased production of reactive oxygen species (ROS) (Tönnies & Trushina, 2017; Terada *et al*, 2020). Yet, the molecular mechanism accounting for this toxicity is poorly understood. Abeta oligomerization and aggregation are influenced by cellular chaperones and heat shock proteins (HSPs) (Cohen *et al*, 2006). Several studies have indicated protective effects of HSP70 or HSP90 family members in AD, since they interfere with Abeta aggregation (Lazarev *et al*, 2017). However, Abeta42 toxicity does not correlate with increasing size of aggregates, but rather with the presence of distinct Abeta oligomers leading to impaired synapse structure and function (Shankar *et al*, 2008). Therefore, HSPs that inhibit the formation of fibrils and/or large aggregates, but do not prevent accumulation of oligomers, may additionally have counterintuitive, maladaptive effects.

The yeast *Saccharomyces cerevisiae* is a eukaryotic model organism that is suitable for studying aspects of Abeta toxicity (i.e., impaired cell growth, cell death, and oxidative stress being most frequently addressed hallmarks of Abeta toxicity in yeast) since it combines a conserved protein quality control network with the possibility of large‐scale genetic investigations (Braun *et al*, 2010). Yeast AD models have been established by several groups (Caine *et al*, 2007; Park *et al*, 2011a; Treusch *et al*, 2011; Fruhmann *et al*, 2018; Chen *et al*, 2020, 2021). Since Abeta42 itself is unstable when expressed in yeast, fusion tags, such as green fluorescent protein (GFP), maltose binding protein, or the essential functional domain of translational release factor Sup35, are utilized for a stable intracellular expression of Abeta42 (Caine *et al*, 2007; Park *et al*, 2011a; Fruhmann *et al*, 2018). Alternatively, fusion of Abeta42 to the Kar2 secretory sequence, which directs it to the secretory pathway, has been successfully used to cause toxicity in yeast (Treusch *et al*, 2011).

In this study, we aimed to investigate mechanisms of toxicity induced by intracellular Abeta42 using Abeta42‐expressing yeast and fly models, combined with *in vitro* and *ex vivo* approaches. Here, we identify the HSP40 family member Ydj1 (DnaJA1 in humans) as a key player in intracellular Abeta42‐triggered toxicity. Mechanistically, we propose that Ydj1/DnaJA1 drives mitochondria‐dependent cell death through stabilization of Abeta42 oligomers and their translocation to mitochondria.

## Results

### Human Abeta42 triggers oxidative stress and necrotic‐like cell death in yeast

**Figure 1:**
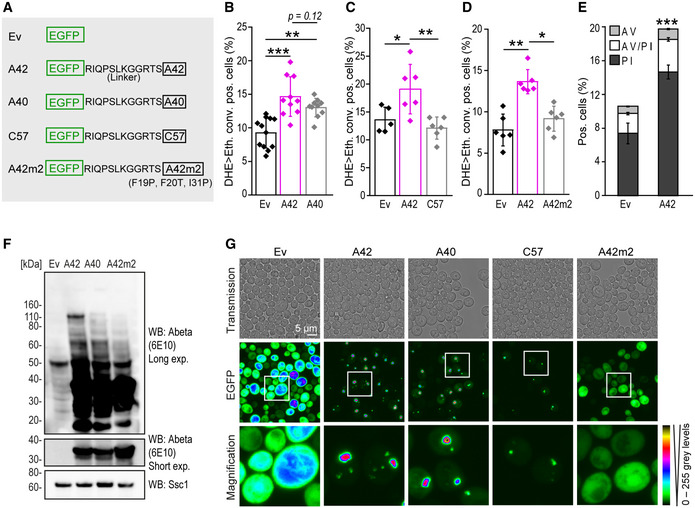
Human A42 forms oligomers and triggers oxidative stress and cell death in yeast ASchematic illustration of EGFP‐linker‐A42 fusion protein (A42) and corresponding controls: EGFP empty vector (ev), EGFP‐A40 (A40), EGFP‐C57 (C57), and EGFP‐A42m2 (A42m2) containing three‐point mutations as indicated.B–DFlow cytometric quantification of DHE>Eth. positive wild‐type yeast cells (allowing the detection of both dead cells and cells exhibiting sub‐lethal oxidative stress) after 42 h (B) or 66 h (C, D) expressing Abeta constructs. Dot plots show all data points along with the mean (bar) ± SD *n* = 5–12 biologically independent cultures. ***P < 0.001, **P < 0.01, *P < 0.05. ANOVA with Tukey’s *post hoc* test.EAnnexin V (AV)/PI co‐staining to assess cell death in yeast‐expressing A42 after 48 h. Data represent means ± SD *n* = 6 biologically independent cultures. ***P < 0.001 (comparing PI positive populations). Unpaired, two‐tailed *t*‐test.FImmunoblot of whole‐cell extract (WCE) of wild‐type yeast cells after 16 h expression of Abeta constructs. For western blot (WB) Abeta‐specific antibody (Abeta) 6E10 was used showing long (Long exp.) and short time exposure (Short exp.). Ssc1 was used as a loading control. See also Appendix Fig S1D.GConfocal microscopy of wild‐type yeast cells after 18 h of Abeta expression. Colors indicate fluorescence intensity. Source data are available online for this figure.

In an attempt to establish a yeast model to study the cytotoxic effects of intracellular Abeta42, we created an EGFP‐Abeta42 fusion protein (EGFP‐A42) that was heterologously expressed in *S. cerevisiae*. We used an N‐terminal EGFP fusion tag, separated by a linker to ensure proper folding and function of both EGFP and Abeta42. Of note, this configuration allows oligomerization of Abeta42 due to an accessible C‐terminus (Bernstein *et al*, 2009). As controls, we used strains carrying the empty vector expressing EGFP only (ev), as well as two alternative APP‐derived peptides fused to EGFP (Fig 1A), including C57 (EGFP‐C57), the non‐toxic peptide simultaneously generated along with Abeta42 in the last step of amyloidogenesis, and the shorter and often referred to as less toxic Abeta40 peptide (EGFP‐A40) (LaFerla *et al*, 2007). Additionally, we expressed a mutated form of Abeta42 (EGFP‐A42m2), which forms fewer oligomeric assemblies due to three‐point mutations present in two of four aggregation‐prone regions present in the Abeta peptide (F19P, F20T, I31P) (Park *et al*, 2011a). Successful expression of all fusion constructs was assessed by immunoblotting and fluorescence microscopy (Fig 1F and G, Appendix Fig S1D).

To investigate potential toxic effects of Abeta42, we analyzed EGFP‐Abeta expression in wild‐type cells grown to stationary phase, an established model for studying hallmarks of aging post‐mitotic higher eukaryotic cells. These hallmarks include increased production of ROS and cell death (Fabrizio *et al*, 2004; Herker *et al*, 2004; Longo *et al*, 2012). Expression of EGFP‐A42 did not impair growth (Appendix Fig S1A), but led to an increase in the fraction of dead cells or cells exhibiting oxidative stress, as assessed by the number of dihydroethidium‐to‐ethidium (DHE>Eth.) positive cells compared to all corresponding non‐toxic controls (ev, EGFP‐C57 and EGFP‐A42m2) (Fig 1B–D). Phenotypic inspection of cell death by annexin V/propidium iodide (PI) co‐staining revealed a primarily necrotic‐like phenotype (PI single‐positive cells) upon Abeta42 expression (Fig 1E) (Madeo *et al*, 1997, 1999; Eisenberg *et al*, 2010).

**Figure EV1:**
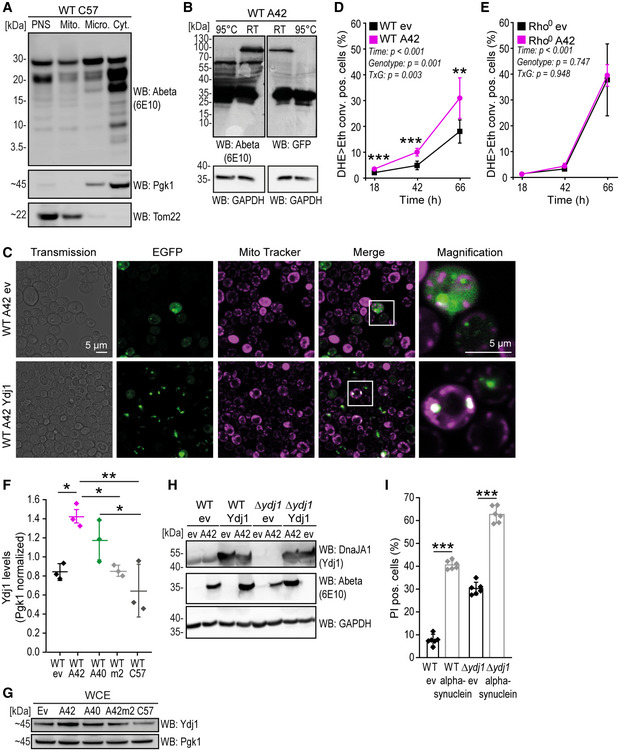
Human A42 shows mitochondria‐associated toxicity dependent on Ydj1 levels AImmunoblot of total cytoplasmic post‐nuclear supernatant (PNS), mitochondrial, microsomal, and cytosolic fractions of wild‐type yeast cells after 18 h expression of EGFP‐C57 (C57) using EGFP‐specific antibody (GFP). Purity of fractions was tested with antibodies against Tom22 (mitochondria) and Pgk1 (cytosol).BImmunoblot of whole‐cell extract (WCE) of wild‐type (WT) yeast cells after 16 h of expression of EGFP‐A42. Samples were either kept at RT or heated to 95°C before loading on the SDS gel. Abeta‐specific antibody (Abeta) 6E10 and EGFP antibody (GFP) were used for immunoblotting. GAPDH was used as a loading control.CFluorescence microscopy of wild‐type (WT) yeast cells after 18 h of expression of EGFP‐A42 (A42) and co‐overexpressing Ydj1‐FLAG (Ydj1) or EGFP empty vector (ev). Mitochondria were visualized with MitoTracker Red (magenta).D, EQuantification of DHE>Eth. positive cells at indicated time points during chronological aging of wild‐type (WT) cells and cells lacking mitochondrial DNA (Rho0), expressing EGFP‐A42 (A42) or EGFP only (ev). Mean ± SD *n* = 4–6 biologically independent cultures. *P*‐values by two‐way repeated measures ANOVA followed by simple main effects (***P < 0.001; **P < 0.01, versus control).FQuantification of intensities of Ydj1‐specific antibody (Ydj1) bands of the immunoblot representatively shown in G and Appendix Fig S1B, normalized to intensities of Pgk1‐specific antibody bands (Pgk1). Dot plots show all data points along with the mean (line) ± SD *n* = 3 biologically independent cultures. **P < 0.01; *P < 0.05. ANOVA with Tukey’s *post hoc* test.GImmunoblot of whole‐cell extract (WCE) of wild‐type yeast cells after 16 h of expression of EGFP‐A42. Pgk1 was used as a loading control. See also Appendix Fig S1B.HImmunoblot of whole‐cell extract (WCE) of wild‐type and Δ*ydj1* yeast cells after 16 h of expression of EGFP‐A42 and co‐overexpressing Ydj1‐FLAG (Ydj1) or harboring the corresponding vector controls. DnaJA1‐ and Abeta‐specific (6E10) antibodies were used for immunoblots. GAPDH was used as a loading control.IQuantification of PI positive wild‐type (WT) and Δ*ydj1* cells after 42 h of expressing alpha‐synuclein or harboring the empty vector control (ev). Dot plots show all data points along with the mean (bar) ± SD *n* = 6 biologically independent cultures. ***P < 0.001. ANOVA with Tukey’s *post hoc* test. Source data are available online for this figure.

Confocal microscopy of EGFP‐A42 showed that intracellular Abeta42 accumulated and formed large aggregates as well as smaller punctuate structures (Fig 1G). EGFP‐A40 and EGFP‐C57 also formed punctuate structures within the cell, with EGFP‐C57 aggregates being less prominent than EGFP‐A40 or EGFP‐A42. In line with its reported inability to form oligomers and aggregates (Bagriantsev & Liebman, 2006; Park *et al*, 2011b), EGFP‐A42m2 showed a diffuse cytosolic fluorescence, similar to the EGFP‐expressing control (Fig 1G). Immunoblot analyses under oligomer preserving conditions confirmed the presence of specific Abeta42 oligomers (presumably dimers and tetramers), as indicated by the detection of several electrophoretically less mobile bands in addition to the monomeric full‐length EGFP‐A42 fusion protein (~35 kDa) using both 6E10 (Fig 1F) and EGFP (Appendix Fig S1D) antibodies. Interestingly, such small Abeta42 oligomers (low‐n oligomers) are believed to be the most toxic forms of Abeta42 aggregation products in AD (Chen *et al*, 2017; Ono, 2018). Consistent with this notion, the oligomeric forms of Abeta were primarily detected in cells expressing EGFP‐A42, but to a lesser extent or not in cells expressing EGFP‐A40 or EGFP‐A42m2 control peptides, respectively (Fig 1F). The tetrameric form of EGFP‐A42, detected at ~120 kDa, decomposed when samples were heated at 95°C before loading on SDS‐gel (Fig EV1B), indicating a *bona fide* oligomeric assembly, since heat instability is a reported characteristic of Abeta oligomers (Park *et al*, 2011a). Thus, and because this tetrameric EGFP‐A42 assembly was most robustly detected by immunoblot in all of our experimental conditions, we used this as a representative oligomer for further quantitative analysis.

### Human Abeta42 translocates to mitochondria in yeast cells

**Figure 2:**
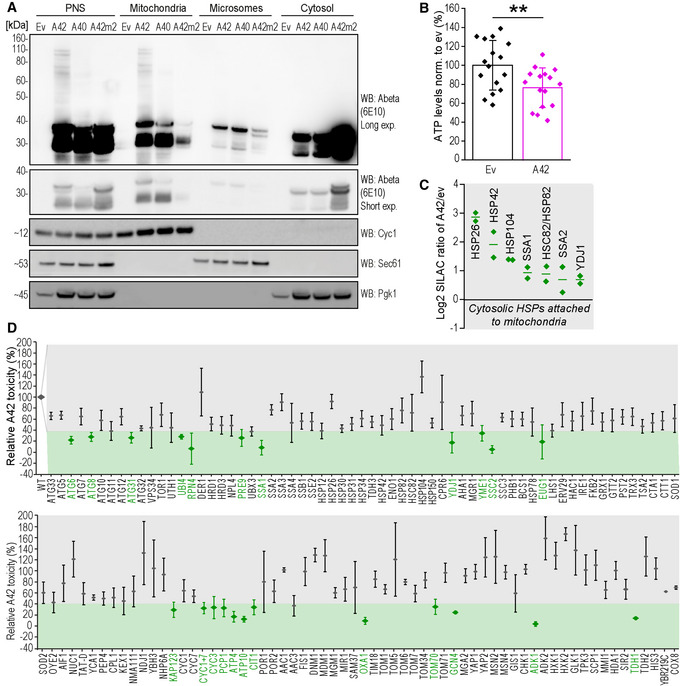
Human A42 oligomers show mitochondria proximal localization, reduce ATP, and increase the presence of cytosolic HSPs at mitochondria Immunoblot of total cytoplasmic post‐nuclear supernatant (PNS), mitochondrial, microsomal, and cytosolic fractions of wild‐type yeast cells after 18 h expression of EGFP‐Abeta42 (A42), EGFP‐Abeta40 (A40), and EGFP empty vector (ev) using Abeta‐specific antibody (Abeta) 6E10 with long (Long exp.) and short time exposure (Short exp.). Purity of fractions was tested with antibodies against Cyc1 (mitochondria), Sec61 (microsomes), and Pgk1 (cytosol).Cellular ATP content of wild‐type yeast cells after 42 h expression of EGFP‐A42 (A42) or EGFP empty vector (ev). Dot plots show all data points along with the mean (bar) ± SD *n* = 16 biologically independent cultures. **P < 0.01. Unpaired, two‐tailed *t*‐test.Relative protein abundance of EGFP‐Abeta42 (A42) versus empty vector (ev)‐expressing wild‐type yeast cells. The significant subset of cytosolic heat shock proteins (HSP) detected in a proteomics analysis of isolated mitochondria is depicted. Log2 SILAC ratios of A42/ev of two independent proteome measurements are shown. Dot plots show all data points along with the mean (line). Significance was determined using an outlier test (Significance A, *P* < 0.003). See also Appendix Table S3.Screen of 123 deletion strains assessing Abeta42 (A42)‐induced toxicity (assessed by DHE>Eth. positive cells indicative of the sum of sub‐lethal oxidative stress and cell death) in yeast. Relative A42 toxicity normalized to wild‐type (WT) cells is depicted. Potential hits (mutants that reduce A42 toxicity) are shown in green. Data represent means ± SD *n* ≥ 3 biologically independent cultures. See methods section for details on screening and hit criteria. Source data are available online for this figure.

Abeta translocation to or into mitochondria has been found in AD patients, as well as in other AD models (Hansson Petersen *et al*, 2008; Walls *et al*, 2012), including isolated yeast mitochondria (Mossmann *et al*, 2014). Similar to these findings, confocal microscopy analysis showed that EGFP‐A42 localized close to MitoTracker‐stained mitochondria (Fig EV1C). To further investigate the subcellular localization of EGFP‐A42 and Abeta42 oligomers, we performed cell fractionation by differential centrifugation followed by immunoblot analyses (Fig 2A). EGFP‐A42 was predominantly detected in the mitochondria‐enriched fraction, and to a lesser extent in cytosol‐ and microsome‐enriched fractions. Of note, oligomers were enriched in the mitochondrial fraction and not detectable in the fractions enriched of cytosol and microsomes (Fig 2A). Similarly, EGFP‐A40 was also detected primarily in the mitochondrial fraction, whereas the mutated Abeta42m2 as well as C57 exclusively localized to the cytosol (Figs 2A and EV1A).

In summary, we established a yeast system for intracellular (cytosolically expressed) Abeta42 formation that recapitulates several cellular hallmarks associated with AD, including oxidative stress, cell death, Abeta42 oligomerization and aggregation, as well as Abeta42 mitochondria proximal localization.

### Functional mitochondria are crucial for Abeta42‐mediated cell death

Cells expressing EGFP‐A42 showed decreased ATP levels compared to the EGFP‐expressing vector control (Fig 2B), suggesting that Abeta42 perturbs mitochondrial function. Accordingly, a respiration‐deficient strain lacking mitochondrial DNA (Rho^0^) prevented EGFP‐A42‐mediated cell death (Fig EV1D and E). Intrigued by the mitochondria proximal localization of EGFP‐A42, the importance of mitochondrial DNA for Abeta42‐induced cell death, and the impairment of mitochondrial function upon Abeta42 expression, we investigated possible changes in the mitochondrial proteome using stable isotope labeling‐based quantitative proteomics of mitochondria isolated from EGFP‐A42‐expressing or EGFP‐expressing control cells. Of note, beside mitochondrial proteins, also cytosolic proteins co‐purified with mitochondria. Interestingly, several cytosolic HSPs were enriched in the purified mitochondrial fraction upon EGFP‐A42 expression (Fig 2C, Appendix Table S3).

Additionally, we performed a genetic screen, expressing EGFP‐A42 in a set of 123 haploid single‐gene deletion strains of genes related to neurodegeneration processes, including known regulators and executors of cell death, protein degradation and stability, stress response, and mitochondrial function (Fig 2D). As sensitive readout for toxicity, the fraction of DHE>Eth. positive cells was quantified (42–48 h) after EGFP‐A42 expression because it allows the detection of both dead cells and cells exhibiting oxidative stress. At this time point all strains have grown to stationary phase and show a proper expression of EGFP‐A42 with on average a two‐fold increase in DHE>Eth. positive cells in the WT condition. The effect of EGFP‐A42 expression in each deletion strain was compared to wild‐type cells as well as three randomly picked deletion strains (*HIS3*, *COX8,* and *YBR219C*, a protein of unknown function), which served as negative controls. Wild‐type yeast cells showed reproducible increase in DHE>Eth. positive cells in 42–48 h cultures, which was defined as 100% toxicity (Fig 2D). Strains showing less than 40% toxicity were regarded as potential hits. Noteworthy, executors of apoptosis, such as the yeast caspase Yca1, yeast homologue mammalian HtrA2 family proteins Nma111, as well as three mitochondria‐mediated key players of apoptosis, including Aif1, Nuc1, and Ybh3 (Wissing *et al*, 2004; Büttner *et al*, 2006, 2011, 2013), failed to affect intracellular EGFP‐A42‐mediated oxidative stress. This outcome goes in line with the finding that cells expressing EGFP‐A42 exhibited a necrotic rather than apoptotic cell death morphology (Fig 1E). Instead, our screen revealed a prominent involvement of mitochondria‐ and mitochondrial function‐associated proteins in Abeta42‐induced oxidative stress. Twelve of 23 hits were either mitochondrial proteins or proteins involved in oxidative phosphorylation. In addition to three mitochondria‐unrelated gene deletions that, however, do cause respiratory deficiency (*EUG1*, *GCN4*, and *TDH1*), we identified several genes involved in protein quality control, including three autophagy‐related genes (*ATG6*, *ATG8,* and *ATG31*), three genes relevant for the proteasome machinery (*UBI4*, *RPN4*, and *PRE9*), and two heat shock proteins (*SSA1* and *YDJ1*) to be required for full EGFP‐A42 toxicity.

Intriguingly, the cytosolic and mitochondria‐associated HSP40 family member, Ydj1, which was increased two‐fold at mitochondria in cells expressing EGFP‐A42 within the proteomic analysis (Fig 2C), also appeared as a hit in the genetic screen (Fig 2D). Immunoblotting of whole‐cell extracts using a Ydj1‐specific antibody confirmed an increase in Ydj1 in the presence of EGFP‐A42 (Fig EV1F and G, Appendix Fig S1B).

Overall, both of our approaches demonstrated the involvement of mitochondria in the cellular response to EGFP‐A42 expression and both revealed Ydj1 as a potential amplifier of Abeta42‐mediated toxicity.

### Ydj1 is crucial for Abeta42‐induced cell death by stabilizing Abeta42 oligomers and mediating their translocation to mitochondria

**Figure 3:**
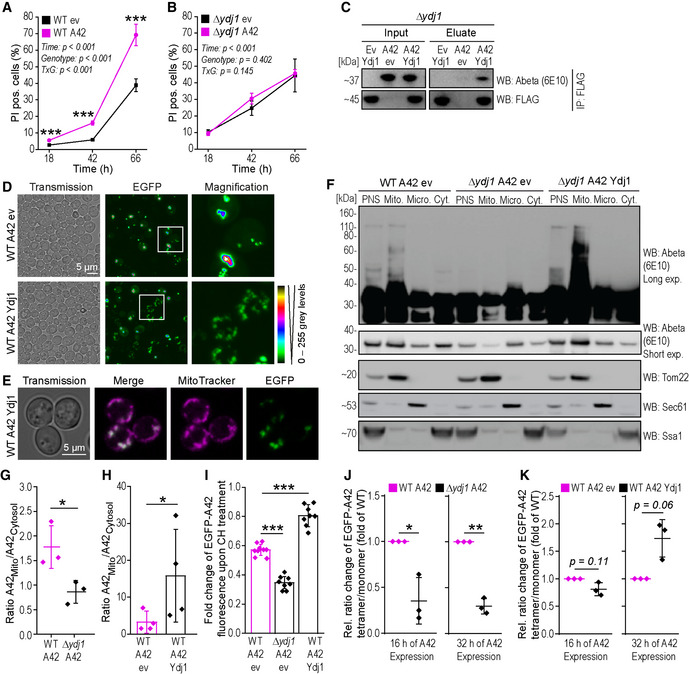
Ydj1 is required for Abeta42‐induced cell death and drives Abeta42 oligomer formation and translocation to mitochondria A, BQuantification of cells positive for propidium iodide (PI)‐staining indicating cell death at indicated time points after start of expression of EGFP‐A42 (A42) or EGFP empty vector (ev) of wild‐type (WT) (A) and *YDJ1*‐deleted (Δ*ydj1*) cells (B). Mean ± SD *n* = 5 biologically independent cultures. Comparisons by two‐way ANOVA (mixed‐design) followed by simple main effects (***P < 0.001, versus control).CImmunoblot (WB) of whole‐cell extract (Input) and eluate of FLAG‐tagged Ydj1. Immunoprecipitation (IP: FLAG) of *YDJ1* deletion strain (Δ*ydj1*) cells expressing EGFP‐A42 (A42) or EGFP empty vector (ev) and co‐overexpressing Ydj1‐FLAG (Ydj1) using Abeta‐specific antibody (6E10) and FLAG antibody (FLAG).DConfocal microscopy of wild‐type (WT) yeast cells expressing EGFP‐A42 (A42) only and co‐overexpressing Ydj1‐FLAG (Ydj1) or with the corresponding vector control after 18 h of expression. Colors indicate fluorescence intensity.EConfocal microscopy of wild‐type (WT) yeast cells expressing EGFP‐A42 (A42) and co‐overexpressing Ydj1‐FLAG (Ydj1) after 18 h of expression. Mitochondria were visualized with MitoTracker Red (magenta). See also Figs EV2J and EV1C.FImmunoblot (WB) of total cytoplasmic post‐nuclear supernatant (PNS), mitochondrial (Mito.), microsomal (Micro.), and cytosolic (Cyt.) fractions of wild‐type (WT) and *YDJ1* deletion strain (Δ*ydj1*) cells after 18 h of expression of EGFP‐A42 (A42) and co‐overexpressing Ydj1‐FLAG (Ydj1) or corresponding empty vector controls (ev) using Abeta‐specific antibody (Abeta) 6E10 with long (Long exp.) and short time exposure (Short exp.). Tom22‐specific antibody is a marker for mitochondria, Sec61 for microsomes, and Ssa1 was used to verify cytosolic fraction.G, HRatio of full‐length EGFP‐A42 in the mitochondrial fraction / cytosolic fraction, using densitometry quantification of the immunoblot representatively shown in Fig 3F and Appendix Fig S1C from three or four independent experiments. Dot plots show all data points along with the mean (line/bar) ± SD *n* = 3–4 biologically independent cultures. *P < 0.05. Unpaired, two‐tailed *t*‐test.IAssay for protein degradation using cycloheximide (CH) to stall protein translation. EGFP fluorescence intensity was measured at two time points (t0 and t2, 2 h after CH administration) and normalized to t0 in wild‐type (WT) and *YDJ1* deletion strain (Δ*ydj1*) cells after 18 h of expression of EGFP‐A42 (A42) and co‐overexpressing Ydj1‐FLAG (Ydj1) or corresponding empty vector controls (ev). Dot plots show all data points along with the mean (bar) ± SD *n* = 8 biologically independent cultures. ***P < 0.001. ANOVA with Tukey’s *post hoc*. See also Appendix Fig S2C.J, KQuantification of the ratio between EGFP‐A42 tetramer and monomer in wild‐type (WT) and *YDJ1* deletion strain (Δ*ydj1*) expressing EGFP‐A42 (J) as well as between WT expressing EGFP‐A42 only and co‐overexpressing Ydj1‐FLAG (Ydj1) (K) at indicated time points. Dot plots show all data points along with the mean (line) ± SD *n* = 3(J) *n* = 3(K) biologically independent cultures. **P < 0.01; *P < 0.05. One sample *t*‐test against 1. See also Appendix Fig S2A and B. Source data are available online for this figure.

**Figure EV2:**
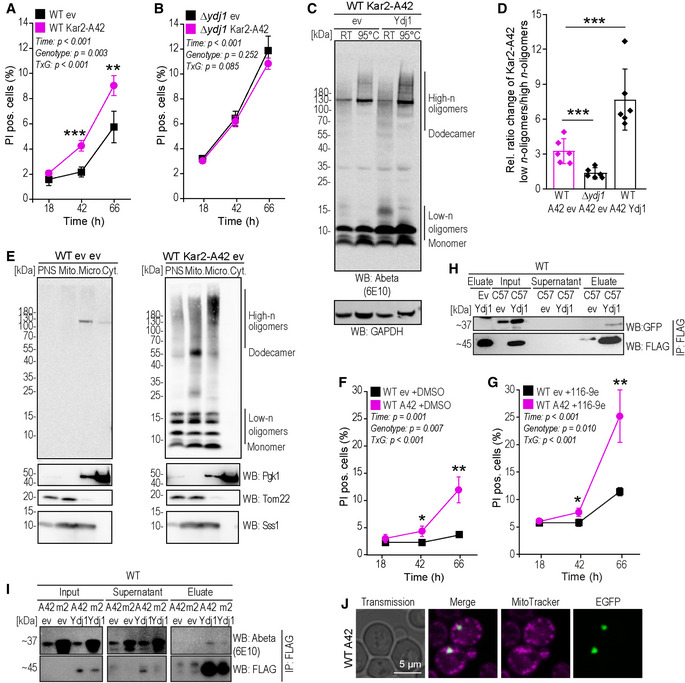
Validation of Ydj1 effects toward Abeta‐induced phenotypes in alternative AD yeast model expressing Kar2‐A42 guiding Abeta42 to the secretory pathway A, BQuantification of PI positive cells at indicated time points during chronological aging of wild‐type (WT) (A) and Δ*ydj1* (B) cells expressing Kar2‐A42 or harboring the empty vector control (ev). Mean ± SD *n* = 6 biologically independent cultures. *P*‐values by two‐way repeated measures ANOVA followed by simple main effects (***P < 0.001; **P < 0.01, versus control).CImmunoblot of whole‐cell extract (WCE) of wild type (WT) after 16 h of expression of Kar2‐A42 and expressing Ydj1‐FLAG (Ydj1) or harboring the corresponding vector control (ev). Samples were either kept at RT or heated to 95°C before loading on the 4–12% NuPage Bis–Tris gel. Abeta‐specific antibody (Abeta) 6E10 was used for immunoblotting. GAPDH was used as a loading control.DQuantification of the ratio between A42 low‐n oligomers and high‐n oligomers in wild‐type (WT) and *YDJ1* deletion strain (Δ*ydj1*) expressing Kar2‐A42 as well as between wild‐type (WT) expressing Kar2‐A42 only or co‐overexpressing Ydj1‐FLAG (Ydj1) after 16 h of expression from immunoblots representatively shown in (C). Dot plots show all data points along with the mean (bar) ± SD *n* = 6 biologically independent cultures. ***P < 0.001. ANOVA with Tukey’s *post hoc* test.EImmunoblot of total cytoplasmic post‐nuclear supernatant (PNS), mitochondrial, microsomal, and cytosolic fractions of wild‐type yeast cells after 18 h expression of Kar2‐Abeta42 (A42) and empty vector (ev) using Abeta‐specific antibody (WB: Abeta) 6E10. Purity of fractions was tested with antibodies against Tom22 (mitochondria), Sss1 (microsomes), and Pgk1 (cytosol).F, GQuantification of PI positive cells at indicated time points during chronological aging of wild‐type (WT) cells expressing EGFP‐A42 or harboring the empty vector control (ev) upon treatment with Hsp70/Hsp40 interaction inhibitor 116‐9e (G) or corresponding DMSO control (F). Mean ± SD *n* = 6 biologically independent cultures. *P*‐values by two‐way repeated measures ANOVA followed by simple main effects (**P < 0.01; *P < 0.05, versus control).HImmunoblot of whole‐cell extract (WCE), supernatant, and eluate of FLAG‐tagged Ydj1. Immunoprecipitation (IP: FLAG) of wild‐type (WT) cells expressing EGFP‐C57 (C57) or EGFP only (ev) and co‐overexpressing Ydj1‐FLAG (Ydj1) using EGFP antibody (GFP) and FLAG antibody (FLAG).IImmunoblot of whole‐cell extract (WCE), eluate, and supernatant of FLAG‐tagged Ydj1. Immunoprecipitation (IP: FLAG) of wild‐type (WT) cells expressing EGFP‐A42 (A42), A42m2, or EGFP only (ev) and co‐overexpressing Ydj1‐FLAG (Ydj1) using Abeta‐specific antibody (Abeta) 6E10 and FLAG antibody (FLAG).JConfocal microscopy of wild‐type (WT) yeast cells expression of EGFP‐A42 (A42) after 18 h of expression. Mitochondria were visualized with MitoTracker Red (magenta). Source data are available online for this figure.

Since Ydj1 emerged as a top hit in both the genetic screen and the proteomic approach, we further investigated how altering Ydj1 levels by knockout (Fig EV1H) affected EGFP‐A42 toxicity. EGFP‐A42‐triggered oxidative stress and propidium iodide–detectable cell death were reduced to control levels in the Δ*ydj1* strain (Figs 2D and 3A and B). Ydj1 has been reported to act in part through interaction with Hsp70, activating Hsp70’s ATPase activity. Therefore, in an attempt to mimic the effects of *YDJ1* deletion, we used the Hsp40–Hsp70 interaction inhibitor 116‐9e (Wisén *et al*, 2010). Treatment with 116‐9e did not lower but rather increased cell death in wild‐type cells expressing EGFP‐42 compared to vehicle (DMSO)‐treated controls (Fig EV2F and G), arguing for an Hsp70‐independent function of Ydj1 to be crucial for Abeta42 toxicity. Interestingly, lowering toxicity by *YDJ1* deletion appeared to be specific for Abeta42, as deletion of *YDJ1* did not reduce alpha‐synuclein‐induced cell death (Fig EV1I) in a yeast model for Parkinson’s disease (Büttner *et al*, 2008). Importantly, deletion of *YDJ1* also abolished Abeta42‐induced cell death in an alternative yeast model where Abeta42 was fused to the Kar2 localization sequence, which directs the Abeta42 peptide to the secretory pathway (Treusch *et al*, 2011) (Fig EV2A and B).

To examine a possible physical interaction of Ydj1 and EGFP‐A42 as well as corresponding control peptides ‐C57 and ‐A42m2, we performed an *in vitro* pull‐down experiment using strains co‐expressing EGFP‐A42, ‐C57, or ‐A42m2 together with FLAG‐tagged Ydj1 (Ydj1‐FLAG). Immunoprecipitation of Ydj1‐FLAG using an anti‐FLAG antibody was performed followed by western blot analysis of the eluate using Abeta‐ or EGFP‐specific antibodies to test for co‐immunoprecipitation of A42 and A42m2 or C57, respectively. Both EGFP‐A42 and EGFP‐C57 (Figs 3C and EV2H) were co‐immunoprecipitated with Ydj1‐FLAG, indicating an interaction with Ydj1. An interaction with EGFP‐A42m2, however, was not observed (Fig EV2I), in accord with the notion that Ydj1 interacts with hydrophobic peptides and proteins (Li *et al*, 2003).

HSP40 family members reportedly prevent the formation and/or assist decomposition of aggregates and guide shuttling proteins to their subcellular destinations (Walsh *et al*, 2004). Thus, we next explored if Ydj1 could modulate EGFP‐A42 assembly composition and mediate EGFP‐A42 translocation to mitochondria. Indeed, confocal microscopy revealed that overexpression of Ydj1 favored smaller, dispersed assemblies (Fig 3D), which were not visible in WT EGFP‐A42 cells. Of note, these smaller assemblies were prominently observed at mitochondria labeled with the mitochondria‐specific fluorescence dye MitoTracker Red (Figs 3E and EV2J). In line, overexpression of Ydj1 also increased the presence of EGFP‐A42 in the mitochondrial fraction upon cell fractionation (Fig 3F, Appendix Fig S1C). Conversely, cell fractionation of the Δ*ydj1* strain showed that the mitochondrial localization of EGFP‐A42 observed in WT cells was mainly lost in the absence of Ydj1 (Fig 3F). Importantly, the ratio of mitochondrial versus cytosolically localized Abeta was significantly reduced upon deletion of *YDJ1* and enhanced upon Ydj1 overexpression (Fig 3G and H). This strongly indicates that Ydj1 is responsible for the transport of EGFP‐A42 and its oligomers to mitochondria, which is in line with Ydj1’s function in mitochondrial protein translocation (Caplan *et al*, 1992; Jores *et al*, 2018).

**Figure EV3:**
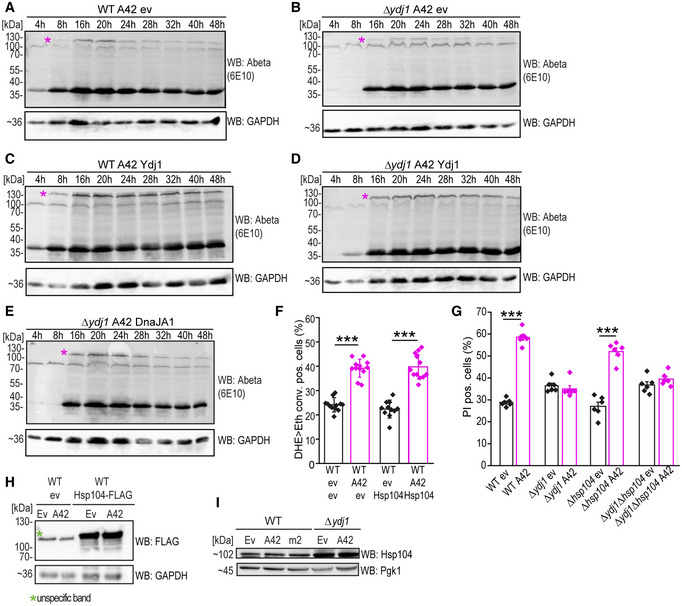
Ydj1/DnaJA1 effects toward Abeta oligomerization and cytotoxicity are independent of Hsp104 levels A–DImmunoblot of time series of whole‐cell extract (WCE) of wild‐type (WT) and *YDJ1* deletion (Δ*ydj1*) cells expressing Ydj1 or harboring the corresponding empty vector control (ev) after indicated hours (h) of expression of EGFP‐A42 using Abeta‐specific antibody (Abeta) 6E10. Star* indicates the tetramer.EImmunoblot of time series of Abeta42 expression in *YDJ1* deletion (Δ*ydj1*) cells co‐expressing DnaJA1 after indicated hours (h) of expression of EGFP‐A42 using Abeta‐specific antibody (6E10). Star* indicates the tetramer.FQuantification of DHE>Eth. positive wild type (WT) after 66 h of expressing EGFP‐A42 (A42) and Hsp104 or harboring the corresponding empty vector controls (ev). Dot plots show all data points along with the mean (bar) ± SD *n* = 12 biologically independent cultures. ***P < 0.001. ANOVA with Tukey’s *post hoc* test.GQuantification of PI positive wild‐type (WT), Δ*ydj1*, Δ*hsp104,* and Δ*ydj1*Δ*hsp104* cells after 66 h of expressing EGFP‐A42 (A42) or harboring the corresponding empty vector controls (ev). Dot plots show all data points along with the mean (bar) ± SD *n* = 6 biologically independent cultures. ***P < 0.001. ANOVA with Tukey’s *post hoc* test.HImmunoblot of whole‐cell extract (WCE) of wild‐type (WT) yeast cells after 16 h of expression of EGFP‐A42 and Hsp104‐FLAG using FLAG antibody (FLAG) and corresponding vector controls (ev). The Hsp104‐FLAG is ~102 kDa. GAPDH is used as a loading control. Green star marks an unspecific band.IImmunoblot of whole‐cell extract (WCE) of wild‐type and Δ*ydj1* cells expressing EGFP‐A42 (A42), EGFP‐A42m2 (m2), or EGFP only (ev) using Hsp104‐specific antibody. Pgk1 is used as a loading control. Source data are available online for this figure.

Immunoblot time series of EGFP‐A42‐expressing strains WT, Δ*ydj1,* and Ydj1 overexpression indicated that Ydj1 stabilized EGFP‐A42 and its heat‐sensitive low‐n oligomer (EGFP‐A42 tetramer marked with star) content (Fig EV3A–D). We therefore assessed whether Ydj1 stabilized the different forms of Abeta42 peptides preventing their turnover or, alternatively, enhanced *de novo* synthesis of the EGFP‐Abeta42 fusion protein. Therefore, we added the translation inhibitor cycloheximide to the yeast culture to interrupt *de novo* synthesis of proteins and monitored the decay of EGFP fluorescence intensity over time via flow cytometry, which is expected to correlate with the kinetics of cellular EGFP‐A42 degradation (Fig 3I, Appendix Fig S2C). EGFP‐A42 levels were stabilized upon Ydj1 overexpression, while deletion of *YDJ1* accelerated EGFP‐A42 degradation (Fig 3I). Next, we wanted to evaluate if the presence of Ydj1 affects low‐n oligomer formation and stability and therefore performed immunoblot analysis to assess the tetramer/monomer ratio of EGFP‐A42. Upon *YDJ1* deletion, the tetramer/monomer EGFP‐A42 ratio decreased significantly compared to wild‐type cells both after 16 and 32 h of expression (Figs 3J and EV3A and B, Appendix Fig S2A). In contrast, overexpression of Ydj1 stabilized EGFP‐A42 and its oligomers (Figs 3K and EV3C and D, Appendix Fig S2B) and, tended to increase the tetramer/monomer ratio after 32 h of expression (Fig 3K).

As deletion of *YDJ1* might change the profiles of other HSPs, we examined the yeast disaggregase Hsp104, previously shown to resolve amyloid aggregates (DeSantis *et al*, 2012) and to cooperate with Hsp40 and Hsp70 for dissolving and renaturing the aggregated proteome after environmental stress (Shorter & Lindquist, 2008). Indeed, our experiments revealed increased Hsp104 levels in the absence of Ydj1 (Fig EV3I). However, neither overexpression of Hsp104‐FLAG nor combined deletion of *HSP104* and *YDJ1* changed Abeta42 toxicity in the EGFP‐A42‐expressing yeast model (Fig EV3F–I).

The Abeta42‐stabilizing effect of Ydj1 could be confirmed in Kar2‐A42‐expressing yeast cells, again showing stabilization of low‐n oligomers (Fig EV2C and D). Inspection of Abeta42 localization by cellular fractionation in this model revealed Abeta42 deposition in both mitochondrial and microsomal fraction, which can be explained by the initial Kar2‐driven expression of Abeta42 toward the ER/secretory pathway. Importantly, the oligomer distribution differed between mitochondrial‐ and microsomal‐enriched fraction in spite of a moderate microsomal contamination in mitochondrial fractions (Fig EV2E). Evidently, high‐n oligomers were enriched in microsomal fraction, whereas the dodecamer and some low‐n oligomers were rather detected in mitochondrial fraction.

In sum, Ydj1 was found to be crucial for Abeta42 translocation to or to the proximity of mitochondria and appears to prevent the formation of large aggregates, while it favors the stability of low‐n oligomeric mitochondria‐localized forms of Abeta42.

### The human homologue of Ydj1, DnaJA1, re‐establishes Abeta42 toxicity in *YDJ1*‐deleted yeast and interacts with Abeta42

**Figure 4:**
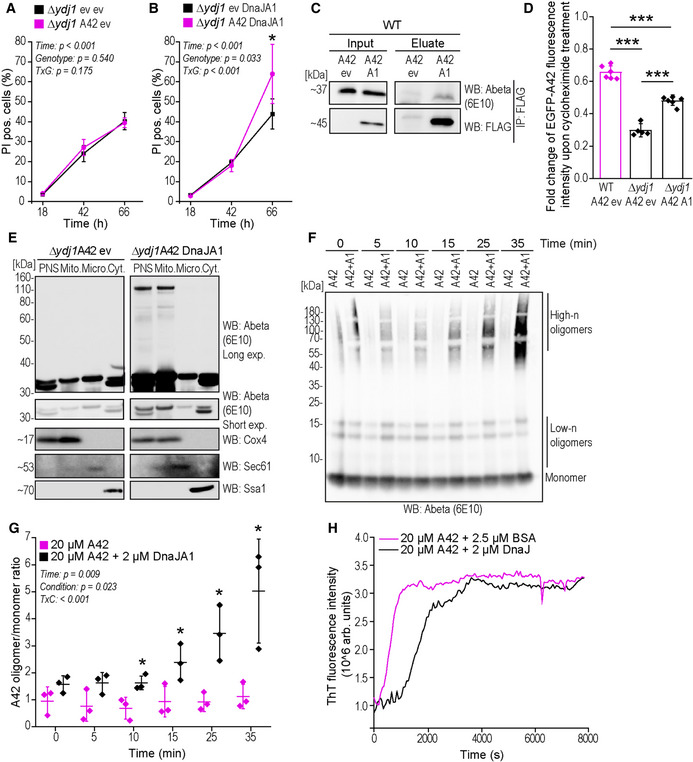
Human homologue of Ydj1, DnaJA1, modulates Abeta42 species formation and complements Abeta42 toxicity phenotypes in the absence of Ydj1 A, BQuantification of propidium iodide (PI)‐staining positive cells indicating cell death at indicated time points of expression of EGFP‐A42 (A42) Δ*ydj1* cells with co‐expression of DnaJA1‐FLAG (DnaJA1) (B) or corresponding empty vector controls (ev) (A). Mean ± SD *n* = 4–6 biologically independent cultures. Comparisons by two‐way ANOVA (mixed design) followed by simple main effects (*P < 0.05, versus control).CImmunoblot (WB) of whole‐cell extract (Input) and eluate of FLAG‐tagged DnaJA1 immunoprecipitation (IP: FLAG) of wild‐type strain (WT) expressing EGFP‐A42 (A42) and co‐expressing DnaJA1‐FLAG (A1) using Abeta‐specific antibody (6E10) and FLAG antibody (FLAG).DAssay for protein degradation using cycloheximide (CH) to stall protein translation. EGFP fluorescence intensity of *YDJ1* deletion strain (*Δydj1*) and wild‐type (WT) cells expressing EGFP‐A42 (A42) only or co‐expressing DnaJA1‐FLAG (A1) was measured at two time points (t0 and t2, 2 h after CH administration) and normalized to t0. Dot plots show all data points along with the mean (bar) ± SD *n* = 6 biologically independent cultures. ***P < 0.001. ANOVA with Tukey’s *post hoc* test. See also Appendix Fig S2C.EImmunoblot (WB) of total cytoplasmic post‐nuclear supernatant (PNS), mitochondrial (Mito.), microsomal (Micro.), and cytosolic (Cyt.) fractions of *YDJ1*‐deleted cells (Δ*ydj1*) after 18 h expression of EGFP‐A42 (A42) only or co‐expressing DnaJA1‐FLAG (DnaJA1) using Abeta‐specific antibody (Abeta) 6E10 with long (Long exp.) and short time (Short exp.) exposure (two sections from one immunoblot). Cox4‐specific antibody is a marker for mitochondria, Sec61 for microsomes and Ssa1 was used to verify cytosolic fraction.FRepresentative immunoblot of synthetic Abeta42 oligomer formation monitored *in vitro* over the indicated time course with or without DnaJA1 (A1) and quantified by densitometry in (G).GQuantification of the oligomer‐to‐monomer ratio of synthetic Abeta42 with or without DnaJA1 obtained from immunoblots representatively shown in (F). Dot plots show all data points along with the mean (line) ± SD *n* = 3 biologically independent cultures. *P*‐values by two‐way ANOVA (mixed design) followed by simple main effects (*P < 0.05, versus A42 control). See also Appendix Fig S2D.HA42 beta‐sheet‐rich assembly formation monitored by increase in ThT fluorescence over time with BSA or with DnaJ. Data represent means of at least eight measurements. See also Appendix Fig S1E. Source data are available online for this figure.

In an attempt to complement the loss of Ydj1 by its human orthologue (Whitmore *et al*, 2020), we co‐expressed human DnaJA1 together with EGFP‐Abeta42 in Δ*ydj1* yeast cells. Strikingly, DnaJA1 was able to restore EGFP‐A42 cell death in the absence of Ydj1 (Fig 4A and B). As to be expected, DnaJA1 interacted with EGFP‐A42 in a pull‐down experiment from whole‐cell extracts of cells co‐expressing DnaJA1‐FLAG and EGFP‐A42 (Fig 4C). Similar to Ydj1, DnaJA1 stabilized EGFP‐A42 peptides in a degradation kinetics assay (Figs 4D and EV3E), and restored EGFP‐A42 tetramer formation in *Δydj1* yeast cells (Figs 4E and EV3B and E). Finally, DnaJA1 facilitated translocation of EGFP‐A42 to the proximity of mitochondria, as assessed by cellular fractionation followed by immunoblotting (Fig 4E).

To gain additional insights into the effects of the DnaJA1 chaperone on Abeta42 oligomerization, we monitored aggregation properties of synthetic Abeta42 *in vitro* in a time‐course experiment (Fig 4F and G). Immunoblot analysis allowed us to quantify oligomer‐to‐monomer ratios of Abeta42. Of note, both low‐ and high‐n oligomers of up to 180 kDa in size were visible in this assay, while higher fibrils and large aggregates were not detected. Abeta42 oligomerization was observed shortly after the beginning of the incubation period independently of the presence of DnaJA1 (Fig 4F). Nevertheless, the presence of DnaJA1 strongly accelerated oligomer (low‐ and high‐n) formation as shown by an increased total oligomer/monomer ratio (Fig 4G) as well as tetramer/monomer ratio (Appendix Fig S2D) throughout the time‐course experiment. To further elucidate amyloid aggregation, a widely used amyloid fibrillization assay employing Thioflavin T (ThT) was applied. In this assay, ThT detects beta‐strand structures in higher‐ordered Abeta oligomers and fibrils. Small soluble low‐n oligomers (such as di‐, tri‐, and tetramers) and large aggregates lack well‐defined beta‐strand structures and thus are not detected by ThT (Garai & Frieden, 2013). Unfortunately, this assay was not applicable to our conditions using human DnaJA1, because DnaJA1 strongly interacted with ThT itself (Appendix Fig S1F). Thus, we monitored aggregation of synthetic Abeta42 dependent on DnaJ (Hsp40) purified from *E. coli* (Fig 4H, Appendix Fig S1E). While synthetic Abeta42 mixed with BSA formed ThT‐detectable structures within few minutes of incubation (after ~400 s), addition of 2 µM DnaJ significantly delayed the formation of ThT fluorescent Abeta42 assemblies (Fig 4H, Appendix Fig S1E). The lag phase preceding ThT fluorescence boost typically involves formation of oligomers before fibril formation (Garai & Frieden, 2013). It therefore appears plausible that DnaJ interferes with one of the microscopic processes in the first phase of amyloid growth formation, presumably favoring soluble Abeta intermediates.

**Figure 5:**
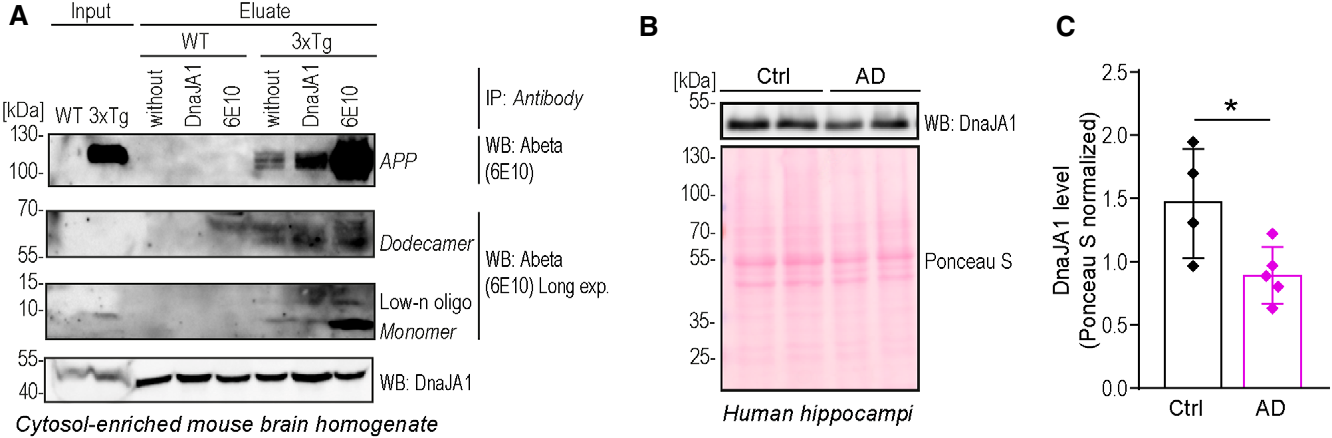
Human homologue of Ydj1, DnaJA1, interacts with Abeta42 and its oligomers in 3xTg mouse model and is altered in *post mortem* brain samples of AD patients Immunoblot of cytosol‐enriched cerebral tissue homogenate (Input) and eluate of Abeta42 or DnaJA1 immunoprecipitation (IP) using Abeta‐specific antibody (6E10) and DnaJA1‐specific antibody (DnaJA1), respectively, showing optimal and long exposure (Long exp.). Immunoprecipitation was performed using magnetic beads without antibody, or with DnaJA1 or 6E10 antibody mixed with cytosol‐enriched brain homogenates obtained from female (15 months old) wild‐type (WT) or 3xTg (PS1_M146V_/APP_Swe_/tau_P301L_) mice. Sections showing monomer and low‐n oligomers (low‐n oligo), dodecamer, and full‐length amyloid precursor protein (APP) are from one immunoblot.Representative immunoblot (WB) of DnaJA1 levels from the hippocampi of AD patients (AD) and aged non‐demented controls (ctrl). Ponceau S served as a loading control.Quantification by densitometry of DnaJA1 normalized to Ponceau S from the hippocampi of AD patients (AD) and aged non‐demented controls (ctrl). Representative immunoblot is shown in (B). Dot plots show all data points along with the mean (bar) ± SD *n* = 4–5 independent patients. *P < 0.05. Unpaired, two‐tailed *t*‐test. Source data are available online for this figure.

Most importantly, we show that DnaJA1 interacted with Abeta42 in *ex vivo* mouse 3xTg brain tissue homogenates (Fig 5A). The 3xTg mouse model expresses three transgenes: i) mutated Abeta precursor protein APP (APP_SWE_), ii) human four‐repeat tau P30lL mutation without amino terminal inserts (4R0N), and iii) human PSEN1 with M146V mutation, and is a widely used murine model of AD due to developing both main AD pathologies, namely extracellular Abeta depositions and tangle formations (Oddo *et al*, 2003). Performing a pull‐down assay with a cytosol‐enriched cell fraction of total brain homogenates, we detected the Abeta42 monomer, low‐n oligomers, the dodecamer, as well as full‐length APP in the eluate of beads bound to Abeta‐specific 6E10 antibody. Of note, DnaJA1 displayed non‐specific binding toward magnetic beads, complicating the interpretation of the 6E10 antibody‐based immunoprecipitation. Therefore, to determine if Abeta42 interacts with DnaJA1, we compared beads loaded with a DnaJA1‐specific antibody and beads without antibody. The amount of precipitated DnaJA1 consequently increased in DnaJA1‐antibody‐loaded beads compared to empty beads (Fig 5A). Similarly, the amount of co‐purifying APP, the Abeta dodecamer, and potentially low‐n oligomers increased, arguing for an interaction of Abeta protein sequence containing APP and Abeta oligomers with DnaJA1 in *ex vivo* brain homogenates (Fig 5A).

DnaJA1 is highly expressed in pyramidal cells of the hippocampus (https://www.proteinatlas.org/ENSG00000086061‐DNAJA1/tissue), the main site of human AD pathology (Davies *et al*, 1992) and has been found to be dysregulated in *post mortem* hippocampi of AD patients (Sorrentino *et al*, 2017). Using hippocampi of AD patients versus aged non‐demented controls, we could confirm differential protein levels of DnaJA1 in human *post mortem* brains by western blotting (Fig 5B and C).

### Depletion of *Drosophila* HSP40 reduces Abeta42‐induced toxicity in a fly model of AD

**Figure EV4:**
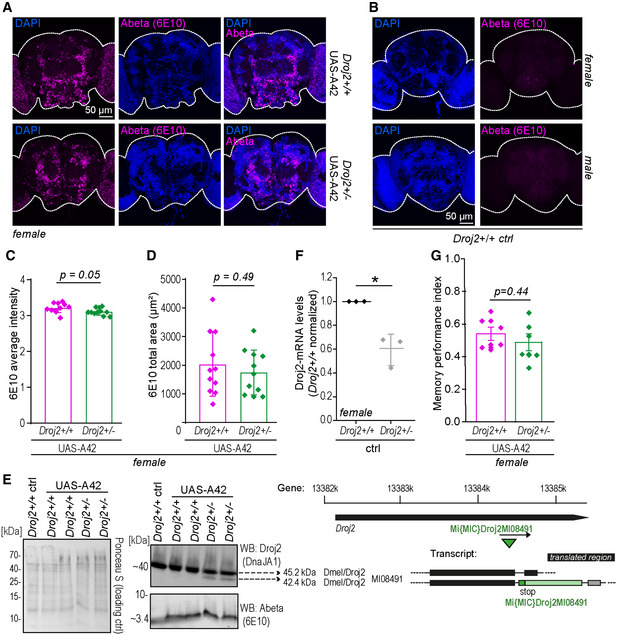
Abeta42‐mediated toxicity is dependent on the fly homologue of DnaJA1, Droj2, in an AD fly model A, BRepresentative confocal microscopy of (A) 10‐day‐old female fly brains immunostained with Abeta‐specific antibody (Abeta) (6E10) (magenta) and reference DNA staining with DAPI (blue) of *Droj2* knockdown flies (*Droj2* ^+/−^) and corresponding isogenic *w^1118^* wild‐type flies (*Droj2* ^+/+^) expressing human Abeta42 (UAS‐A42). (B) 10‐day‐old female and male fly brains immunostained with Abeta‐specific antibody (Abeta) (6E10) (red) and reference DNA staining with DAPI (blue) of corresponding isogenic *w^1118^* wild‐type control flies (*Droj2* ^+/+^ *ctrl*) not expressing human Abeta42.C, DAverage intensity (C) and total area (D) of Abeta (6E10) signal from fly brain confocal images representatively shown in Fig EV4A from 12 brains of *w^1118^* wild‐type (*Droj2* ^+/+^) and knockdown (*Droj2* ^+/−^) female flies. Dot plots show all data points along with the mean (bar) ± SD *n* = 12. Unpaired, two‐tailed *t*‐test.EFly Droj2 is recognized by specific antibody of the human homologue DnaJA1. Immunoblot analysis of fly heads using DnaJA1‐specific antibody (DnaJA1) and Abeta‐specific antibody (6E10) of 3‐ to 6‐day‐old Droj2 knockdown flies (*Droj2* ^+/−^) and corresponding isogenic *w^1118^* wild‐type flies (*Droj2* ^+/+^) with expression of human Abeta42 (UAS‐A42) or without, control (ctrl). Ponceau S was used as a loading control. Scheme of the Droj2 locus with the position of the Mi{MIC}Droj2MI08491 transposon. In the transcript, black rectangles indicate translated regions, lines indicate introns, and dashed lines indicate untranslated regions. Data are adopted from FlyBase.FqPCR analysis of Droj2‐mRNA levels of 3‐ to 6‐day‐old *Droj2* knockdown female flies (*Droj2* ^+/−^) without expressing human Abeta42 (ctrl) normalized to corresponding isogenic *w^1118^* wild‐type flies (*Droj2* ^+/+^). Reference gene is *Rpl32*. Dot plots show all data points along with the mean (line) ± SD *n* = 3 biologically independent experiments. *P < 0.05. One sample *t*‐test against 1.GAversive associative memory performance 2 min after training of aged (18 days old) female Droj2 knockdown flies (*Droj2* ^+/−^) and corresponding isogenic *w^1118^* wild‐type flies (*Droj2* ^+/+^) both expressing human Abeta42 (UAS‐A42) of six independent biological replicates. Dot plots show all data points along with the mean (bar) ± SD *n* = 6. Unpaired, two‐tailed *t*‐test. Source data are available online for this figure.

**Figure 6:**
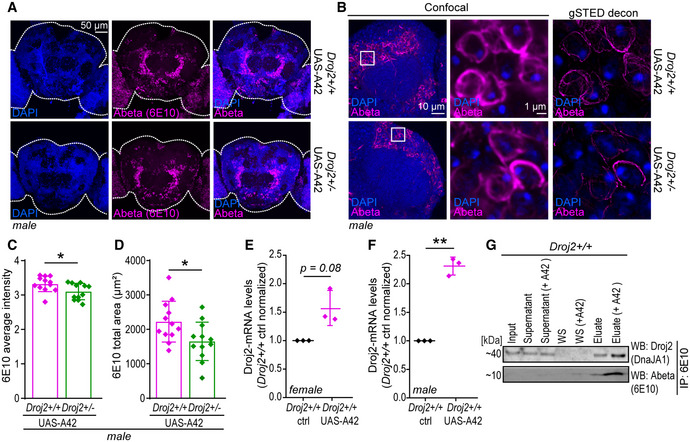
Droj2, fly homologue of DnaJA1, is upregulated in response to Abeta42 expression and its downregulation decreases neuronal Abeta accumulation ARepresentative confocal microscopy of 10‐day‐old male fly brains immunostained with Abeta‐specific antibody (Abeta) 6E10 (magenta) and reference nuclei staining with DAPI (blue) of Droj2 knockdown flies (*Droj2* ^+/−^) and corresponding isogenic *w^1118^* wild‐type flies (*Droj2* ^+/+^) expressing human Abeta42 (UAS‐A42). See also Fig EV4A and B.BRepresentative confocal and gSTED deconvolved (decon) images of Kenyon cells in 18‐day‐old male fly brains immunostained with Abeta‐specific antibody (Abeta) 6E10 (magenta) and reference nuclei staining with DAPI (blue) of Droj2 knockdown flies (*Droj2* ^+/−^) and corresponding isogenic *w^1118^* wild‐type flies (*Droj2* ^+/+^) expressing human Abeta42 (UAS‐A42).C, DAverage intensity (C) and total area (D) of Abeta (6E10) signal from confocal images representatively shown in (A) from 12 brains of *w^1118^* wild‐type (*Droj2* ^+/+^) and knockdown (*Droj2* ^+/−^) 10‐day‐old male flies. Dot plots show all data points along with the mean (bar) ± SD *n* = 12. *P < 0.05. Unpaired, two‐tailed *t*‐test. See also Fig EV4C and D.E, FqPCR analysis of Droj2‐mRNA levels of 3‐ to 6‐day‐old female (E) and male (F) flies expressing human Abeta42 (UAS‐A42) of *w^1118^* wild‐type flies (*Droj2* ^+/+^) normalized to corresponding isogenic *w^1118^* wild‐type flies without Abeta42 expression (*Droj2* ^+/+^ ctrl). Reference gene is *Rpl32*. Dot plots show all data points along with the mean (line) ± SD *n* = 3. **P < 0.01. One sample *t*‐test against 1.GImmunoprecipitation (IP: 6E10) of synthetic Abeta42 added to the fly head extract of *w^1118^* wild‐type (*Droj2^+/+^*) flies (Input). Abeta‐specific antibody 6E10 was used for Abeta42‐ and DnaJA1‐specific antibody for Droj2 immunoblot (WB) detection. Showing input, supernatant, supernatant after washing step (WS), and eluate. See also Fig EV4E. Source data are available online for this figure.

We went on examining the effects of this particular HSP40 on Abeta42 toxicity in a (*Drosophila melanogaster*) fly model of AD (Sekiya & Iijima, 2021). This model expresses the human Abeta42 protein sequence fused to the rat preproenkephalin signal peptide and targets Abeta42 to the secretory pathway. AD‐like phenotypes, including deficits in olfactory memory, have been reported previously (Iijima *et al*, 2008). To test the effect of HSP40 in this model, we generated a strain by combining a mutated form of the Ydj1 homologue Droj2 (*Drosophila* DnaJ‐like‐2) (Venken *et al*, 2011) and the pan‐neuronal expression of nSyb‐*GAL4*‐driven Abeta42 fly strain (*Droj2* ^+/−^ UAS‐A42). This heterozygote mutated form of *Droj2* led to a reduction in mRNA levels to 60% as well as less protein compared to the wild‐type (*Droj2^+/+^* UAS‐A42) control (Fig EV4F). Confocal and time‐gated stimulated emission depletion (gSTED) microscopy of 6E10 (Abeta‐specific) antibody‐stained brains revealed typical Abeta deposits in both strains expressing Abeta42 (Iijima *et al*, 2008) (Figs 6A and B, and EV4A and B). To assess if reduced levels of Droj2 influence Abeta burden across the brain in mid‐aged flies, we quantified the average intensity and total area of Abeta (6E10) signals using confocal images. Significant reduction in Abeta accumulation was detected in *Droj2* knockdown male flies (*Droj2* ^+/−^ UAS‐A42) (Fig 6C and D), albeit unchanged Abeta42‐mRNA levels (Appendix Fig S1G). However, this effect was moderate to absent in female flies (Fig EV4C and D), indicating sex‐specific effects. Monitoring Droj2‐mRNA levels of AD (Abeta42‐expressing) flies compared to the wild‐type (*Droj2* ^+/+^) control flies revealed an increase in Droj2 in the presence of Abeta42 in young flies (Fig 6E and F). We also asked if Droj2 can bind to Abeta42. Indeed, Droj2 interacted with synthetic Abeta42 in a pull‐down experiment from wild‐type (*Droj2* ^+/+^) fly head extracts (Fig 6G, Appendix Fig S1H) using a human DnaJA1‐specific antibody, which we confirmed to cross‐react with *Drosophila* Droj2 (Fig EV4E). Of note, Droj2 displayed non‐specific binding to magnetic beads, nevertheless affinity purification revealed a strong increase in Droj2 in the presence of synthetic Abeta42 (Fig 6G).

**Figure 7:**
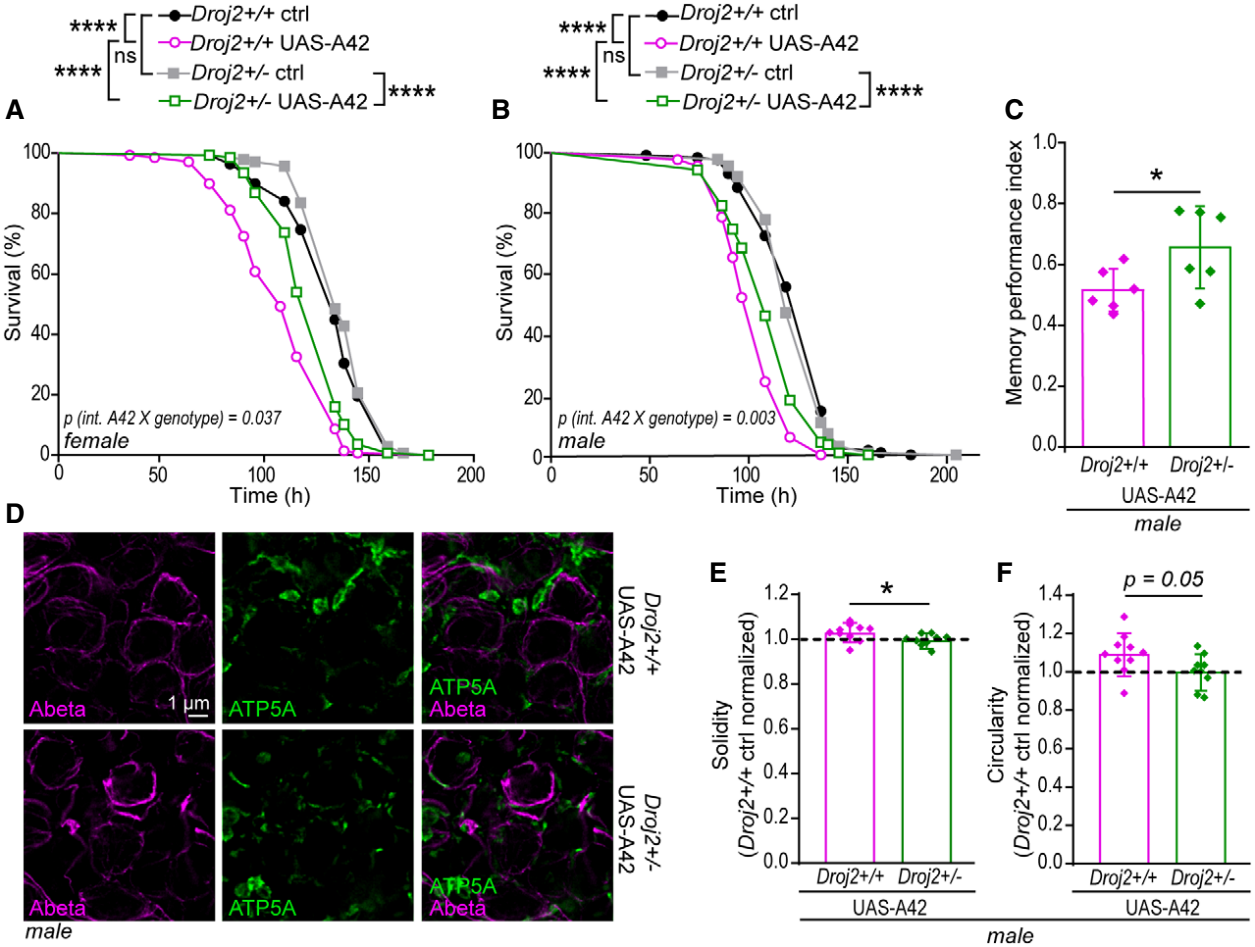
Abeta42‐induced mortality, cognitive impairment, and mitochondrial morphology changes depend on the fly homologue of DnaJA1, Droj2 A, BSurvival of female (A) and male (B) *w^1118^* wild‐type flies (*Droj2* ^+/+^) and Droj2 knockdown flies (*Droj2* ^+/−^) with expression of human Abeta42 (UAS‐A42) or control flies without expression (ctrl), upon supplementation of sugar (10% sucrose) with 20 mM MnCl_2_. Survival was determined at indicated time points. *n* = 6 with 100–120 flies per experiment. The indicated *P*‐value refers to the interaction (int.) term of a Cox proportional hazards model comparing Abeta42 toxicity (UAS‐A42 versus ctrl) and Droj2 expression (*Droj2* ^+/+^ versus *Droj2* ^+/−^) as main factors. The following pairwise comparisons of the indicated groups survival were done by log rank test (****P < 0.0001; ns, *P* > 0.05). See also Appendix Fig S1I–K.CAversive associative memory performance 2 min after training of aged (18 days old) male Droj2 knockdown flies (*Droj2* ^+/−^) and corresponding isogenic *w^1118^* wild‐type flies (*Droj2* ^+/+^) both expressing human Abeta42 (UAS‐A42) of six independent biological replicates. Dot plots show all data points along with the mean (bar) ± SD *n* = 6. *P < 0.05. Unpaired, two‐tailed *t*‐test. See also Fig EV4G.DRepresentative gSTED deconvolved images of Kenyon cells in 15‐day‐old male fly brains immunostained with Abeta‐specific antibody (Abeta) 6E10 (magenta) and mitochondrial marker ATP5A‐specific antibody (ATP5A, green) of Droj2 knockdown flies (*Droj2* ^+/−^) and corresponding isogenic *w^1118^* wild‐type flies (*Droj2* ^+/+^) expressing human Abeta42 (UAS‐A42).E, FSolidity (E) and circularity (F) normalized to corresponding wild‐type *Droj2^+/+^* control (without Abeta42 expression, dashed line) of ATP5A‐stained mitochondria from fly brain gSTED deconvolved images representatively shown in (D) from 8 to 10 brains of *w^1118^* wild‐type (*Droj2* ^+/+^) and knockdown (*Droj2* ^+/−^) 15‐day‐old male flies expressing human Abeta42 (UAS‐A42). Dot plots show all data points along with the mean (bar) ± SD *n* = 8–10. *P < 0.05. Unpaired, two‐tailed *t*‐test. See also Fig EV5D–H.

To address the consequences of Droj2 depletion on Abeta‐induced toxicity, we decided to monitor survival under manganese stress, a known environmental risk factor for AD, in order to accelerate Abeta42 toxic effects (Burton & Guilarte, 2009; Tong *et al*, 2014). Abeta42 significantly enhanced manganese‐induced death in both male and female wild‐type (*Droj2* ^+/+^) flies but was less toxic in the *Droj2* knockdown (*Droj2* ^+/−^) flies (Fig 7A and B, Appendix Fig S1I–K). Since Abeta42‐expressing flies develop memory deficits (Iijima *et al*, 2008), we aimed to investigate whether Droj2 reduction can mitigate loss of memory in aged AD flies. Indeed, Droj2 reduction significantly improved short‐term olfactory memory (STM) in aged (18‐day‐old) Abeta42‐expressing male, but not female flies (Figs 7C and EV4G), correlating with Abeta burden in male and female flies and again, suggesting sex‐specific effects in this model system (Figs 6C and D, and EV4C and D).

**Figure EV5:**
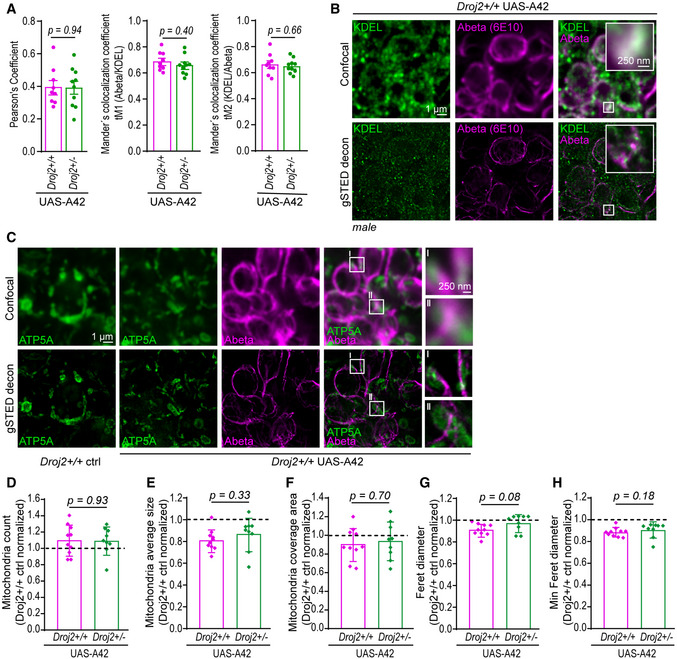
Abeta42‐mediated toxicity is dependent on the fly homologue of DnaJA1, Droj2, in an AD fly model APearson’s correlation coefficient and Manders’ co‐localization Coefficients of endoplasmatic reticulum protein KDEL and Abeta analyzed on confocal images of Kenyon cells in 15‐day‐old male fly brains immunostained with Abeta‐specific antibody (Abeta) 6E10 and KDEL‐specific antibody in Droj2 knockdown flies (*Droj2* ^+/−^) and corresponding isogenic *w^1118^* wild‐type flies (*Droj2* ^+/+^) with expression of human Abeta42 (UAS‐A42). Dot plots show all data points along with the mean (bar) ± SD *n* = 10. Unpaired, two‐tailed *t*‐test.BRepresentative confocal and gSTED deconvolved (decon) images of Kenyon cells in 15‐day‐old male fly brains immunostained with Abeta‐specific antibody (Abeta) 6E10 (magenta) and endoplasmic reticulum protein KDEL‐specific antibody (KDEL, green) of Droj2 knockdown flies (*Droj2* ^+/−^) and corresponding isogenic *w^1118^* wild‐type flies (*Droj2* ^+/+^) expressing human Abeta42 (UAS‐A42).CRepresentative confocal and gSTED deconvolved (decon) microscopy of Kenyon cells in 15‐day‐old male fly brains immunostained with Abeta‐specific antibody (Abeta) 6E10 (magenta) and mitochondrial marker ATP5A‐specific antibody (ATP5A, green) of *w^1118^* wild‐type flies (*Droj2* ^+/+^) expressing human Abeta42 (UAS‐A42).D–HCounts of mitochondria (D), mitochondria average size (E), mitochondria coverage area (F), Feret diameter (G), and min Feret diameter (H) of ATP5A‐stained mitochondria from fly brain gSTED deconvolved images representatively shown in Fig 7C from 9 to 10 brains of *w^1118^* wild‐type (*Droj2* ^+/+^) and knockdown (*Droj2* ^+/−^) male flies expressing human Abeta42 (UAS‐A42). Dot plots show all data points along with the mean (bar) ± SD *n* = 9–10. Unpaired, two‐tailed *t*‐test or Mann–Whitney test.

In order to assess the effects of Droj2 downregulation on cellular Abeta distribution in male flies, we utilized gSTED microscopy, a super‐resolution technique allowing a lateral resolution of approximately 40 nm (Pooryasin *et al*, 2021). Double staining for the ER marker KDEL and 6E10 antibodies in fly whole‐mount brains revealed partial co‐localization of Abeta with ER in Kenyon cells (intrinsic neurons of the Mushroom Body) based on Pearson's and Mander's coefficients, in line with the expression of Abeta42 within the ER/secretory pathway in this model. No significant differences in co‐localization were observed between wild‐type and Droj2 knockdown (Fig EV5A and B). Comprehensive gSTED assessment of Abeta cellular localization relative to the ATP synthase subunit ATP5A (an inner mitochondrial membrane marker) revealed mitochondria–Abeta contact sites (Fig EV5C). This observation goes in line with our cell fractionation data obtained utilizing Kar2‐Abeta42 yeast model (Fig EV2E). Even though we detected Abeta in close proximity to mitochondria, a quantitative analysis to determine whether this could be affected by Droj2 is technically challenging. This would require to discriminate ER–mitochondria contact sites, considering the high degree of Abeta/ER co‐localization observed in both Abeta‐expressing wild‐type and Droj2 knockdown flies. Previous reports showed Abeta‐induced alterations in mitochondrial morphology in Mushroom Body neurons (Wang & Davis, 2021), we therefore decided to continue analyzing mitochondrial morphology changes upon Droj2 downregulation by gSTED microscopy. In Abeta‐expressing flies, Droj2 knock‐down (*Droj2* ^+/−^ compared to *Droj2* ^+/+^) decreased solidity (Fig 7D and E) (Napoli *et al*, 2021) and circularity (Fig 7D and F) (Kalkhoran *et al*, 2017), resulting in a mitochondrial morphology more similar to wild‐type flies (Figs 7D–F and EV5D–H). These, at first sight rather moderate effects are in line with the 40% reduction in Droj2 mRNA in the *Droj2* ^+/−^ strain.

Taken together, our results demonstrate phylogenetic conservation of the capacity of this particular Hsp40 to promote Abeta42 oligomer stabilization both *in vivo* and *in vitro* and to exacerbate Abeta42 toxicity, cellular morphological changes, and memory loss *in vivo* in different AD‐relevant model systems.

## Discussion

In this study, using two independent yeast and one established fly model of AD, we identified the Hsp40 protein Ydj1/Droj2 to drive Abeta42 toxicity. Our *in vivo* analysis was accompanied by *in vitro* and *ex vivo* experiments showing Abeta interaction with human and mouse DnaJA1 protein, as well as dysregulation of this chaperone in *post mortem* human brains pointing to an important role of DnaJA1 in AD.

To specifically investigate the consequences of intracellular Abeta42, we established a yeast model expressing EGFP‐A42 directly to the cytosol. Here, expression of human Abeta42 provoked typical hallmarks of Abeta toxicity, such as oxidative stress and cell death, confirming previous findings on aging yeast (Chen & Petranovic, 2015). In addition, we observed that intracellular Abeta42 forms different multimeric assemblies within the cell, ranging from small oligomers to large aggregates. Localization studies revealed that Abeta42 translocates to or to the proximity of mitochondria, consistent with an impairment of ATP production and elevated oxidative stress. Oxidative damage, mitochondrial dysfunction, and neuronal cell death have all been recognized during AD pathogenesis (LaFerla *et al*, 2007; Sorrentino *et al*, 2017). Our observations are in accord with previous studies showing Abeta42‐dependent decline in ATP synthesis and dysfunction of the oxidative phosphorylation system (Rhein *et al*, 2009; Bobba *et al*, 2013). Besides, we obtained evidence that Abeta42 triggers oxidative stress leading to cell death only if mitochondria are respiration competent. This goes in line with previous findings that the key enzymes mediating the Warburg effect play a central role in mediating neuronal resistance to Abeta by decreasing mitochondrial activity (Newington *et al*, 2011, 2012). Another striking outcome revealed by this study is the presence of oligomeric Abeta at mitochondria in both (EGFP‐A42‐ and Kar2‐A42‐based) yeast models, despite the fact that the Kar2‐A42 model directs Abeta42 to the secretory pathway. Utilizing the Kar2‐Abeta yeast model, Chen and Petranovic previously demonstrated Abeta‐induced mitochondrial dysfunction (Chen & Petranovic, 2015). In line with these findings, mitochondrial Abeta was detected in human AD samples (Hansson Petersen *et al*, 2008) as well as in brain tissue of 3xTg mouse line, which also shows age‐dependent decline in mitochondrial function (Espino de la Fuente‐Muñoz *et al*, 2020).

Using two independent screening approaches, we identified the yeast HSP40 family member, Ydj1, as a crucial mediator of Abeta42 toxicity. Ydj1 has been described as a co‐chaperone supporting the ATPase activity of HSP70 chaperone proteins and their interaction with polypeptide substrates (Walsh *et al*, 2004). Nevertheless, Ydj1 can also bind substrates independently of HSP70, preventing their aggregation *in vitro* (Langer *et al*, 1992; Cyr, 1995; Meacham *et al*, 1999). This goes in line with our *in vitro* data where DnaJA1 interferes with Abeta oligomerization pattern. Also, in support of an HSP70‐independent function of Ydj1 to promote Abeta toxicity, we failed to mimic the effects of *YDJ1* deletion by the Hsp40/Hsp70 complex inhibitor 116‐9e that binds Hsp70 and prevents the Hsp70 activating function of Ydj1, while it does not interfere with its Hsp70‐independent function (Wisén *et al*, 2010; Sluder *et al*, 2018). Ydj1/DnaJA1 is active during cellular stress responses and responsible for protein folding and re‐folding, suppression of protein aggregates, and mitochondrial as well as ER protein translocation (Caplan *et al*, 1992; Glover & Lindquist, 1998; Jores *et al*, 2018). We provide evidence that Ydj1 and its human homologue, DnaJA1 (Qiu *et al*, 2006), interact with Abeta42, thereby influencing the aggregation and oligomerization properties of Abeta42.

Moreover, Ydj1 and DnaJA1 appear to be required for the presence of oligomeric Abeta42 at mitochondria and for inducing mitochondrial dysfunction. Consequently, deletion of *YDJ1* reduced Abeta42 translocation to mitochondria and rescued mitochondrial defects, including ROS generation and cell death. This echoes previous findings in a fly model, showing that the amyotrophic lateral sclerosis and frontotemporal lobar degeneration‐associated FUS protein can be transported to mitochondria via HSP60, thereby inducing mitochondrial impairment and cell death (Deng *et al*, 2015). Another study reported that Hsp60 is required for mitochondrial APP and Abeta mislocalization in a neuronal cell line carrying APP Swedish mutation (Walls *et al*, 2012). Because APP to Abeta processing has also been reported at mitochondria‐associated membranes (MAMs), it will be important to test whether Hsp40 or Hsp60 affects Abeta’s interaction with mitochondria at the site of or by influencing MAMs (Del Prete *et al*, 2017). Interestingly, mitochondria may be essential for turnover of unfolded, cytosolic proteins, as previously suggested by Ruan *et al* (2017). This could explain why aggregate‐prone peptides such as Abeta42 are (actively) translocated to mitochondria and may serve as a cellular defense mechanism against various proteinopathies. It is tempting to speculate that upon declining mitochondrial function in aging or disease, mitochondria might be overwhelmed by protein aggregates, resulting in mitochondrial damage, and thus mitochondria‐associated cell death.

Using an AD fly model, we demonstrated phylogenetic conservation of our findings. Depletion of the Ydj1/DnaJA1 fly homologue, Droj2, improved mitochondrial morphology, diminished Abeta42‐mediated toxicity upon manganese stress, and partly re‐established Abeta42‐induced memory loss in aged flies in a sex‐specific manner. Droj2 was significantly upregulated in response to Abeta42 expression in males, but to a lesser extent in female flies. Moreover, *Droj2* knockdown male flies accumulated less Abeta burden during aging compared to Droj2‐proficient controls, once again an effect that was less pronounced or absent in females. One may speculate that the fine tuning of Hsp40 levels could be responsible for the observed sex differences, however, future studies are needed to corroborate this.

Dimers of DnaJA1 have previously been shown to bind to their unfolded clients containing large hydrophobic and aromatic residues close to acidic residues (Terada & Oike, 2010). These motif characteristics match the Abeta42 peptide sequence (Soto *et al*, 2002). In view of this, we revealed in our study that Ydj1 and DnaJA1 interact with Abeta42. Most importantly, Ydj1 did not bind to the non‐toxic Abeta42m2, which contains mutations in the two aggregation important regions of Abeta42. Furthermore, stabilization of Abeta42 oligomers was supported by our *in vitro* data, which indicate that human DnaJA1 accelerate the formation of Abeta42 oligomers as well as that purified DnaJ from *E*. *coli* delays Abeta42 aggregation, presumably by inhibiting one of the biophysical processes involved in formation of ThT‐detectable amyloid fibrils. This is interesting because targeted inhibition of fibril elongation could induce abundance of soluble intermediates, which in turn increase cytotoxicity (Scheidt *et al*, 2019). Of note, Carnini *et al* (2012) also investigated the association between HSP40/DnaJA’s and Abeta in *in vitro* cell culture, illustrating cell line‐specific HSP40‐dependent influence of Abeta peptide stability. While HSP40 transfection into catecholaminergic a‐differentiated (CAD) cells decreased cellular levels of Abeta, it increased Abeta levels in hippocampal cultures, demonstrating a possible pathogenic role of HSP40 in AD (Carnini *et al*, 2012). The authors speculated that in specific disease conditions, HSP40 would protect Abeta42 from degradation, thereby favoring Abeta42 accumulation and AD progression (Carnini *et al*, 2012). In parallel, another study tested HSP40 effects on the AD‐associated protein tau showing that overexpression of DnaJA1 can favor both tau clearance and stabilization dependent on Hsp70 levels in M17 neuroblastoma cells (Abisambra *et al*, 2012). A recent study reported similar findings, whereby inducing DnaJA1 activity by CRBN (endogenous substrate of cerebelon) downregulation decreased phosphorylation and aggregation of tau, which was detected *in vivo* and *in vitro* (Akber *et al*, 2021). Furthermore, Abisambra *et al* also demonstrated DnaJA1‐induced polyQ clearance, while alpha‐synuclein stability was unaffected in a model of Parkinson’s disease. This goes in line with our observation that *YDJ1* does not influence alpha‐synuclein toxicity in yeast, indicating differential roles of Ydj1/DnaJA1 toward some clients implicated in proteinopathies. *Vice versa*, divergent functions of Hsp40 chaperones have been reported. For instance, opposite actions of DnaJA1 and DnaJB6, another Hsp40 family member, were demonstrated in an *in vitro* model of Huntington´s disease (Rodríguez‐González *et al*, 2020). Interestingly, DnaJB6 has been shown to inhibit the primary nucleation of Abeta40 oligomer formation as well as fibril formation of Abeta42 (Månsson *et al*, 2014; Österlund *et al*, 2020). It will be interesting to study commonalities and potential differences in DnaJA1 and DnaJB6 regarding their role in Abeta toxicity.

Recent progress in understanding amyloid kinetics enabled resolving the effects of well‐known chaperones on specific microscopic stages of Abeta42 aggregation. Inhibitory effects of the chaperone clusterin on fibril elongation have been reported (Scheidt *et al*, 2019). Simultaneously, another group provided evidence of clusterin involvement in early stages of AD using the 5×FAD mouse model. They postulated that clusterin binding to Abeta42 oligomers might protect soluble toxic intermediates from enzymatic degradation and thus stabilize them (Oh *et al*, 2019). In addition, another study performed by Stege and colleagues demonstrated that the small HSP alphaB‐crystallin is able to prevent Abeta fibrillization, stabilizing non‐fibrillar neurotoxic species (Stege *et al*, 1999).

Interestingly, DnaJA1 is downregulated in *post mortem* brain samples of patients who suffered from AD (Abisambra *et al*, 2012; Sorrentino *et al*, 2017) and has been postulated as one of the major AD‐ and MCI‐associated genes using bioinformatic meta‐analysis (Tao *et al*, 2020). In a similar fashion to our results, this particular chaperone was upregulated as part of an early heat shock response in spinocerebellar ataxia‐7 (SCA7) patient‐derived fibroblasts (Scholefield *et al*, 2014) and in young SCA7 transgenic mouse model, while it was downregulated in older SCA7 mice (Chou *et al*, 2010). Age‐dependent regulation of DnaJA1 levels in cortical tissue has been already reported with highest expression in teenagers and young adults (16–23 years) (Breen *et al*, 2018). At the same time, oligomeric Abeta shows temporal profiles throughout aging, some being already present at early age long before first symptoms occur (Lesne, 2014). Accordingly, AD progression most likely starts at least two or three decades before actual diagnosis. These data together with our results suggest that DnaJA1 plays a crucial role in AD, but it remains yet to be determined at which stage of this malady DnaJA1 may exert its pathological actions.

Targeted regulation of specific HSPs, which can prevent or promote aggregation of misfolded proteins, might serve as a defense response against proteotoxicity. This makes such chaperones highly attractive for therapeutic targeting against neurodegenerative diseases. However, our study shows that it is of the utmost importance to dissect their specific role in proteinopathies thoroughly and to consider both protective and toxic effects.

## Materials and Methods

### Yeast strains, media, and plasmids

Experiments were carried out in BY4741 (MATa *his3*Δ*1 leu2*Δ*0 met15*Δ*0 ura3*Δ*0*) and respective null mutants, obtained from Euroscarf. For generating strains with depletion of mtDNA (Rho^0^), BY4741 wild‐type cells were grown in full medium containing 10 µg/ml ethidium bromide for 3 days. The resulting respiratory deficiency was confirmed by complete lack of growth on obligatory respiratory medium (glycerol as the sole carbon source). For most experiments with plasmid harboring yeast strains, at least six different clones obtained after plasmid transformation were tested separately to rule out clonal variations, with the exception of the genetic screen, where three individual clones were processed. In general, strains were grown on SC medium containing 0.17% yeast nitrogen base (BD Difco), 0.5% (NH_4_)_2_SO_4_, and 30 mg/l of all amino acids (except 80 mg/l histidine and 200 mg/l leucine), 30 mg/l adenine, and 320 mg/l uracil with 2% glucose (SCD). For induction of expression of plasmid‐encoded EGFP‐, Kar2, and FLAG constructs, cells were grown in SCD medium lacking histidine and/or uracil (in presence of pESC‐his and/or pESC‐ura plasmids, respectively) to logarithmic phase and shifted by centrifugation at 3,500 *g* for 5 min and resuspension to fresh 2% galactose SC medium (SCG) lacking the same amino acids to maintain selective pressure for plasmid(s). Vector constructs containing Abeta peptides were based on pESC vectors (Agilent Technologies, formerly obtained from Stratagene) that contained yEGFP3 (Cormack *et al*, 1997) (hereafter referred to as EGFP) N‐terminally located within the multiple cloning site (MCS) that also coded for a linker sequence 5′‐CGAATTCAACCCTCACTAAAGGGCGGCCGCACTAGT‐3′ to guarantee proper folding of both EGFP and inserted peptides (A42, C57, A40, and A42m2). To generate EGFP‐containing vectors, EGFP was amplified by PCR using pUG35‐ura (Gueldener, U and Hegemann, J.H) as template (see Appendix Table S1 for primers), cut with *EcoRI* and ligated into pESC‐his to generate the empty vector (pESC‐his‐EGFP), where a stop codon is present in frame with EGFP within the MCS. A cloning vector (pESC‐his‐EGFP_G) having an additional guanine inserted after the EGFP (see Table 1 for alternative primers) and thus changing the reading frame of the MCS to omit the STOP codon was generated. To construct A42, C57, A40, and A42m2 EGFP fusion proteins, inserts were amplified by PCR (see Appendix Table S1 for primers) using cDNA generated from human neuronal cells and plasmid A42m2 (Aβm2‐MRF), a kind gift of Susan Liebman (Bagriantsev & Liebman, 2006), respectively. PCR fragments were cut with *Spe*I and *Cla*I and ligated into pESC‐his‐EGFP_G. To generate yeast Ydj1‐FLAG and human DnaJA1‐FLAG, inserts were amplified using yeast chromosomal DNA and cDNA from human neuronal cells, respectively, cut with *Spe*I and *Cla*I and ligated into pESC‐ura (Agilent Technologies). Kar2‐A42 was obtained from p416 GPD‐Kar2‐Aβ42, a kind gift of Dina Petranovic (Chen & Petranovic, 2015) and cloned in pESC‐his vector using *ClaI* and *SpeI*. All constructs were validated by sequencing. All primers used for cloning are listed in Supplemental Experimental Procedures (Appendix Table S1).

### Yeast cultures, flow cytometry, oxidative stress levels, and test for apoptotic markers

For yeast experiments, 200 µl SCD medium lacking appropriate amino acids for plasmid selection in deep well plates (Bel‐Art, Cat. No. 378600000) was inoculated with 5–10 µl fresh overnight cultures to reach cell densities of approximately 1 × 10^6^ cells/ml. Cells were grown at 28°C and 320 rpm for 5 h and then shifted to SCG medium to induce plasmid‐based protein expression. Plates were sealed with gas permeable membranes (Excel Scientific, Cat. No. B100) throughout the course of the experiment. Aliquots of ~1 × 10^7^ cells were harvested to perform tests for oxidative stress and cell death markers at indicated time points over a period of 3 days. Data were either analyzed from 42 or 66 h time point depending on when empty vector (EGFP‐expressing) cells reached ~10% cell death or the complete time course is shown. Tests for apoptotic (annexin V staining) and necrotic (propidium iodide, PI staining) markers, as well as markers for oxidative stress (dihydroethidium, DHE, to ethidium, Eth, conversion assay), were performed as described previously (Kainz *et al*, 2017). Instead of FITC‐labeled annexin V, annexin V Alexa Fluor 647 (Invitrogen, Cat. No. A23204) was used giving rise to red fluorescence that was compatible both with the endogenous green fluorescence derived from EGFP fusion proteins and PI fluorescence. For quantification using flow cytometry (BD LSRFortessa and BD FACSAria IIu), 30,000 cells were evaluated and analyzed with BD FACSDiva software. Unstained and single‐stained samples served as controls for setting gates and proper compensation of respective channels (EGFP detection, 530/30 nm, excited by 488 nm laser; Eth detection, 695/40 nm, excited by 488 nm laser; PI detection, 695/40 nm, excited by 488 nm laser, annexin V Alexa Fluor 647 detection, APC channel, excited by 633 nm laser). DHE>Eth. positive cells were defined by gates that included both strong and weak fluorescence populations, allowing the detection of both dead cells and cells exhibiting oxidative stress.

### Genetic screen

The genetic screen was performed by assessing the number of DHE>Eth. positive cells after 42 h culture time of yeast cultures of different gene deletion strains expressing EGFP alone (vector control) or EGFP‐A42. The EGFP‐A42 to EGFP ratio of the fraction of DHE>Eth. positive cells served as a measure of Abeta42 toxicity (*toxicity ratio*). Three different clones (individual plasmid transformants) of each strain were aged separately and pooled before flow cytometry. This procedure was repeated three times in independent experiments (initial screen) and all deletion strains which showed a toxicity ratio of 1.0–1.3 (wild‐type cells on average displayed a ratio of 2) were classified as potential hits (i.e., reducing Abeta42 toxicity by ≥ 70%) and analyzed again (replication screen). Subsequently, all remaining hits were analyzed in at least three independent experiments and the data from all replicates were pooled for final data analysis.

### ATP assay

To determine ATP levels of yeast cells, intracellular metabolites were extracted using hot ethanol. Briefly, 1 × 10^8^ cells were harvested after 42 h culture time at 16,000 *g* for 2 min at room temperature (RT) and resuspended in 0.5 ml of boiling ethanol (75% ethanol, 10 mM (NH_4_)_2_SO_4_) and incubated in a thermomixer at 1,000 (motor speed) rpm for 3 min at 90°C. Residual cell debris was removed by centrifugation at 16,000 *g* for 20 min at −5°C, and 10 µl of the supernatant was taken for the subsequent determination of ATP levels using the ATP Determination kit (Invitrogen, Cat. No. A22066). Luminescence was assessed with a microplate reader (GlowMax, PROMEGA, delay time 2 s, integration time 10 s, and detection range 350–650 nm). Data were normalized to the number of cells, as determined by CASY Cell Counter Technology (Schaerfe System, Roche). At least four different clones were measured per strain and construct, each with two technical replicates that were pooled before statistical analysis. This experiment was repeated at least three times independently.

### Immunoblotting

For immunoblotting, 1–2 × 10^8^ cells were harvested (1,500 *g*, 5 min) after 16 h of plasmid‐based protein expression unless stated otherwise. Cell extracts were either produced using NaOH/2‐mercaptoethanol‐based chemical lysis followed by trichloroacetic acid (TCA)‐based protein precipitation (Riezman *et al*, 1983) as described previously (Kainz *et al*, 2017) or using 0.1 M NaOH. Afterwards, cells were incubated with 800 µl 0.1 M NaOH in a thermomixer at 1,400 rpm (motor speed) for 5 min at RT. After centrifugation (1,500 *g*, 5 min), the resulting cell pellets were resuspended in 150 µl 1× Laemmli buffer (4% SDS, 20% glycerol, 5% 2‐mercaptoethanol, 0.004% bromophenol blue, and 0.125 M Tris/HCl, pH approx. 6.8) and incubated at 1,400 rpm (motor speed) for 10 min at RT. Note that in order to preserve SDS‐stable oligomers that can alternatively be decomposed by heating at 95°C (Park *et al*, 2011a) typically used for protein denaturing, this step was performed at RT. Before loading the gel, the extracts were centrifuged again at 16,000 *g* for 1 min at RT and the supernatant was defined as whole‐cell extract (WCE). The extracts were separated on a 12% SDS polyacrylamide gel or 4–12% NuPAGE Bis–Tris Gels (Thermo Fisher). Blotting was performed using PVDF membrane (Carl Roth Gmbh & Co, Cat. No. T830.1) and CAPS transfer buffer (10 mM CAPS, pH 11, 10% methanol) or nitrocellulose blotting membrane (Life Science; Cat. No. 10600006) and Tris–Glycin blotting buffer (19 mM Tris, 188 mM glycine, and 20% methanol) for 2 h at 220 mA. After 1 h membrane blocking in 5% blocking solution (1× TBS and 5% skimmed milk powder), blots were probed with murine monoclonal antibodies against EGFP (Roche, Cat. No. 1814460, dilution: 1:5,000, in 1× TBS and 1% skimmed milk powder), amyloid beta (clone 6E10, BioLegend; Cat. No. #SIG‐39320, dilution: 1:750 in 1× TBS and 1% skimmed milk powder), DnaJA1 (LSBio, Cat. No. LS‐B8561/54406, clone name KA2A5.6, dilution 1:400 in 1× TBS‐Tween and 1% skimmed milk powder), GAPDH (Invitrogen, dilution 1:100,000 in 1× TBS and 1% skimmed milk powder), and rabbit polyclonal antibodies against yeast proteins (Sss1, Cyc1, Sec61, Pgk1, Tom22, Ssa1, Cox4 (Vögtle *et al*, 2017) and Ydj1 (the antibody against Ydj1 was generated by immunization of rabbits using the synthetic peptide SEENLKKLEEILPPRIC coupled to keyhole limpet hemocyanin via an N‐terminal cysteine), and the respective peroxidase‐conjugated affinity‐purified secondary antibodies (Sigma; Cat. No. A9044 and Cat. No. A0545 against murine or rabbit antibodies, respectively). For the membrane detection, Clarity^TM^ Western ECL Substrate (BioRad, Cat. No. 170‐5061) and ChemiDoc^TM^ Touch Imaging System (BioRad) were utilized. Data were analyzed and quantified using the densitometry tools of ImageLab (5.2) software (Bio‐Rad laboratories) after automatically obtaining optimal exposure times. To verify linear relation between signal intensity and protein amount during quantification of the relative tetramer/monomer ratio, whole cell extract (WCE) was loaded onto SDS–PAGE at four appropriate dilutions per sample (using separate gels for quantification of tetramer and monomer).

### Immunoprecipitation in yeast

For immunoprecipitation, ~10^9^ cells were harvested 16 h after induction of plasmid‐based protein expression and resuspended in 200 µl lysis buffer P+ (50 mM Tris/HCl pH 7.4, 150 mM NaCl, 1% Triton X‐100, 1 mM EDTA, 1 mM PMSF, and 1× Roche Complete^®^ protease inhibitor cocktail). Acid‐washed glass beads (200 µl), pre‐chilled on ice, were added to cells, which were then disrupted in a bead disruptor (Mini‐BeadBeater 96, BioSpec Products, Inc., Bartlesville) for 4× 30 s at 2,000 rpm (motor speed) with 30 s cooling on ice in between. After centrifugation at 10,000 *g* for 10 min at 4°C, protein concentration of supernatant was determined using the Bradford assay (BioRad). Immunoprecipitation of FLAG‐tagged Ydj1 and DnaJA1 using Anti‐FLAG M2 Affinity gel (Sigma‐Aldrich, Cat. No. A2220) was performed according to the manufacturer’s protocol. Beads were washed twice with 500 µl 1× TBS (10 mM Tris/HCl, pH 7.6, 150 mM NaCl) and mixed with 2 mg of native protein extracts. The resin–protein suspension was incubated at 4°C under light shaking for 2 h after which the initial supernatant was removed and further processed. Beads were washed in 1× TBS 5–7 times and the associated proteins were eluted by incubating the resin in 50 µl 1x Laemmli buffer at 1,400 rpm (motor speed) for 10 min at RT. Final washing step and the initial supernatant were incubated with 30 µl StrataClean™ Resin to capture proteins (Agilent Technologies) with shaking at 1,400 rpm (motor speed) for 20 min at RT. Resin protein complexes were then centrifuged at 16,000 *g* for 1 min at RT. The supernatant was discarded and the resins (pellets) were incubated in 65 µl 1× Laemmli with shaking at 1,400 rpm (motor speed) for 10 min at RT. Before gel loading, samples were centrifuged at 16,000 *g* for 1 min at RT.

### EGFP‐A42 degradation kinetics

In order to assess the degradation kinetics of EGFP‐A42 in yeast strains, the translation inhibitor cycloheximide (final concentration 10 µg/ml) was added to yeast cultures 16 h after inducing EGFP‐A42 expression, and EGFP levels were assessed using flow cytometry directly before (t_0_) and 2 h (t_2_) after cycloheximide addition. For quantifications using flow cytometry (BD LSRFortessa), 30,000 cells were evaluated and analyzed with BD FACSDiva software (EGFP detection, 530/30 nm, excited by 488 nm laser). Data represent mean EGFP fluorescence of cells from t_2_ normalized to t_0_.

### Staining procedures and confocal microscopy analysis

MitoTracker™ Red CMXRos (Invitrogen, Inc.) was added directly to 1 ml of a stationary phase cell culture 16 h after EGFP‐A42 expression to a final concentration of 150 nM. Imaging was performed using a Leica SP5 confocal microscope with spectral detection (Leica, Inc.) and a 63x HCX PL APO NA 1.4 oil immersion objective. EGFP was excited at 488 nm and emission detected between 500 and 550 nm. MitoTracker Red was excited at 561 nm and emission detected between 575 and 700 nm. Fluorescence signals were acquired using noiseless hybrid photon detectors. Fluorescence and transmission images were recorded simultaneously and in a blinded fashion.

### Cell fractionation

For preparation of whole‐cell, mitochondrial, microsomal, and cytosolic fractions, ~10^9^ cells were harvested (1,500 *g*, 5 min) after 18 h of plasmid‐based protein expression and washed in double‐distilled H_2_O. Cells were then incubated in 2 ml DTT reduction buffer (100 mM Tris/H2SO4 pH 9.4, 10 mM DTT) with shaking at 1,000 rpm (motor speed) for 10 min at 30°C followed by centrifugation at 1,500 *g*, 5 min. Spheroplasts were generated by incubation of cells with zymolyase buffer (1.2 M sorbitol, 20 mM potassium phosphate buffer, pH 7.4) supplemented with 3 mg/mg (wet weight cell pellet) Zymolyase^®^‐20T (amsbio, Cat. No. 120491‐1) at 1,000 rpm for 45 min at 30°C. Spheroplasts were washed (1,500 *g*, 5 min) with 1.2 M sorbitol and homogenized in homogenization buffer (0.6 M sorbitol, 10 mM Tris/HCl pH 6.4, 1 mM EDTA, 1 mM PMSF, and 1x Complete^®^ protease inhibitor cocktail (Roche, Cat. No. 11697498001) with a cooled glass–Teflon potter on ice (20 strokes). After homogenization and removal of the nuclei and cell debris (1,500 *g*, 5 min, 4°C), we gained the cytoplasmic, post‐nuclear supernatant (PNS) containing the cytosol and other organelles. Part of the PNS was further centrifuged at 13,000 *g* for 15 min at 4°C to obtain mitochondrial fraction in the pellet, and microsomes and cytosol in the supernatant. The pellet was homogenized in 500 µl SEM buffer (250 mM sucrose, 1 mM EDTA, 10 mM MOPS/KOH pH 7.2) using glass–Teflon potter on ice (15 strokes) and was subjected to centrifugation at 13,000 *g* for 10 min at 4°C. The mitochondrial fraction was incubated in 200 µl 1x Laemmli buffer with continuous shaking at 1,400 rpm (motor speed) for 10 min at 25°C. In order to separate the microsomes from the cytosol, the supernatant was centrifuged at 100,000 *g* for 1 h at 4°C. The resulting pellet, mainly containing microsomes, was incubated with 200 µl 1× Laemmli buffer as described above. Supernatants of cytosolic and PNS fractions were further incubated with 60 µl StrataClean™ Resin to capture proteins (Agilent Technologies) with continuous shaking at 1,400 rpm (motor speed) for 20 min at RT. After incubation, samples were centrifuged at 16,000 *g* for 1 min at RT. Pellets of PNS and cytosol fraction were incubated with 200 µl 1× Laemmli buffer as described above. Before gel loading all fractions were centrifuged at 16,000 *g* for 1 min at RT.

### Mitochondrial proteomics

To investigate the mitochondrial‐ and mitochondria‐associated proteome, wild‐type cells expressing EGFP or EGFP‐A42 were grown in media containing “light” or “heavy” arginine (L‐arginine‐^13^C_6_,^15^N_4_ hydrochloride, 100 mg/l (Sigma‐Aldrich)) and lysine (L‐lysine ^13^C_6_, ^15^N_2_ hydrochloride, 100 mg/l (Sigma‐Aldrich)) stable isotopes, respectively. Equal amounts (based on OD_600_ measurements) of light‐ and heavy‐labeled cells were pooled, followed by isolation of mitochondria using cell fractionation as described above. After homogenization with 20 strokes in 2 ml homogenization buffer (0.6 M sorbitol, 10 mM Tris/HCl pH 6.4, 1 mM EDTA, 1 mM PMSF, 1× Complete^®^ protease inhibitor cocktail (Roche, Cat. No. 11697498001)), remaining cell debris and non‐broken cells were removed in two consecutive centrifugation steps (1,500 *g*, 4°C, 5 min). Mitochondria were further isolated by centrifugation at 13,000 *g* at 4°C and homogenization in 1 ml SEM buffer (250 mM sucrose, 1 mM EDTA, 10 mM MOPS/KOH pH 7.2) with 15 strokes in a glass–Teflon potter. After removing remaining cell debris (1,500 *g*, 4°C, 5 min), mitochondria were isolated by final centrifugation step at 16,000 *g*, at 4°C for 10 min. Pellet was resuspended in 30 µl SEM buffer, followed by protein concentration determination by Bradford assay (BioRad). The mitochondrial fractions were aliquoted, snap frozen in liquid nitrogen, and stored at −80°C upon further processing for mass spectrometry (MS).

MS analyses were performed on LTQ Orbitrap XL mass spectrometers (Thermo Fisher Scientific, Bremen, Germany) coupled to an 1,200 nanoflow‐HPLCs (Agilent Technologies GmbH, Waldbronn, Germany) essentially as described (Dumit *et al*, 2014). HPLC‐column tips (fused silica, 75 μm id, New Objective, Woburn, MA, USA) were self‐packed with Reprosil‐Pur 120 ODS‐3 (Dr. Maisch, Ammerbuch, Germany). Samples were applied onto the column without pre‐column. A gradient of A [0.5% acetic acid (LGC Promochem, Wesel, Germany) in water (HPLC gradient grade, Mallinckrodt Baker B.V., Deventer, Netherlands)] and B [0.5% acetic acid in 80% ACN (LC‐MS grade, Wako, Germany) in water] with increasing organic proportion was used for sample separation (loading with 2% B; separation ramp: from 10 to 30% B within 80 min). The flow rate was 250 nl/min and for sample application 500 nl/min. Data‐dependent acquisition was performed and the mass spectrometer switched automatically between MS (max. of 1 × 10^6^ ions) and MS/MS. Each MS scan was followed by a maximum of five MS/MS scans in the LTQ using normalized collision energy of 35% and a target value of 5,000. Parent ions with a charge state from z = 1 and unassigned charge states were excluded for fragmentation. The mass range for MS was *m/z* = 370 to 2,000. The resolution was set to 60,000. Mass spectrometric parameters were as follows: spray voltage 2.3 kV; no sheath and auxiliary gas flow; and ion‐transfer tube temperature 125°C.

The MS raw data files were processed by MaxQuant software (version 1.3.05; (Cox & Mann, 2008)), which performs peak detection, SILAC pair detection, generates peak lists of mass error‐corrected peptides, and database searches. Significant outliers were determined by Perseus (Tyanova *et al*, 2016) using Significance A.

A full‐length yeast Uniprot database (version Jan. 2014) was employed. Carbamidomethylcysteine was set as fixed modification, and methionine oxidation and protein amino‐terminal acetylation were set as variable modifications. Three missed cleavages were allowed, enzyme specificity was trypsin/P+DP, and the MS/MS tolerance was set to 0.5 Da. Peptide lists were further used to identify and relatively quantify proteins using the following parameters: peptide and protein false discovery rates (FDR) were set to 0.01, minimum peptide length was set to 7, minimum ratio count was set to 2, and identified proteins were re‐quantified. The “match‐between‐run” option (2 min) was used.

The mass spectrometry proteomics data have been deposited to the ProteomeXchange Consortium via the PRIDE (Perez‐Riverol *et al*, 2019) partner repository with the dataset identifier PXD012612 (Project accession number).

### Inhibition of Hsp70/Hsp40 complex

116‐9e(4‐[1,1’‐Biphenyl]‐4‐yl‐3,4‐dihydro‐6‐methyl‐2‐oxo‐5‐[(phenylmethoxy)carbonyl]‐1(2H)‐pyrimidine‐hexanoic acid) was obtained from Sigma (CAS Number 831217‐43‐7). 116‐9e is solved in DMSO according to the company’s manual. Cells were treated with final concentration of 100 µM 116‐9e and the control DMSO (1%) simultaneously with the start of Abeta expression when switching cells to galactose‐containing medium.

### *Drosophila* husbandry and genetics

Standard laboratory fly breeding was carried out at 25°C, 65–70% humidity, and a 12:12 h light:dark cycle. Fly food was prepared according to the Bloomington recipe as a semi‐defined cornmeal–molasses medium with slight modifications (per liter: 4.2 g agar–agar, 85.3 g sugar beet syrup, 7.5 g baker´s yeast, 8.3 g soymeal, 66.7 g cornmeal, 1.3 g p‐hydroxy‐benzoic acid methyl ester dissolved in 4.2 ml ethanol, and 5.25 ml propionic acid). All strains were isogenized with *w^1118^* flies, an isogenic line, for six generations. nSyb‐*GAL4* enhancer trap lines (y[1] w[*]; P{w[+m*]=nSyb‐GAL4.S}3) were used to drive expression (Bloomington stock number 51635). UAS‐Abeta42 flies were kindly provided by Dr. Koichi Iijima (Thomas Jefferson University, USA). The *Droj2* knockdown flies (y[1] w[*]; Mi{y[+mDint2]=MIC}Droj2[MI08491]/TM3, Sb[1] Ser[1]), carrying a disruption of one *Droj2* allele due to MiMIC insertion, were obtained by Bloomington Stock Center Indiana (Bloomington stock number 44980). For spatially and temporally controlled expression of Abeta42, male flies harboring the UAS‐Abeta42 construct were crossed with *GAL4* driver line female virgins in a ratio of 1:5–1:4. As controls (ctrl), male flies without UAS‐Abeta42 were also crossed with *GAL4* driver line female virgins. Parental flies, which were reared at standardized larval density, were transferred to new vials every 3^rd^ day and only 1 to 3‐day‐old progenitor flies from the F1 generation were used for experiments. The flies were anesthetized on a porous pad by CO_2_ application for max. 5 min, after which 20–30 female flies were transferred to small fly culture vials containing 2–3 ml food. After incubation at 29°C for 24 h, flies were used for further experiments.

### Whole‐mount brain immunostaining, confocal, and time‐gated STED (gSTED) imaging in *Drosophila*

For confocal microscopy, brains from 10‐ or 18‐day‐old adult female and male flies were dissected in cold HL3 solution and fixed in PBS containing 4% paraformaldehyde (w/v) for 40 min at RT on the shaker. For gSTED, 15‐day‐old male flies were used. After fixation, brains were washed three times for 20 min each with 2% PBT (PBS containing 2% Triton X‐100, vol/vol) for confocal microscopy, or with 1% PBT for gSTED. Brains were treated with 70% formic acid (Sigma) for 10 min at RT on the shaker, then washed in 2% PBT (confocal) or 1% PBT (gSTED) at RT for two times for 5 min each. Brains were blocked with 10% normal goat serum in 2% PBT (confocal) or 1% PBT (gSTED) for 2 h at RT. Then, brains were stained with primary antibodies in 2% PBT (confocal) or 1% PBT (gSTED) with 5% normal goat serum for 48 h at 4°C. After primary antibody incubation, brains were washed in 2% PBT (confocal) or 1% PBT (gSTED) for six times for 20 min each at RT.

Then, brains were incubated in 2% PBT (confocal) or 1% PBT (gSTED) with 5% normal goat serum containing biotin‐XX goat anti‐mouse IgG (H + L) (Thermo Fisher; 1:200) for 2 h at RT, for confocal detection or appropriate IgG subtype‐specific secondary antibodies for gSTED. Brains were washed in 0.7% PBT (confocal) or 1% PBT (gSTED) for six times for 20 min each, followed by incubation in 2% PBT with 5% normal goat serum containing streptavidin‐Alexa 594 conjugate (Biolegend, 1:100, for confocal detection only). For confocal detection, brains were further washed six times in 2% PBT for 20 min each. Afterwards brains were mounted in Vectashield containing DAPI (Vector abs; confocal detection). For gSTED, brains were stained with DAPI (0.1 µg/ml in PBS; D1306 Thermo Fisher Scientific) for 5 min at RT and finally mounted in ProLong Gold Antifade Mountant (Thermo Fischer Scientific, P36934) with high‐precision 1.5H coverslips (Carl Roth).

The following primary antibodies were used: mouse monoclonal IgG1 κ anti‐Aß antibody (clone 6E10; Biolegend, 803001; 1:200 for confocal, 1:400 for gSTED), mouse IgG2b anti‐ATP5A (clone 15H4C4, Abcam, ab14748, 1:100 for gSTED), and mouse IgG2a anti‐KDEL (clone 10C3, Enzo Lifesciences, ADI‐SPA‐827, 1:100 for gSTED). These primary antibodies were detected with the following fluorophore‐conjugated secondary antibodies for gSTED microscopy: STAR RED FluoTag‐X2 sdAb anti‐Mouse IgG1 (Clone 10A4, Nanotag, N2002‐AbRED‐S, 1:500), Alexa Fluor 594 Goat anti‐Mouse IgG2b (Invitrogen, A‐21145, 1:100), or Alexa Fluor 594 Goat anti‐Mouse IgG2a (Invitrogen, A‐21135, 1:100). Cross reactivity between secondary antibodies against specific mouse IgG subtypes was tested beforehand. No unspecific staining was observed when swapping the secondary antibodies (Appendix Fig S2E).

Image stacks of specimens were acquired on Leica TCS SP8 confocal microscope (Leica Microsystems) using 40×, 1.3 NA oil objective for whole‐brain imaging. For visualization, the confocal images were exported as TIF and processed by linear adjustment of brightness by open‐source software Fiji ImageJ 1.52p. For Abeta intensity quantification, the average intensity Z‐projection was performed with the same number of stacks for each scanning and the mean grey value of the central brain was measured. Total area (representing areas of Abeta aggregates) was calculated following image thresholding (the same image thresholding was applied across all experiments) using the plugin “Analyze/Analyze particles.” Each experiment was done with six to nine replicates and performed independently twice. For inter‐experiment comparison, all measurements were internally normalized versus internal Droj2^+/−^‐UAS‐A42 fly brains. Statistical significance was evaluated by unpaired, two‐tailed *t*‐test. 2D‐gSTED images were acquired with an Abberior Instruments Expert line STED setup (Abberior Instruments GmbH), equipped with an inverted IX83 microscope (Olympus), two pulsed STED lasers for depletion at 775 nm (0.98 ns pulse duration, up to 80 MHz repetition rate) and at 595 nm (0.52 ns pulse duration and 40 MHz repetition rate), and pulsed excitation lasers (at 488, 561, and 640 nm). The system was operated by Imspector software (Abberior Instruments GmbH, version 16.3.13367). The dyes STAR RED and Alexa Fluor 594 were depleted with a pulsed STED laser at 775 nm. DAPI was imaged in confocal mode only, with a 405 nm excitation laser. Time gating was set at 750 ps for gSTED images. Fluorescence signals were detected sequentially by avalanche photodiode detectors at appropriate spectral regions. 2D confocal and gSTED Images were acquired with a 100×, 1.40 NA oil immersion objective with a pixel dwell time of 2 µs and 10× lines accumulation (confocal) or 30× lines accumulation (gSTED) at 16‐bit sampling, and a field of view of 10 × 10 µm. Lateral pixel size was set to 20 nm. Within each experiment, samples were acquired with equal settings. Raw dual‐channel gSTED images were processed for Richardson–Lucy deconvolution using the Imspector software (Abberior Instruments GmbH, version 16.3.13367). The point spread function was automatically computed with a 2D Lorentz function having a full‐width half‐maximum of 40 nm, based on measurements with 40 nm Crimson beads. Default deconvolution settings were applied. Deconvolved 8‐bit gSTED images were used for quantification.

Mitochondria morphometric parameters were quantified using the ImageJ (version 1.52p, NIH) function “Analyze particles” upon signal threshold of deconvolved ATP5A gSTED images. Pearson´s correlation coefficient (above threshold), Mander’s co‐localization coefficients tM1 (Abeta/KDEL) above auto‐threshold of channel 2, and tM2 (KDEL/Abeta) above auto‐threshold of channel 1 were used to determine the degree of co‐localization on 8‐bit confocal images using the ImageJ (version 1.52p, NIH) plugin “Coloc 2”, with the PSF set to 10 pixels, in a single ROI (50 × 50 pixels) for a given optical slice. For gSTED microscopy and analysis, experiments were performed blinded and repeated two times on different biological replicates.

### Quantitative real‐time PCR

Forty fly heads per each genotype were collected on dry ice and total RNA was extracted using TRIZOL (ThermoFisher Scientific) according to the manufacturer’s protocol. RNA concentration and quality were analyzed spectrometrically. Reverse transcription of RNA was performed with 2–4 µg RNA using QuantiNova^TM^ Reverse Transcription kit (Qiagen) according to the manual. Sample dilution for quantitative PCR was determined by standard curves using dilution series of sample mixes using the Applied Biosystems StepOnePlus^®^ (Thermo Fisher) with Biozym Blue S'Green qPCR Kit Separate ROX (Biozym) or QuantiNova PCR Kits (Qiagen). PCR efficiencies were within 85–130% for all PCR reactions. The efficiencies of the primers were included in the calculation (Nolan *et al*, 2006). Target mRNA quantification was calculated by relative ΔCt comparison (ΔΔCt‐method) using *Rpl32* and *αTubulin* mRNA as internal standards (revealing similar results) and subsequently normalized to signals from wild‐type fly heads. Primers used for the RT–PCR are listed in Appendix Table S2.

### *Drosophila* protein extracts and immunoblotting

Thirty fly heads per genotype were collected on dry ice and squished in 30 µl RIPA buffer (50 mM Tris/HCl pH 8.0, 0.5% sodium deoxycholate, 1% Triton X‐100, 150 mM NaCl, and 1% SDS) using a plastic pistil and incubated for 15 min at RT. Samples were centrifuged at 16,000 *g* for 1 h at 4°C and the supernatant was used as soluble (protein) fraction. The pellet (insoluble fraction) was resuspended in 30 µl 70% formic acid (Sigma‐Aldrich) using a plastic pistil, followed by centrifugation at 16,000 *g* for 20 min at 4°C. Formic acid was evaporated using a Speed Vac and the residual pellet was resuspended in 18 µl DMSO (Sigma‐Aldrich). After addition of 5× Laemmli buffer, samples were incubated with continuous shaking at 1,400 rpm (motor speed) for 5 min at 95°C. Before gel loading, samples were centrifuged at 16,000 *g* for 20 min at RT. Insoluble fraction was used for Abeta42 detection, whereas soluble fraction for the detection of soluble proteins. Insoluble fraction was loaded to 12% NuPage (Thermo Fisher) gels. Electrophoresis was performed using MES buffer (ThermoFisher). Blotting was carried out using Tris–glycine buffer (25 mM Tris, 0.5 M glycine, and 20% methanol) and nitrocellulose membrane (GE Healthcare Protran BA83) for 3 h, 50 mA at 4°C. Blots were probed with murine monoclonal antibodies against amyloid beta (clone 6E10, BioLegend; Cat. No. #SIG‐39320, dilution: 1:750) and corresponding secondary antibody. Soluble fraction was loaded to 4–12% NuPage (Thermo Fisher) gels with subsequent electrophoresis using MOPS buffer (ThermoFisher). Blotting was carried out using CAPS transfer buffer (10 mM CAPS, pH 11, 10% methanol) and PVDF membrane (Carl Roth GmbH & Co. KG) for 1.5 h, 220 mA at 4°C. Blots were probed with murine monoclonal antibodies against DnaJA1 antibody (LsBio, Cat. No. LS‐C87957, dilution: 1:4,000) and corresponding secondary antibody.

### Immunoprecipitation of *Drosophila* proteins

In order to assess the interaction between fly Droj2 and synthetic A42, 130 fresh heads of *w^1118^* flies were collected on dry ice and homogenized using a plastic pistil in MSD lysis buffer with 1 µl per head (150 mM NaCl, 20 mM Tris pH 7.5, 1 mM EGTA, 1 mM EDTA, 1% Triton X‐100, 2× Complete protease inhibitor cocktail (Roche), 1× Phosstop (Roche)). Homogenates were centrifuged for 1 h at 16,000 *g* and at 8°C. Direct immunoprecipitation was performed using 50 µl PureProteome™ Protein G Magnetic Bead System (Millipore) according to the manufacturer’s instructions, but with 2 h incubation (instead of 20 min) for antibody coupling to the beads. Briefly, the supernatant after protein extraction prior co‐immunoprecipitation was pre‐cleared applying magnetic beads (without antibody) by incubation on a rotating wheel for 20 min at 4°C. Samples were further centrifuged for 10 min at 4°C and 16,000 *g*. Pre‐cleared protein extracts were incubated for 4 h at 4°C with beads coupled to the amyloid beta‐specific antibody (clone 6E10) (5 µl) (BioLegend; Cat. No. #SIG‐39320) and with 500 µM Abeta42 peptides (1‐42, solution, Bachem). After bead separation, the supernatant was recovered and used as a control for non‐bound proteins, whereas three washing steps were performed to remove excess proteins. Elution was performed by incubation with 20 µl 2× SDS sample buffer (100 mM Tris–HCl, pH 6.8, 4% SDS, 20% glycerol, 5% 2‐mercaptoethanol, 2 mM EDTA, and 0.1 mg/ ml bromophenol blue) at 95°C for 5 min and subsequent incubation for 5 min at RT with continuous shaking at 1,400 rpm (motor speed). To capture the proteins from supernatant and washing step fraction, the samples were incubated with 30 µl StrataClean™ Resin (Agilent Technologies) for 20 min at RT and continuous shaking at 1,400 rpm. Subsequently, supernatant and washing controls were centrifuged for 1 min at 16,000 *g* and RT and the resin beads were incubated in 1× Laemmli buffer for 10 min and shaking at 1,400 rpm (motor speed) and RT. Prior to loading the gel, samples were centrifuged at 16,000 *g* for 1 min.

### *Drosophila* survival assays

To determine survival upon challenge under manganese stress, 1‐ to 3‐day‐old female flies (both sexes, kept separately) were incubated at 29°C for 24 h for the Abeta42 expression and transferred into fresh vials with filter papers soaked with solution containing 10% sucrose and 20 mM MnCl_2_ as previously described (Büttner *et al*, 2013). Filters were kept wet at all times and numbers of dead flies were recorded at indicated time points. For realization of manganese stress experiments, flies were crossed on defined sugar/yeast/agar (SYA) food (per liter: 10 g agar–agar, 50 g sucrose, 100 g baker´s yeast, and 3 g p‐hydroxy‐benzoic acid methyl ester known as nipagin dissolved in 30 ml ethanol and 3 ml propionic acid). Each experiment was performed with 110–130 flies per genotype and repeated at least three times in independent experiments with flies derived from independent crosses.

### Memory performance in *Drosophila*

Olfactory short‐term memory (STM) was performed as described previously (Gupta *et al*, 2013). In brief, 50–100 18‐day‐old flies (corresponding to one technical replicate) were split into two portions, transferred to empty plastic vials, and allowed to acclimatize in the dark for 30 min. Training and memory trials were performed in a climatized chamber (25°C 65–70% humidity) under dim red light. Each portion of flies was tapped into a standard T‐maze (Tully & Quinn, 1985) and allowed to rest for 2 min. Air flow was set to 2 l/min per maze. Flies were exposed to a conditional olfactory stimulus, either 4‐methylcyclohexanol (mixture of *cis* and *trans*, Sigma‐Aldrich #153095, 1:55 in paraffin oil) or 3‐octanol (Sigma‐Aldrich # 218405, 1:150 in paraffin oil) for 1 min, paired with mild electric shocks (12 × 1.25 s pulses of 60V with 3.75 s intervals in‐between), and followed by a flush with air for 30 s. Then, flies were exposed to the other odor without electric shock for 1 min, followed by a 2 min flush with air. The other portion of flies were trained with the odors switched. Flies were introduced to the choice point of the T‐maze and allowed to choose between the conditional, shock‐paired stimulus (CS^+^) and the non‐shocked control stimulus (CS^‐^) for 2 min. The performance index was determined by averaging the performance of each maze (with either 4‐methylcyclohexanol or 3‐octanol as the CS^+^), which was calculated by subtracting the number of male flies avoiding the CS^‐^ from the number of male flies avoiding the CS^+^, divided by the total number of male flies. In total, six experiments with 3–4 technical replicates each were performed to assess STM performance. The memory experiment was performed in a blinded fashion.

Of note, memory performance usually is determined by means of olfactory aversive conditioning, which requires flies to be raised on standard cornmeal yeast molasses food, containing low concentrations of yeast (0.75%) (Gupta *et al*, 2013; Malik & Hodge, 2014), whereas our experiments have been performed using high‐level yeast food (10% yeast). To overcome this problem, we switched to a low yeast fly food condition for the memory experiments.

### *In vitro* aggregation assay with Abeta42 and DnaJ

In order to prepare homogenous monomer solutions, 1 mg of Abeta42 (Bachem) was carefully dissolved in 111 μl of 1,1,1,3,3,3‐hexafluoro‐2‐propanol (HFIP, ≥ 99%, Sigma‐Aldrich) for 1 h with occasional vortexing. The HFIP‐Abeta42 solution was then sonicated for 10 min and subsequently centrifuged at 20,000 *g* at 4°C for 30 min, after which the top 80% of the total volume was removed and divided into 5.54 μl aliquots. Aliquots were kept for 2 h under a fume hood to evaporate HFIP. The dried Abeta42 aliquots were stored at −20°C.

Thioflavin T (ThT, Sigma‐Aldrich) was freshly prepared before each experiment by dissolving 3 mg of ThT in 1 ml of HPLC‐grade water (Roth). The solution was filtered using a 0.2 μm filter (VWR) and the final ThT concentration was calculated from its absorbance in water at 412 nm measured by an Implen Nanophotometer, using a molar extinction coefficient of 36,000/M/cm.

Recombinant DnaJ was purchased from Genway Biotech (1 mg/ml in 25 mM Tris–HCl, pH 7.5, 100 mM NaCl, 5 mM 1,4‐Dithiothreitol, and 10% glycerol) and diluted 1:10 in PBS (137 mM NaCl, 2.7 mM KCl, 10 mM Na2HPO4, pH 7.4, VWR). The resulting solution was divided into 111 μl aliquots and stored at −20°C.

Right before the experiment, an Abeta42 aliquot was dissolved in 5.54 μl of dimethyl sulfoxide (DMSO, ≥ 99.7%, Sigma‐Aldrich) and kept at RT for 10 min. 0.5 μl of the ThT stock solution was mixed with 25.5 μl of PBS and a 111 μl DnaJ aliquot. Finally, 1.38 μl of DMSO‐Abeta42 solution was added, resulting in final concentrations 20 μM ThT, 2 µM DnaJ, and 20 µM Abeta42. After 15 s shaking, the solution was pipetted into the well of the microplate.

For the Abeta42 + BSA samples, the 111 µl DnaJ aliquot was replaced by 2.5 µM BSA (Sigma‐Aldrich) dissolved in an equivalent buffer consisting of 90% PBS and 10% Tris–HCl buffer (25 mM Trizma base (≥ 99.9%, Sigma‐Aldrich)), pH adjusted to 7.5 using 0.1 M HCl (Sigma‐Aldrich), with additional 100 mM NaCl (Sigma‐Aldrich), 5 mM 1,4‐Dithiothreitol (VWR), and 10% glycerol (VWR).

To analyze the kinetics of amyloid aggregation, ThT fluorimetry was conducted at 37°C for 6 h using a TriStar2 S LB 942 microplate reader (Berthold Technology) with shaking between measurements. ThT was excited at 440 nm and fluorescence emission monitored at 495 nm.

### *In vitro* aggregation assay with Abeta42 and DnaJA1

One milligram of Abeta42 (Bachem) was carefully dissolved in 444 μl of 1,1,1,3,3,3‐hexafluoro‐2‐propanol (HFIP, ≥ 99%, Sigma‐Aldrich) for 1 h with occasional vortexing. The HFIP‐Abeta42 solution then was centrifuged at 21,500 *g* at 4°C for 30 min, after which the top 80% of the total volume was divided into 24 μl aliquots. Aliquots were again centrifuged at 6,708 *g* for 30 s and then kept overnight under a fume hood to evaporate HFIP. The Abeta42 aliquots were then re‐dissolved in 50 µl HFIP, centrifuged at 6,708 *g* for 30 s, and dried overnight under a fume hood again. The dried Abeta42 aliquots were stored at −20°C.

Recombinant DnaJA1 (0.5 mg/ml in 10 mM Tris–HCl, pH 7.5, 1 mM EDTA, and 20% glycerol) was purchased from Cusabio and stored at −20°C. Right before the experiment, two Abeta42 aliquots were each dissolved in 6 μl of dimethyl sulfoxide (DMSO, ≥ 99.7%, Sigma‐Aldrich) and kept at RT for 10 min. Then, 83.2 µl of the recombinant DnaJA1 solution was diluted in buffer containing 10 mM Tris–HCl, pH 7.5, 1 mM EDTA, and 20% glycerol, to reach a final volume of 396 µl. Four microliter of DMSO‐Abeta42 solution was added, resulting in concentrations of 20 μM Abeta42 and 2 µM DnaJA1. The aggregation properties of Abeta42 with and without DnaJA1 were assessed over time utilizing immunoblot assay.

### Animal strains and housing

Breeding pairs of triple transgenic mice (3xTg), carrying the PS1M146V, APPSwe, and tauP301L transgenes (Oddo *et al*, 2003), were initially purchased from The Jackson Laboratories (JAX), United States of America. Animals used in this study were bred in the animal facility of the Institute of Molecular Biosciences, Graz, Austria, using the breeding pairs from Charles River Laboratories. The control strain used for the AD disease model was B6129SF2/J, also bred in‐house with breeding pairs from Charles River Laboratories. Briefly, animals were housed in groups of two to four animals per cage under specific pathogen‐free (SPF) conditions in a 14 h/10 h light/dark cycle with *ad libitum* access to standard chow (Ssniff, cat. #V1536) and autoclaved tap water. Autoclaved nest material and one paper house per cage served as cage enrichment. All animal experiments were performed in accordance with national and European ethical regulation (Directive 2010/63/EU) and approved by the responsible institutional or government agencies (Bundesministerium für Wissenschaft, Forschung und Wirtschaft, BMWFW, Austria: BMWFW‐66.007/0032‐V/3b/2019).

### Immunoprecipitation of mouse brain homogenate

Mouse brain tissue was dissected after euthanizing the animal by cervical dislocation under isoflurane anesthesia. In order to assess the interaction between mouse DnaJA1 and Abeta (as well as Abeta‐protein sequence‐containing protein APP), one mouse brain was cut into small pieces and then homogenized in pre‐cooled 15 ml H buffer (225 mM mannitol, 75 mM sucrose, 10 mM MOPS, 1mM EGTA, and 0.5% BSA; (Walls *et al*, 2012)) additionally containing 1× Complete^®^ protease inhibitor cocktail (Roche, Cat. No. 11697498001) and 4% DEA with a cooled loose glass potter on ice (45 strokes). Two milliliter aliquot was taken and centrifuged for 15 min at 3,000 *g* and 4°C; the supernatant represents soluble whole‐cell extract (WCE). To capture the proteins from supernatant, the sample was incubated with 30 µl StrataClean™ Resin (Agilent Technologies) for 20 min at RT and continuous shaking at 1,400 rpm. Subsequently, supernatant was centrifuged for 1 min at 16,000 *g* and RT and the resin beads were incubated in 1x Laemmli buffer for 10 min and shaking at 1,400 rpm (motor speed) and RT. Prior to loading, the gel samples were centrifuged at 16,000 *g* for 1 min. To gain a cytosolic enriched fraction, the remaining 13 ml of the homogenate were centrifuged for 5 min at 1,000 *g*, at 4°C and the resulting supernatant—to remove the mitochondria—centrifuged again at 2,500 rpm for 10 min, at 4°C. The resulting supernatant was—to remove microsomes—centrifuged at 40,000 rpm for 1.5 h and 4°C. The cytosol‐enriched supernatant was concentrated using centrifugal filters (Millipre, Amicon Ultra‐15 Ultracel‐3K) at 4,000 *g* at 4°C until a reduction to 10% of the starting volume. This cytosol‐enriched brain tissue homogenate was further used for direct immunoprecipitation using 50 µl PureProteome™ Protein G Magnetic Bead System (Millipore) according to the manufacturer’s instructions, but with 2 h incubation (instead of 20 min) for antibody coupling to the beads.

Briefly, protein extract was incubated for 4 h at 4°C with beads coupled to the 5 µl Abeta‐specific antibody (6E10), DnaJA1‐specific antibody, or without antibody. After bead separation, the supernatant was recovered and used as a control for non‐bound proteins, whereas three washing steps were performed to remove excess proteins. Elution was performed by incubation with 20 µl 2× SDS sample buffer (100 mM Tris–HCl, pH 6.8, 4% SDS, 20% glycerol, 5% 2‐mercaptoethanol, 2 mM EDTA, and 0.1 mg/ ml bromophenol blue) at 95°C for 5 min and subsequent incubation for 5 min at RT with continuous shaking at 1,400 rpm (motor speed). Prior to loading the gel, samples were centrifuged at 16,000 *g* for 1 min.

### Human hippocampi protein extraction

Experiments with human materials were in accordance with the ethical committee at the University of Bayreuth (Germany). Informed consent was obtained from all subjects and the experiments conformed to the principles set out in the WMA Declaration of Helsinki and the Department of Health and Human Services Belmont Report. *Post mortem* tissues of hippocampi from AD patients and non‐demented controls were obtained from the Netherlands Brain Bank (Amsterdam, The Netherlands). Experiments were carried out in laboratories at the University of Bayreuth. Hippocampi of 5 AD and 4 non‐demented control patients were chopped in 200 mg pieces and homogenized with a tightfitting pre‐cooled glass pestle with RIPA buffer (150 mM sodium chloride, 1.0% (v/v) NP‐40, 0.5% (w/v) sodium deoxycholate, 0.1% (w/v) sodium dodecyl sulfate, 50 mM Tris–HCl (pH 7.4), 1x mini complete (Roche), 2 mM EDTA, 2 mM PMSF, 5 mM chloroacetamide, 20 mM sodium fluoride, and 1 mM sodium vanadate). To remove the debris, homogenized brain samples were centrifuged (15 min, 1,000 *g*, 4°C) and homogenized again. After the second centrifugation, the supernatants were collected and used for BCA protein concentration (ThermoFisher). Samples were stored at −80°C. For the western blotting, samples were heated with laemmli buffer (4% SDS, 20% glycerol, 0.004% bromophenol blue, 0.125 M Tris–HCl (pH 6.8), and 10% 2‐mercaptoethanol) at 99°C and loaded to 12.5% SDS gels (50 µg/lane). Gels were run at 6–12 mA and blotted with transfer buffer for 45 min at 300 V, 175 mA. Membranes were stained with Ponceau S stain, blocked for 1 h in 5% milk buffer, incubated over night with primary antibody (6E10: BioLegend, Cat. No. SIG‐39320; and DnaJA1: LsBio, Cat. No. LS‐C87957), and next day incubated 1 h with corresponding secondary antibody.

### Statistical analyses

Data are presented either as dot plots or line graphs showing mean ± SD. Analysis always involved all generated data points or samples. Sample sizes of yeast and fly experiments were chosen based on standard power analysis (statistical power: ≥ 0.8 and α value: < 0.05) or on previous published studies (Chen *et al*, 2020; Pooryasin *et al*, 2021). Human and mouse tissue sample analysis was explorative and conducted without pre‐specified effect size. Indicated sample size (see figure legends) always refers to biological replicates (independent of cultured cells or animal populations). If not otherwise stated, statistical testing was performed using OriginPro 2016 statistic software or GraphPad Prism™ 9 software. One‐sample or two‐sample Student’s *t*‐test (unpaired) and analysis of variance (ANOVA) with Tukey’s *post hoc* tests served to compare two or multiple groups, respectively. The reported significance values are always two sided. Normal distribution of data was confirmed using Shapiro–Wilk’s test or by visual inspection of QQ plots of residuals. Homogeneity of variance was tested using Levene’s test. Data violating these assumptions were transformed to meet the assumptions of linear models. Survival data of *D. melanogaster* experiments were statistically analyzed using log rank (Mantel Cox) test and in case of multiple comparisons, Bonferroni correction was applied (GraphPad Prism™ 9 software). The interaction between Abeta42 expression and other genetic backgrounds (e.g., *Droj2 mutation*) was evaluated using a Cox proportional hazards model (IBM SPSS statistics software—Version 25). Data from yeast experiments showing several time points were analyzed using a two‐way repeated‐measures ANOVA with the time factor set as the repeated variable that was Greenhouse–Geisser‐corrected in case of sphericity violation (using GraphPad Prism Software, *Version 9*), followed by testing simple main effects (i.e., multiple comparisons of different levels of each factor that were Tukey‐corrected if the factor had more than two levels) in case of interaction significance. In case of missing values (due to technical reasons) the mixed effects model approach was used as recommended by Prism software.

## Author contributions

**Frank Madeo:** Conceptualization; Supervision; Funding acquisition; Writing – review & editing. **Tobias Eisenberg:** Conceptualization; Supervision; Funding acquisition; Writing – review & editing. **Julia Ring:** Conceptualization; Data curation; Formal analysis; Investigation; Methodology; Writing – original draft. **Jelena Tadic:** Conceptualization; Data curation; Formal analysis; Investigation; Methodology; Writing – original draft. **Selena Ristic:** Investigation. **Michael Poglitsch:** Investigation. **Martina Bergmann:** Investigation. **Dirk Mossmann:** Investigation; Methodology. **Roozbeh Hajiraissi:** Investigation. **Marcel Hanke:** Investigation. **YongTian Liang:** Investigation. **Victoria Küttner:** Software; Investigation. **Heimo Wolinski:** Investigation. **Andreas Zimmermann:** Writing – review & editing. **Filomena Broeskamp:** Investigation; Methodology. **Julia Westermayer:** Investigation. **Claudia Abraham:** Investigation. **Simon Schauer:** Investigation. **Lana Domuz Trifunović:** Investigation. **Christopher Dammbrueck:** Investigation. **Sebastian J Hofer:** Investigation. **Mahmoud Abdellatif:** Software; Formal analysis; Methodology. **Guido Grundmeier:** Writing – review & editing. **Guido Kroemer:** Writing – review & editing. **Ralf J Braun:** Resources; Methodology; Writing – review & editing. **Cornelia Sommer:** Investigation. **Mirjana Ninkovic:** Investigation. **Sandra Seba:** Investigation. **Patrick Rockenfeller:** Investigation. **F‐Nora Voegtle:** Supervision; Methodology. **Jörn Dengjel:** Formal analysis; Supervision; Validation; Investigation. **Chris Meisinger:** Supervision; Methodology. **Stephan J Sigrist:** Supervision; Methodology. **Adrian Keller:** Supervision; Methodology. **Nemanja Radic:** Investigation. **Marta Maglione:** Investigation; Methodology; Writing – review & editing. **Andrea Jerkovic:** Investigation. **Leonie Mikolasch:** Investigation. **Daiana Nerina Moretti:** Investigation. **Niklas Hansen:** Investigation.

## Disclosure and competing interests statement

GK has been holding research contracts with Daiichi Sankyo, Eleor, Kaleido, Lytix Pharma, PharmaMar, Samsara, Sanofi, Sotio, Vascage, and Vasculox/Tioma. GK is on the Board of Directors of the Bristol Myers Squibb Foundation France. GK is a scientific co‐founder of everImmune, Samsara Therapeutics, and Therafast Bio. GK is the inventor of patents covering therapeutic targeting of aging, cancer, cystic fibrosis, and metabolic disorders. The other authors declare that they have no conflict of interest.

## Supporting information



Appendix

Expanded View Figures PDF

Source Data for Expanded View and Appendix

Source Data for Figure 1

Source Data for Figure 2

Source Data for Figure 3

Source Data for Figure 4

Source Data for Figure 5

Source Data for Figure 6

## Data Availability

The mass spectrometry proteomics data have been deposited to the ProteomeXchange Consortium via the PRIDE (Perez‐Riverol *et al*, 2019) partner repository with the dataset identifier PXD012612 (Project accession number) http://www.ebi.ac.uk/pride/archive/projects/PXD012612.
