# Supplementary material for: The HSP40 chaperone Ydj1 drives amyloid beta 42 toxicity
Source: EMBO Mol Med. 2022 Apr 4;14(5):e13952. doi: 10.15252/emmm.202113952 (PMC9081910; doi:10.15252/emmm.202113952)
Supplement: Supplementary file 1 — Appendix [file EMMM-14-e13952-s005.pdf]

Appendix Supplementary Figures and Tables

Appendix Figure S1 and legends……………………………………………….……………………………2

Appendix Figure S2 and legends…………………………………………………………………….…………4

Appendix Table S1: Primers used

for cloning of pESC-his-EGFP and pESC-ura-FLAG constructs……………………………………….……….6

Appendix Table S2: Primers used for quantitative

reverse transcription real-time PCR (RT-qPCR) ……………………………………………………..….……….6

Appendix Table S3: Hits from proteomic approach………………………………………………………………6

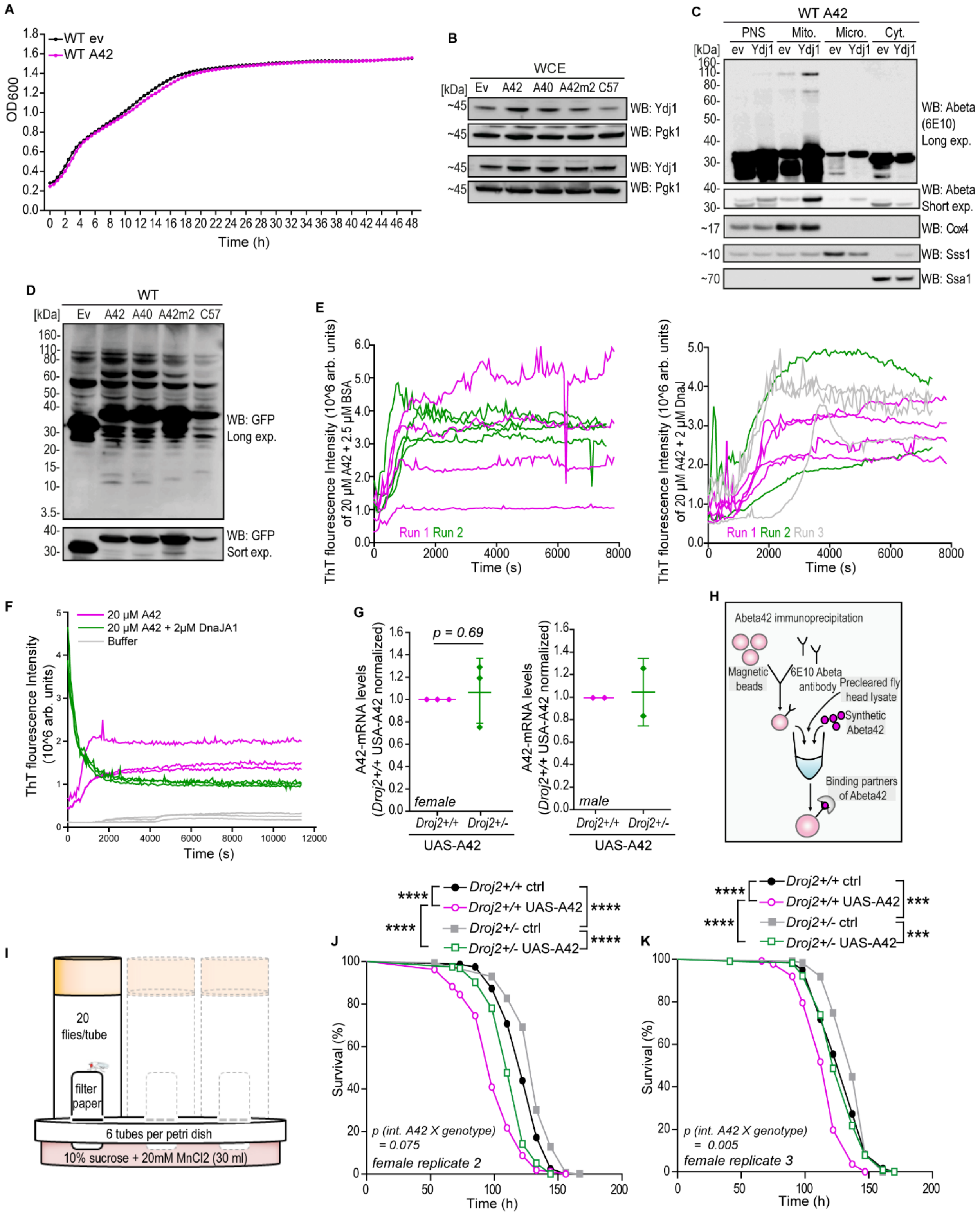

Appendix Figure S1

(**A**) Growth curve of wild type (WT) yeast cells expressing of EGFP-A42 or the corresponding empty vector control (ev) expressing EGFP only. Monitoring OD_{600} using Bioscreen C MBR with BioScreener™ software over 48 h after shift to galactose containing media to start expression of EGFP-constructs.

(**B**) Immunoblots of whole cell extract (WCE) of wild type yeast cells after 16 h of expression of EGFP-A42. Pgk1 was used as a loading control. See also Fig EV1F-G

(**C**) Immunoblot of total cytoplasmic post-nuclear supernatant (PNS), mitochondrial (Mito.), microsomal (Micro.) and cytosolic (Cyt.) fractions of wild type (WT) cells after 16 h of expression of EGFP-A42 (A42) and co-overexpressing Ydj1-FLAG using Abeta-specific antibody (Abeta) 6E10 with long (Long exp.) and short time exposure (Short exp.). Cox4-specific antibody is a marker for mitochondria, Sss1 for microsomes and Ssa1 was used to verify cytosolic fraction.

(**D**) Dot blot graph of 30,000 cells of FACS analysis of wild type cells after 42 h of expressing EGFP stained with DHE. FITC-A and PerCP-Cy5-5-A for green and red fluorescence, respectively. Gates to gain % of EGFP or Eth positive cells have been set with non EGFP containing or not DHE stained cells, respectively; and compensation has been applied using appropriate single fluorescence controls.

(**E**) Single measurements of A42 beta-sheet rich assembly formation with BSA or with DnaJ monitored by increase of ThT fluorescence over time. Means are represented in Fig 4H. Different colors represent independent runs.

(**F**) A42 beta-sheet rich assembly formation monitored by increase of ThT fluorescence over time with or without DnaJA1. Data represent means of at least 8 measurements.

(**G**) qPCR analysis of Abeta42-mRNA levels of 3-6 days old Droj2 knockdown flies (*Droj2+/-*) expressing human Abeta42 (UAS-A42) normalized to corresponding isogenic w^{1118} wild type flies (*Droj2+/+*) expressing human Abeta42 (UAS-A42). Reference gene is α-Tubulin and Rpl32. Dot plots show all data points along with the mean (line) ± s.d.. ns, $p > 0.05$. One sample t-test against 1.

(**H**) Schematic illustration of immunoprecipitation (IP: 6E10) of w^{1118} wild type flies (Droj2+/+) using synthetic Abeta42. Data shown in Figure 6G.

(**I**) Schematic illustration of fly manganese treatment.

(**J-K**) 2 replicate experiments of Fig 7A. Survival of female w^{1118} wild type flies (*Droj2+/+*) and Droj2 knockdown flies (*Droj2+/-*) with expression of human Abeta42 (UAS-A42) or without, control (ctrl), upon supplementation of food (10% sucrose) with 20 mM $MnCl_2$. Survival has been determined at indicated time points. n = 6 with 100-120 flies per experiment. The indicated p value refers to the interaction (int.) term of a Cox Proportional Hazards model comparing Abeta42 toxicity (UAS-A42 vs. ctrl) and Droj2 expression (*Droj2+/+ vs. Droj+/-*) as main factors. The following pairwise comparisons of the indicated groups survival were done by Log Rank test (****, $p < 0.0001$; ***, $p < 0.001$).

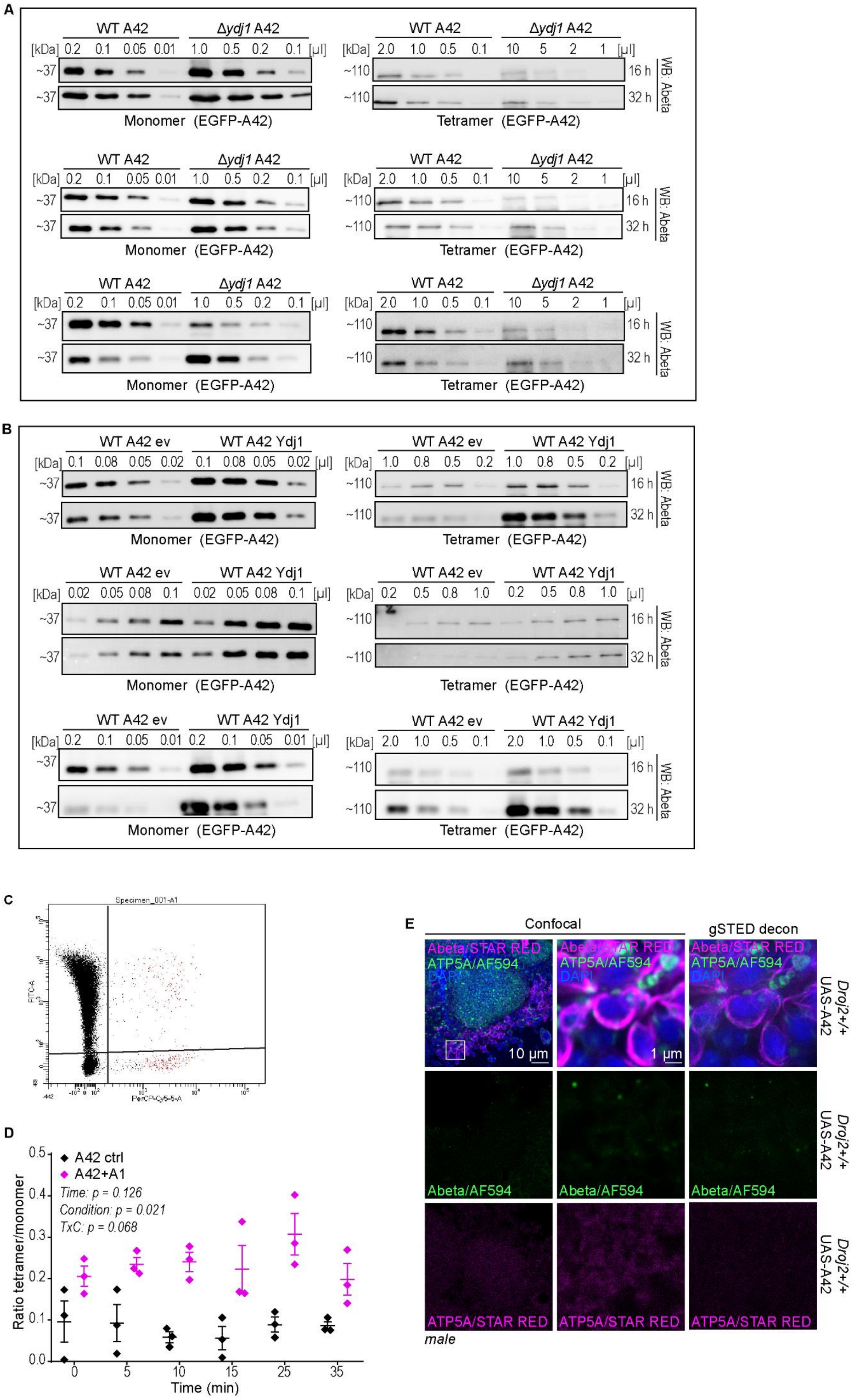
A
WT A42
Δydj1 A42
[kDa] 0.2 0.1 0.05 0.01 1.0 0.5 0.2 0.1 [µl]
[kDa] 2.0 1.0 0.5 0.1 10 5 2 1 [µl]
~37
~110
16 h
32 h
WB: Abeta
Monomer (EGFP-A42)
Tetramer (EGFP-A42)
B
WT A42 ev
WT A42 Ydj1
[kDa] 0.1 0.08 0.05 0.02 0.1 0.08 0.05 0.02 [µl]
[kDa] 1.0 0.8 0.5 0.2 1.0 0.8 0.5 0.2 [µl]
[kDa] 0.02 0.05 0.08 0.1 0.02 0.05 0.08 0.1 [µl]
[kDa] 0.2 0.5 0.8 1.0 0.2 0.5 0.8 1.0 [µl]
[kDa] 0.2 0.1 0.05 0.01 0.2 0.1 0.05 0.01 [µl]
[kDa] 2.0 1.0 0.5 0.1 2.0 1.0 0.5 0.1 [µl]
Monomer (EGFP-A42)
Tetramer (EGFP-A42)
C
Specimen_001-A1
FITC-A
PerCP-Cy5-5-A
D
A42 ctrl
A42+A1
Time: p = 0.126
Condition: p = 0.021
TxC: p = 0.068
Ratio tetramer/monomer
Time (min)
0 5 10 15 25 35
E
Confocal
gSTED decon
Abeta/STAR RED
ATP5A/AF594
DAPI
10 µm
1 µm
Droj2+/+
UAS-A42
Abeta/AF594
ATP5A/STAR RED
male

Appendix Figure S2

(**A-B**) Immunoblots used for EGFP-A42 tetramer/monomer ratio determination in Fig 3J and 3K in wild type (WT) and Δ*ydj1* cells expressing EGFP-A42 (A42) or EGFP only (ev) with co-expression of Ydj1 or corresponding empty vector control (ev) at indicated time points. Each sample probed in 4 dilutions (to ensure linear intensity) using Abeta-specific antibody (Abeta) 6E10.

(**C**) Immunoblot of whole cell extract (WCE) of wild type (WT) yeast cells after 16 h of expression of EGFP constructs. EGFP antibody (GFP) was used for immunoblotting.

(**D**) Quantification of the tetramer to monomer ratio of synthetic Abeta42 with or without DnaJA1 obtained from immunoblots representatively shown in (Fig 4F). Dot plots show all data points along with the mean (line) ± s.d.. n = 3 biologically independent cultures. P-values by two-way repeated measures ANOVA followed by simple main effects (*, $p < 0.05$, vs. control). See also Fig 4G.

(**E**) Representative confocal and gSTED deconvolved (decon) microscopy of Kenyon cells in 15-day-old male fly brains immunostained with mouse monoclonal IgG1 κ anti-Aß antibody (Abeta) clone 6E10 and mouse IgG2b anti mitochondrial marker ATP5A-specific antibody in combination with the fluorophore-conjugated secondary antibodies against specific mouse IgG subtypes STAR RED FluoTag-X2 sdAb anti-Mouse IgG1 (magenta) and Alexa Fluor 594 Goat anti-Mouse IgG2b (green) of w^{1118} wild type flies (*Droj2+/+*) expressing human Abeta42 (UAS-A42). Single staining with swapping the secondary antibodies was used to test cross reactivity. Co-staining with DAPI (blue) was used for nuclei staining.

Appendix Table S1: Primers used for cloning of pESC-his-EGFP and pESC-ura-FLAG constructs

Primer specific for:	Sequence
EGFP empty vector (fwd)	5'-ATCTGAATTCATGTCTAAAGGTGAAGAATTATTCAC-3'
EGFP empty vector (rev)	5'-ATCTGAATTCTTTGTACAATTCATCCATACCATG-3'
Clon. pESC-his-EGFP_G (fwd)	5'-ATCTGAATTCATGTCTAAAGGTGAAGAATTATTCAC-3'
Clon. pESC-his-EGFP_G (rev)	5'-ATCTGAATTCGTTTGTACAATTCATCCATACCATG-3'
A42 and *A42m2* (fwd)	5'-ATCTACTAGTATGGATGCAGAATTCCGACATGAC-3'
A42 and *A42m2* (rev)	5'-ATCTATCGATTTACGCTATGACAACACCGCCC-3'
C57 (fwd)	5'-ATCTACTAGTATGACAGTGATCGTCATCACCTTG-3'
C57 (rev)	5'-ATCTATCGATTTAGTTCTGCATCTGCTCAAAGAAC-3'
A40 (fwd)	5'-ATCTACTAGTATGGATGCAGAATTCCGACATGAC-3'
A40 (rev)	5'-ATCTATCGATTTAGACAACACCGCCCACCATG-3'
Ydj1 (fwd)	5'-ATCTACTAGTATGGTTAAAGAAACTAAGTTTTACG-3'
Ydj1 (rev)	5'-ATCTATCGATGTTTGAGATGCACATTGAACAC-3'
DnaJA1 (fwd)	5'-ATCTACTAGTATGGTGAAAGAAACAACTTACTACGATG-3'
DnaJA1 (rev)	5'-ATCTATCGATGT AGAGGTCTGACACTGAACACC-3'

Appendix Table S2: Primers used for quantitative reverse transcription real-time PCR (RT-qPCR).

Primer specific for:	Sequence
A42 (forward)	5'-GAATTCCGACATGACTCAGGA-3'
A42 (reverse)	5'-CACCTTTGTTTGAACCCACA-3'
Droj2 (forward)	5'-GATGGCCAGAAGATCGTGTT-3'
Droj2 (reverse)	5'-GTGGAGTGCTCCTTCTCGTC-3'
Rpl32 (forward)	5'-CCCAAGGGTATCGACAACAG-3'
Rpl32 (reverse)	5'-GTTCGATCCGTAACCGATGT-3'
α-Tubulin (forward)	5'-TGTCGCGTGTGAAACACTTC-3'
α-Tubulin (reverse)	5'-AGCAGGCGTTTCCAATCTG-3'

Appendix Table S3: Hits from proteomic approach

Relative protein abundance of EGFP-Abeta42 (A42) *versus* empty vector (ev) expressing wild type yeast cells. The significant subset of all proteins (including cytosolic proteins attached to mitochondria) in a proteomics analysis of isolated mitochondria is depicted. Average log2 SILAC ratios of A42/ev of two independent proteome measurements are shown. Significance was determined using an outlier test (Significance A, p <0.003).

Gene names	AVERAGE log2 A42 /empty vector	Significance A
HSP26	2,871	1,0E-42
A42GFP	2,077	4,5E-23
HSP42	1,968	7,1E-21
HSP104	1,376	6,2E-11
GPG1	1,304	6,0E-10
EPS1	0,992	2,7E-06
MNN1	0,987	2,9E-06
GAS5	0,950	7,0E-06
HSC82;HSP82	0,939	8,9E-06

SSA1	0,935	9,6E-06
BIO5	0,865	4,3E-05
MEP1;MEP3	0,822	1,0E-04
DID2	0,822	1,0E-04
ENO1	0,787	2,0E-04
NPC2	0,773	2,6E-04
GAL7	0,769	2,8E-04
GDH1	0,763	3,2E-04
GAL2	0,752	3,9E-04
SSA2	0,749	4,1E-04
PRB1	0,742	4,6E-04
GAL1	0,728	5,9E-04
HBN1	0,718	7,1E-04
YDJ1	0,688	1,2E-03
TNA1	0,687	1,2E-03
KRE2	0,637	2,7E-03
ERG3	0,636	2,8E-03
CDC3	-0,427	2,8E-03
DAP2	-0,428	2,7E-03
PST2	-0,435	2,3E-03
SSO2	-0,464	1,2E-03
YCK2	-0,467	1,1E-03
YBR016W	-0,566	8,1E-05
CRP1	-0,577	6,1E-05
HER2	-0,592	3,8E-05
YCP4	-0,593	3,8E-05
RAS2	-0,596	3,4E-05
RFS1	-0,650	6,5E-06
RPL38	-0,841	6,1E-09
FMP43	-0,983	1,2E-11
CDC4	-1,248	8,9E-18
