# Supplementary material for: The HSP40 chaperone Ydj1 drives amyloid beta 42 toxicity
Source: EMBO Mol Med. 2022 Apr 4;14(5):e13952. doi: 10.15252/emmm.202113952 (PMC9081910; doi:10.15252/emmm.202113952)
Supplement: Supplementary file 2 — Expanded View Figures PDF [file EMMM-14-e13952-s006.pdf]

Expanded View Figures

Figure EV1. Human A42 shows mitochondria-associated toxicity dependent on Ydj1 levels.

A Immunoblot of total cytoplasmic post-nuclear supernatant (PNS), mitochondrial, microsomal, and cytosolic fractions of wild-type yeast cells after 18 h expression of EGFP-C57 (C57) using EGFP-specific antibody (GFP). Purity of fractions was tested with antibodies against Tom22 (mitochondria) and Pgk1 (cytosol).

B Immunoblot of whole-cell extract (WCE) of wild-type (WT) yeast cells after 16 h of expression of EGFP-A42. Samples were either kept at RT or heated to 95°C before loading on the SDS gel. Abeta-specific antibody (Abeta) 6E10 and EGFP antibody (GFP) were used for immunoblotting. GAPDH was used as a loading control.

C Fluorescence microscopy of wild-type (WT) yeast cells after 18 h of expression of EGFP-A42 (A42) and co-overexpressing Ydj1-FLAG (Ydj1) or EGFP empty vector (ev). Mitochondria were visualized with MitoTracker Red (magenta).

D, E Quantification of DHE>Eth. positive cells at indicated time points during chronological aging of wild-type (WT) cells and cells lacking mitochondrial DNA (Rho0), expressing EGFP-A42 (A42) or EGFP only (ev). Mean ± SD $n = 4$–6 biologically independent cultures. *P*-values by two-way repeated measures ANOVA followed by simple main effects (****P* < 0.001; ***P* < 0.01, versus control).

F Quantification of intensities of Ydj1-specific antibody (Ydj1) bands of the immunoblot representatively shown in G and Appendix Fig S1B, normalized to intensities of Pgk1-specific antibody bands (Pgk1). Dot plots show all data points along with the mean (line) ± SD $n = 3$ biologically independent cultures. ***P* < 0.01; **P* < 0.05. ANOVA with Tukey's *post hoc* test.

G Immunoblot of whole-cell extract (WCE) of wild-type yeast cells after 16 h of expression of EGFP-A42. Pgk1 was used as a loading control. See also Appendix Fig S1B.

H Immunoblot of whole-cell extract (WCE) of wild-type and Δ*ydj1* yeast cells after 16 h of expression of EGFP-A42 and co-overexpressing Ydj1-FLAG (Ydj1) or harboring the corresponding vector controls. DnaJA1- and Abeta-specific (6E10) antibodies were used for immunoblots. GAPDH was used as a loading control.

I Quantification of PI positive wild-type (WT) and Δ*ydj1* cells after 42 h of expressing alpha-synuclein or harboring the empty vector control (ev). Dot plots show all data points along with the mean (bar) ± SD $n = 6$ biologically independent cultures. ****P* < 0.001. ANOVA with Tukey's *post hoc* test.

Source data are available online for this figure.

© 2022 The Authors

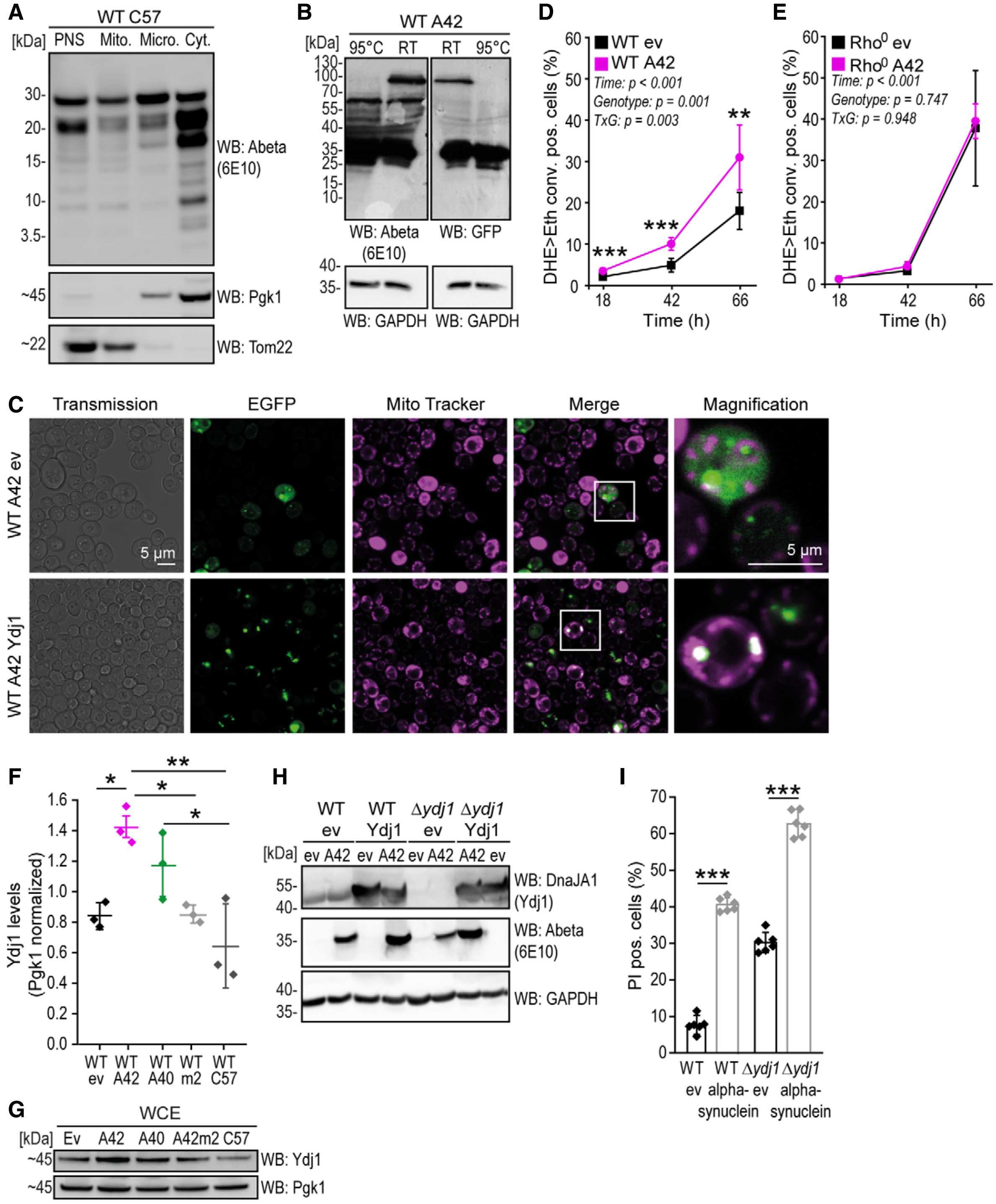

Figure EV1.

 © 2022 The Authors

Figure EV2. Validation of Ydj1 effects toward Abeta-induced phenotypes in alternative AD yeast model expressing Kar2-A42 guiding Abeta42 to the secretory pathway.

A, B Quantification of PI positive cells at indicated time points during chronological aging of wild-type (WT) (A) and Δ*ydj1* (B) cells expressing Kar2-A42 or harboring the empty vector control (ev). Mean ± SD $n = 6$ biologically independent cultures. *P*-values by two-way repeated measures ANOVA followed by simple main effects (****P* < 0.001; ***P* < 0.01, versus control).

C Immunoblot of whole-cell extract (WCE) of wild type (WT) after 16 h of expression of Kar2-A42 and expressing Ydj1-FLAG (Ydj1) or harboring the corresponding vector control (ev). Samples were either kept at RT or heated to 95°C before loading on the 4–12% NuPage Bis–Tris gel. Abeta-specific antibody (Abeta) 6E10 was used for immunoblotting. GAPDH was used as a loading control.

D Quantification of the ratio between A42 low-n oligomers and high-n oligomers in wild-type (WT) and *YDJ1* deletion strain (Δ*ydj1*) expressing Kar2-A42 as well as between wild-type (WT) expressing Kar2-A42 only or co-overexpressing Ydj1-FLAG (Ydj1) after 16 h of expression from immunoblots representatively shown in (C). Dot plots show all data points along with the mean (bar) ± SD $n = 6$ biologically independent cultures. ****P* < 0.001. ANOVA with Tukey's *post hoc* test.

E Immunoblot of total cytoplasmic post-nuclear supernatant (PNS), mitochondrial, microsomal, and cytosolic fractions of wild-type yeast cells after 18 h expression of Kar2-Abeta42 (A42) and empty vector (ev) using Abeta-specific antibody (WB: Abeta) 6E10. Purity of fractions was tested with antibodies against Tom22 (mitochondria), Sss1 (microsomes), and Pgk1 (cytosol).

F, G Quantification of PI positive cells at indicated time points during chronological aging of wild-type (WT) cells expressing EGFP-A42 or harboring the empty vector control (ev) upon treatment with Hsp70/Hsp40 interaction inhibitor 116-9e (G) or corresponding DMSO control (F). Mean ± SD $n = 6$ biologically independent cultures. *P*-values by two-way repeated measures ANOVA followed by simple main effects (***P* < 0.01; **P* < 0.05, versus control).

H Immunoblot of whole-cell extract (WCE), supernatant, and eluate of FLAG-tagged Ydj1. Immunoprecipitation (IP: FLAG) of wild-type (WT) cells expressing EGFP-C57 (C57) or EGFP only (ev) and co-overexpressing Ydj1-FLAG (Ydj1) using EGFP antibody (GFP) and FLAG antibody (FLAG).

I Immunoblot of whole-cell extract (WCE), eluate, and supernatant of FLAG-tagged Ydj1. Immunoprecipitation (IP: FLAG) of wild-type (WT) cells expressing EGFP-A42 (A42), A42m2, or EGFP only (ev) and co-overexpressing Ydj1-FLAG (Ydj1) using Abeta-specific antibody (Abeta) 6E10 and FLAG antibody (FLAG).

J Confocal microscopy of wild-type (WT) yeast cells expression of EGFP-A42 (A42) after 18 h of expression. Mitochondria were visualized with MitoTracker Red (magenta).

Source data are available online for this figure.

© 2022 The Authors

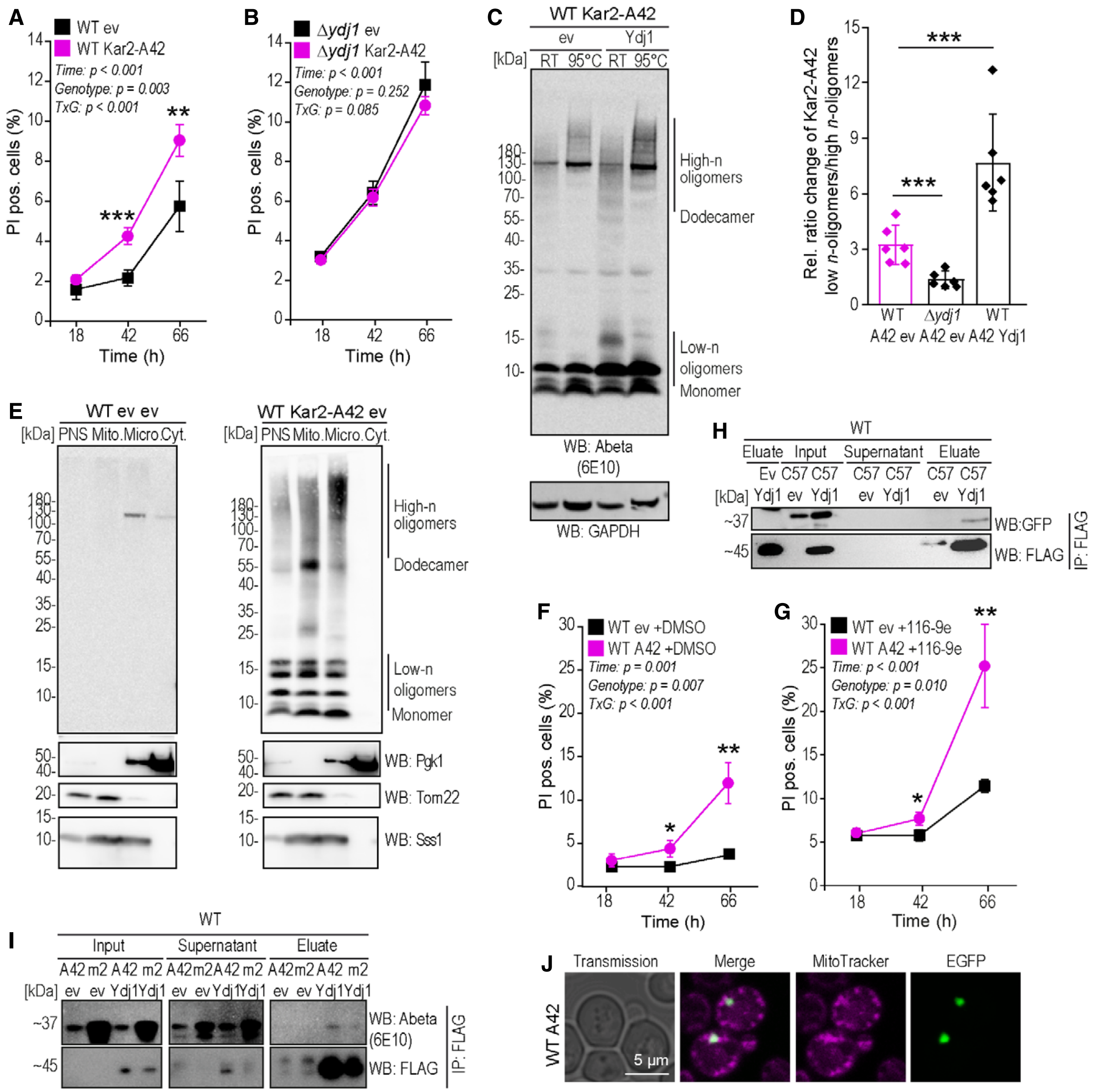

Figure EV2.

© 2022 The Authors

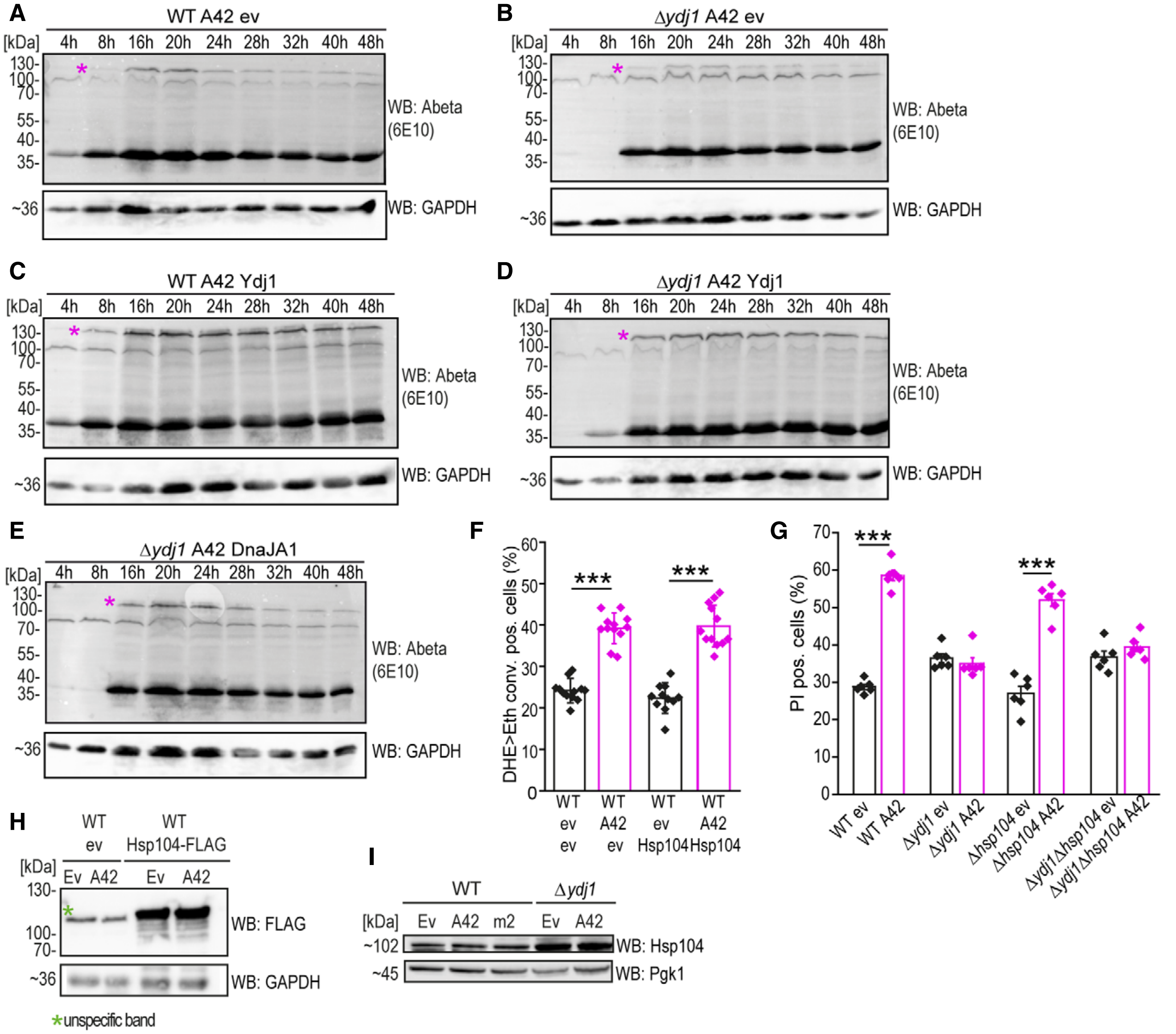

Figure EV3. Ydj1/DnaJA1 effects toward Abeta oligomerization and cytotoxicity are independent of Hsp104 levels.

A–D Immunoblot of time series of whole-cell extract (WCE) of wild-type (WT) and *YDJ1* deletion (Δ*ydj1*) cells expressing Ydj1 or harboring the corresponding empty vector control (ev) after indicated hours (h) of expression of EGFP-A42 using Abeta-specific antibody (Abeta) 6E10. Star* indicates the tetramer.

E Immunoblot of time series of Abeta42 expression in *YDJ1* deletion (Δ*ydj1*) cells co-expressing DnaJA1 after indicated hours (h) of expression of EGFP-A42 using Abeta-specific antibody (6E10). Star* indicates the tetramer.

F Quantification of DHE>Eth. positive wild type (WT) after 66 h of expressing EGFP-A42 (A42) and Hsp104 or harboring the corresponding empty vector controls (ev). Dot plots show all data points along with the mean (bar) ± SD $n = 12$ biologically independent cultures. ***$P < 0.001$. ANOVA with Tukey's *post hoc* test.

G Quantification of PI positive wild-type (WT), Δ*ydj1*, Δ*hsp104*, and Δ*ydj1*Δ*hsp104* cells after 66 h of expressing EGFP-A42 (A42) or harboring the corresponding empty vector controls (ev). Dot plots show all data points along with the mean (bar) ± SD $n = 6$ biologically independent cultures. ***$P < 0.001$. ANOVA with Tukey's *post hoc* test.

H Immunoblot of whole-cell extract (WCE) of wild-type (WT) yeast cells after 16 h of expression of EGFP-A42 and Hsp104-FLAG using FLAG antibody (FLAG) and corresponding vector controls (ev). The Hsp104-FLAG is ~102 kDa. GAPDH is used as a loading control. Green star marks an unspecific band.

I Immunoblot of whole-cell extract (WCE) of wild-type and Δ*ydj1* cells expressing EGFP-A42 (A42), EGFP-A42m2 (m2), or EGFP only (ev) using Hsp104-specific antibody. Pgk1 is used as a loading control.

Source data are available online for this figure.

Figure EV4. Abeta42-mediated toxicity is dependent on the fly homologue of DnaJA1, Droj2, in an AD fly model.

A, B Representative confocal microscopy of (A) 10-day-old female fly brains immunostained with Abeta-specific antibody (Abeta) (6E10) (magenta) and reference DNA staining with DAPI (blue) of *Droj2* knockdown flies ($Droj2^{+/-}$) and corresponding isogenic w^{1118} wild-type flies ($Droj2^{+/+}$) expressing human Abeta42 (UAS-A42). (B) 10-day-old female and male fly brains immunostained with Abeta-specific antibody (Abeta) (6E10) (red) and reference DNA staining with DAPI (blue) of corresponding isogenic w^{1118} wild-type control flies ($Droj2^{+/+}$ *ctrl*) not expressing human Abeta42.

C, D Average intensity (C) and total area (D) of Abeta (6E10) signal from fly brain confocal images representatively shown in Fig EV4A from 12 brains of w^{1118} wild-type ($Droj2^{+/+}$) and knockdown ($Droj2^{+/-}$) female flies. Dot plots show all data points along with the mean (bar) ± SD $n = 12$. Unpaired, two-tailed *t*-test.

E Fly Droj2 is recognized by specific antibody of the human homologue DnaJA1. Immunoblot analysis of fly heads using DnaJA1-specific antibody (DnaJA1) and Abeta-specific antibody (6E10) of 3- to 6-day-old Droj2 knockdown flies ($Droj2^{+/-}$) and corresponding isogenic w^{1118} wild-type flies ($Droj2^{+/+}$) with expression of human Abeta42 (UAS-A42) or without, control (ctrl). Ponceau S was used as a loading control. Scheme of the Droj2 locus with the position of the Mi{MIC} Droj2MI08491 transposon. In the transcript, black rectangles indicate translated regions, lines indicate introns, and dashed lines indicate untranslated regions. Data are adopted from FlyBase.

F qPCR analysis of Droj2-mRNA levels of 3- to 6-day-old *Droj2* knockdown female flies ($Droj2^{+/-}$) without expressing human Abeta42 (ctrl) normalized to corresponding isogenic w^{1118} wild-type flies ($Droj2^{+/+}$). Reference gene is *Rpl32*. Dot plots show all data points along with the mean (line) ± SD $n = 3$ biologically independent experiments. *$P < 0.05$. One sample *t*-test against 1.

G Aversive associative memory performance 2 min after training of aged (18 days old) female Droj2 knockdown flies ($Droj2^{+/-}$) and corresponding isogenic w^{1118} wild-type flies ($Droj2^{+/+}$) both expressing human Abeta42 (UAS-A42) of six independent biological replicates. Dot plots show all data points along with the mean (bar) ± SD $n = 6$. Unpaired, two-tailed *t*-test.

Source data are available online for this figure.

 © 2022 The Authors

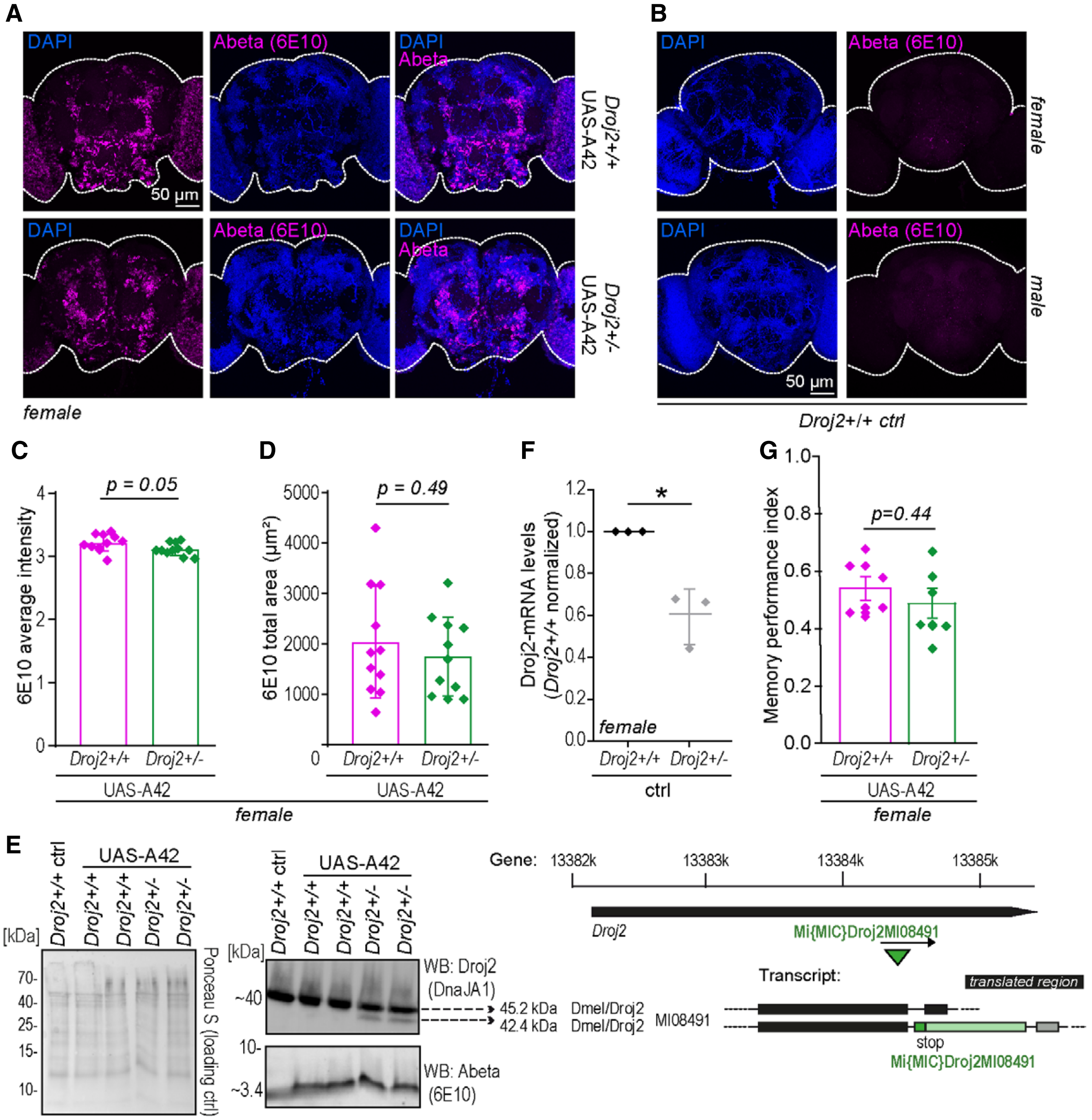
A
DAPI
Abeta (6E10)
DAPI Abeta
Droj2+/+ UAS-A42
Droj2+/- UAS-A42
50 µm
female
B
DAPI
Abeta (6E10)
female
male
50 µm
Droj2+/+ ctrl
C
p = 0.05
6E10 average intensity
Droj2+/+
Droj2+/-
UAS-A42
D
p = 0.49
6E10 total area (µm²)
Droj2+/+
Droj2+/-
UAS-A42
female
F
*
Droj2-mRNA levels (Droj2+/+ normalized)
female
Droj2+/+
Droj2+/-
ctrl
G
p=0.44
Memory performance index
Droj2+/+
Droj2+/-
UAS-A42
female
E
Droj2+/+ ctrl
UAS-A42
Droj2+/+
Droj2+/-
[kDa]
Ponceau S (loading ctrl)
WB: Droj2 (DnaJA1)
45.2 kDa Dmel/Droj2
42.4 kDa Dmel/Droj2
WB: Abeta (6E10)
Gene:
13382k
13383k
13384k
13385k
Droj2
Mi{MIC}Droj2MI08491
Transcript:
translated region
MI08491
stop
Mi{MIC}Droj2MI08491

Figure EV4.

Figure EV5. Abeta42-mediated toxicity is dependent on the fly homologue of DnaJA1, Droj2, in an AD fly model.

A Pearson's correlation coefficient and Manders' co-localization Coefficients of endoplasmatic reticulum protein KDEL and Abeta analyzed on confocal images of Kenyon cells in 15-day-old male fly brains immunostained with Abeta-specific antibody (Abeta) 6E10 and KDEL-specific antibody in Droj2 knockdown flies (*Droj2*$^{+/-}$) and corresponding isogenic w^{1118} wild-type flies (*Droj2*$^{+/+}$) with expression of human Abeta42 (UAS-A42). Dot plots show all data points along with the mean (bar) ± SD n = 10. Unpaired, two-tailed t-test.

B Representative confocal and gSTED deconvolved (decon) images of Kenyon cells in 15-day-old male fly brains immunostained with Abeta-specific antibody (Abeta) 6E10 (magenta) and endoplasmic reticulum protein KDEL-specific antibody (KDEL, green) of Droj2 knockdown flies (*Droj2*$^{+/-}$) and corresponding isogenic w^{1118} wild-type flies (*Droj2*$^{+/+}$) expressing human Abeta42 (UAS-A42).

C Representative confocal and gSTED deconvolved (decon) microscopy of Kenyon cells in 15-day-old male fly brains immunostained with Abeta-specific antibody (Abeta) 6E10 (magenta) and mitochondrial marker ATP5A-specific antibody (ATP5A, green) of w^{1118} wild-type flies (*Droj2*$^{+/+}$) expressing human Abeta42 (UAS-A42).

D–H Counts of mitochondria (D), mitochondria average size (E), mitochondria coverage area (F), Feret diameter (G), and min Feret diameter (H) of ATP5A-stained mitochondria from fly brain gSTED deconvolved images representatively shown in Fig 7C from 9 to 10 brains of w^{1118} wild-type (*Droj2*$^{+/+}$) and knockdown (*Droj2*$^{+/-}$) male flies expressing human Abeta42 (UAS-A42). Dot plots show all data points along with the mean (bar) ± SD n = 9–10. Unpaired, two-tailed t-test or Mann–Whitney test.

© 2022 The Authors

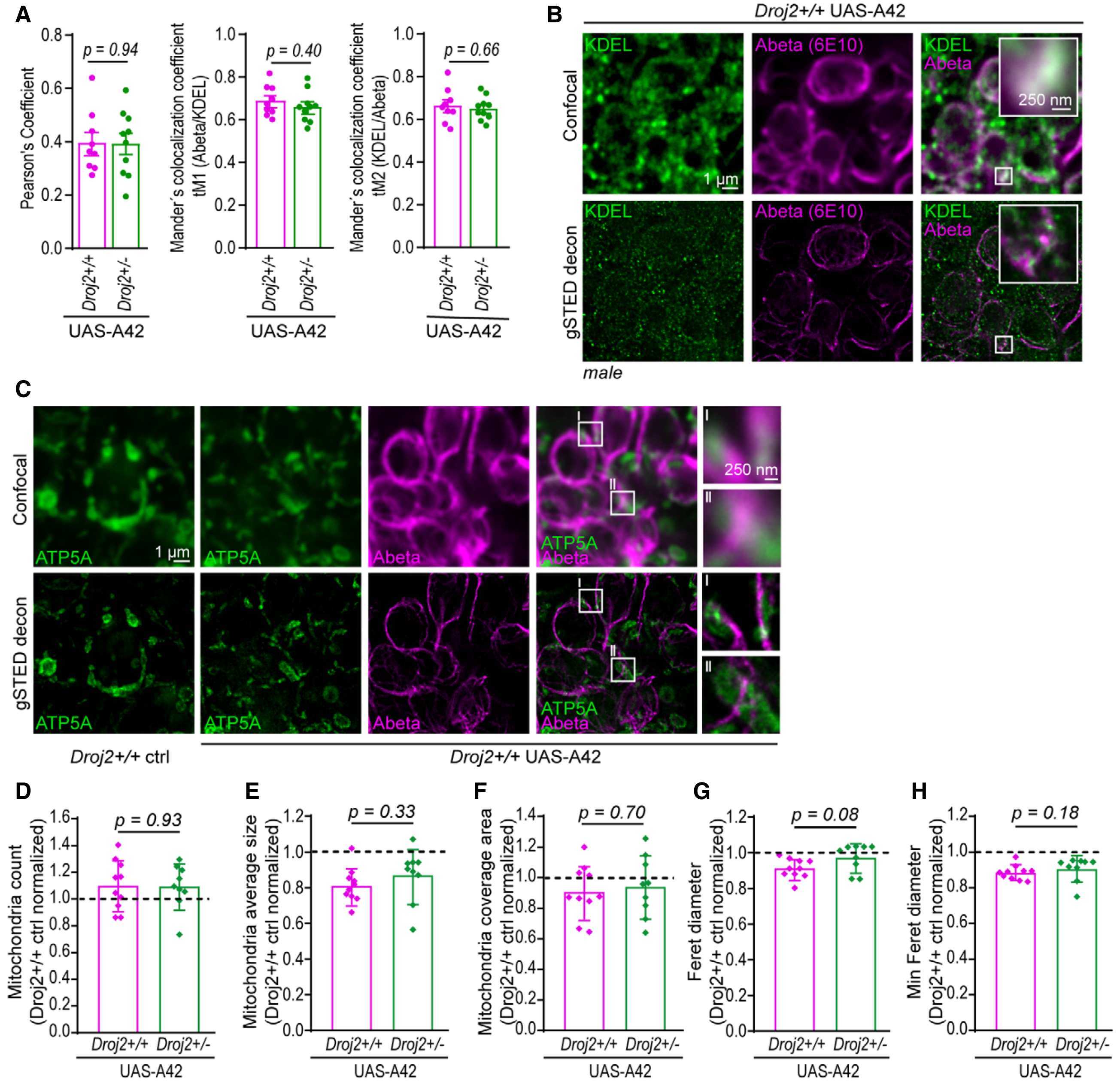
A
p = 0.94
Pearson's Coefficient
Droj2+/+
Droj2+/-
UAS-A42
p = 0.40
Mander´s colocalization coefficient tM1 (Abeta/KDEL)
p = 0.66
Mander´s colocalization coefficient tM2 (KDEL/Abeta)
B
Droj2+/+ UAS-A42
Confocal
gSTED decon
KDEL
Abeta (6E10)
Abeta
1 µm
250 nm
male
C
ATP5A
Abeta
Droj2+/+ ctrl
Droj2+/+ UAS-A42
D
p = 0.93
Mitochondria count (Droj2+/+ ctrl normalized)
E
p = 0.33
Mitochondria average size (Droj2+/+ ctrl normalized)
F
p = 0.70
Mitochondria coverage area (Droj2+/+ ctrl normalized)
G
p = 0.08
Feret diameter (Droj2+/+ ctrl normalized)
H
p = 0.18
Min Feret diameter (Droj2+/+ ctrl normalized)

Figure EV5.
