# Supplementary figures and images for: The HSP40 chaperone Ydj1 drives amyloid beta 42 toxicity
Source: EMBO Mol Med. 2022 Apr 4;14(5):e13952. doi: 10.15252/emmm.202113952 (PMC9081910; doi:10.15252/emmm.202113952)

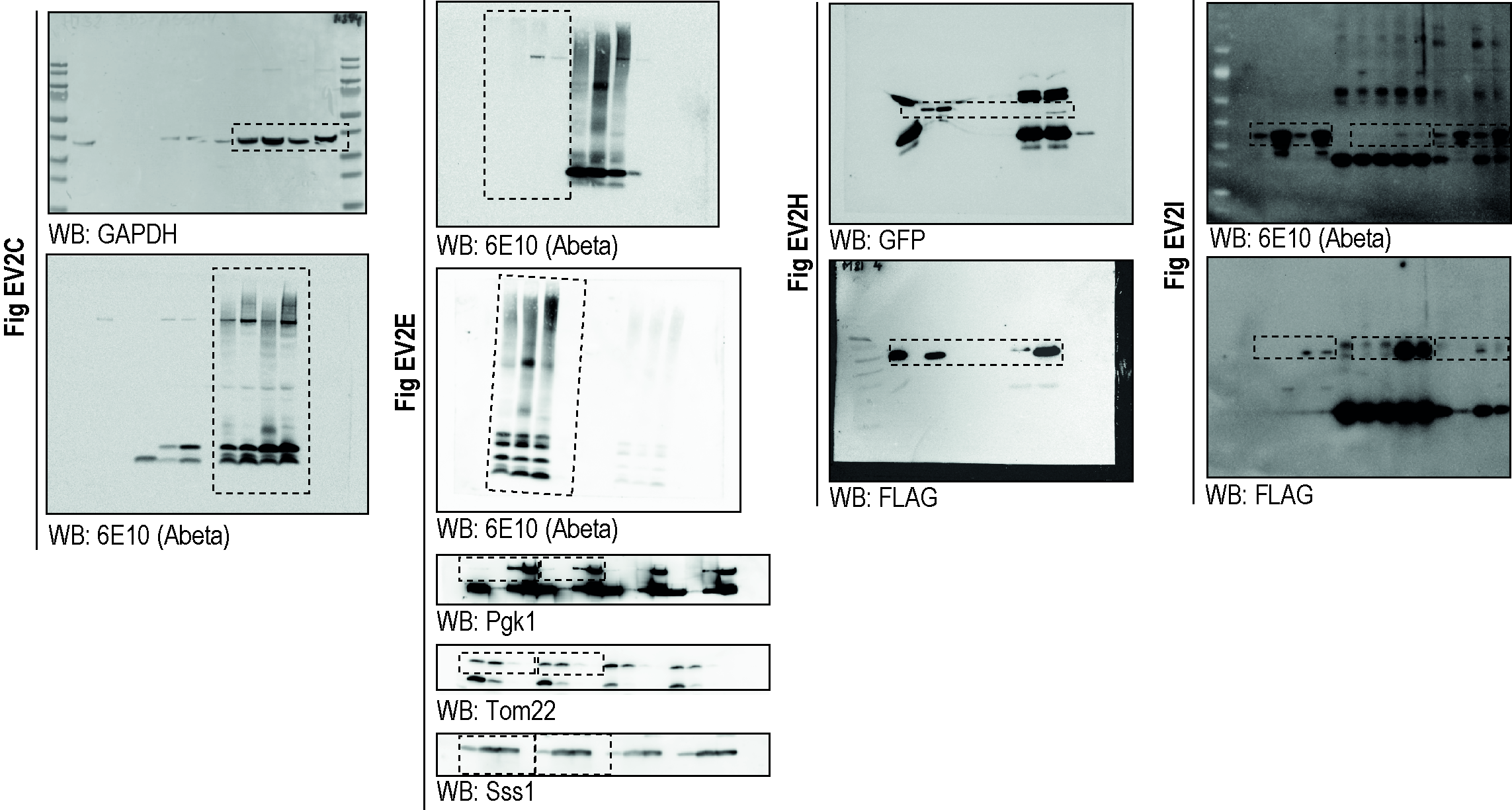

Supplement: Supplementary file 3 — Source Data for Expanded View and Appendix [file EMMM-14-e13952-s010.zip › EMM-2021-13952-V2_source_data_whole_immuno_blots_related_to_EV2.tif]

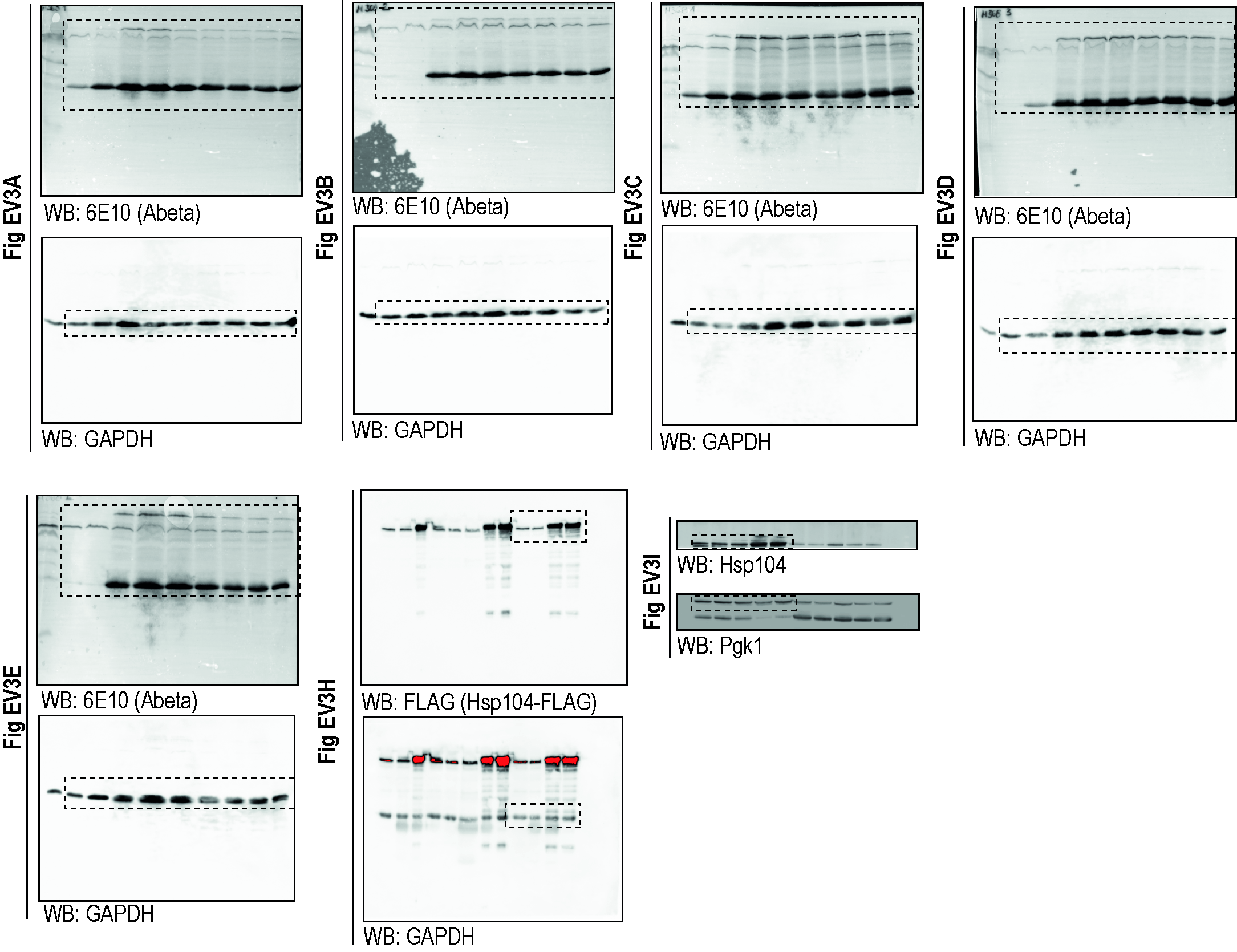

Supplement: Supplementary file 3 — Source Data for Expanded View and Appendix [file EMMM-14-e13952-s010.zip › EMM-2021-13952-V2_source_data_whole_immuno_blots_related_to_EV3.tif]

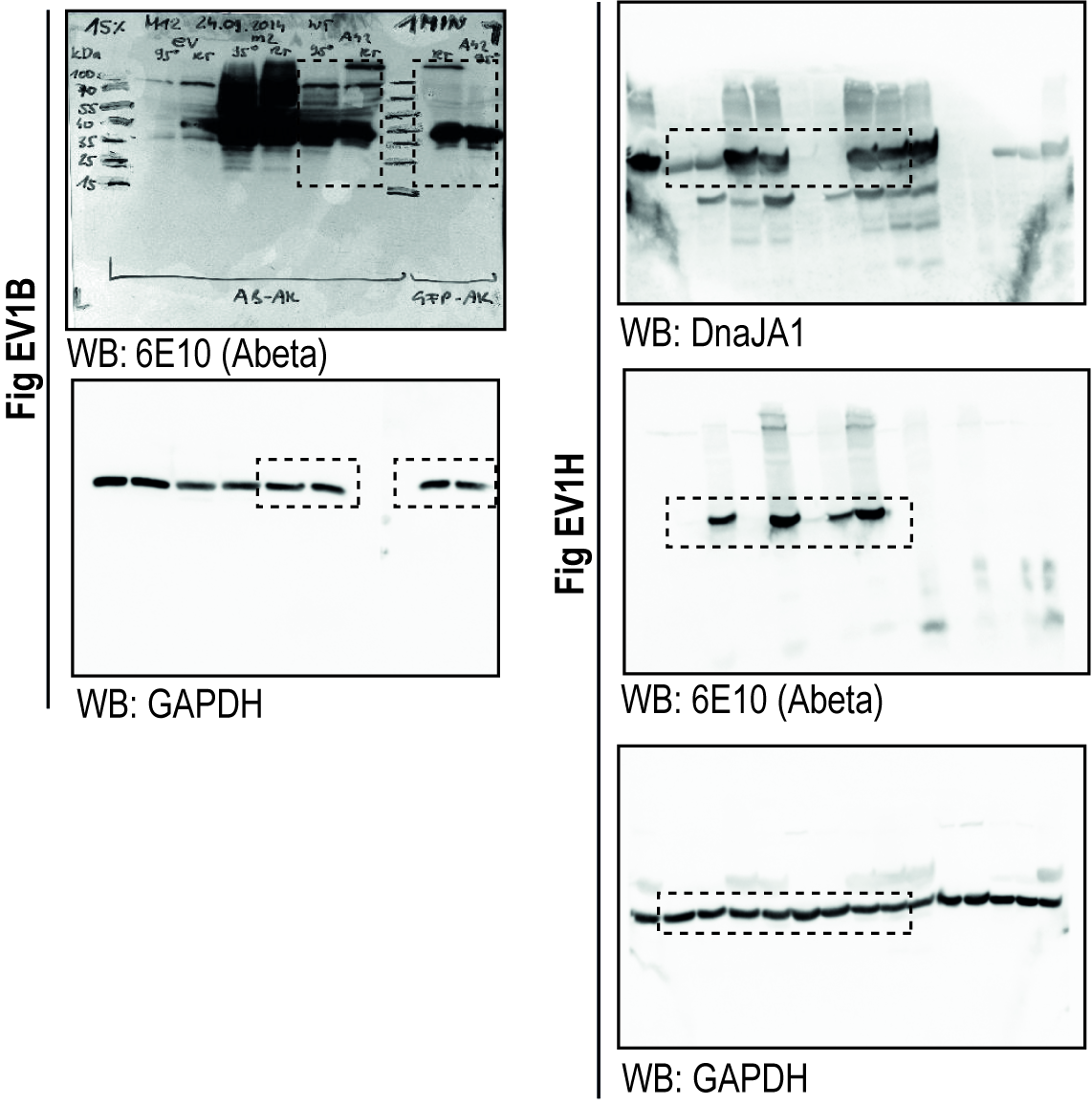

Supplement: Supplementary file 3 — Source Data for Expanded View and Appendix [file EMMM-14-e13952-s010.zip › EMM-2021-13952-V3-Figure_EV1_Source_Data-sd.tif]

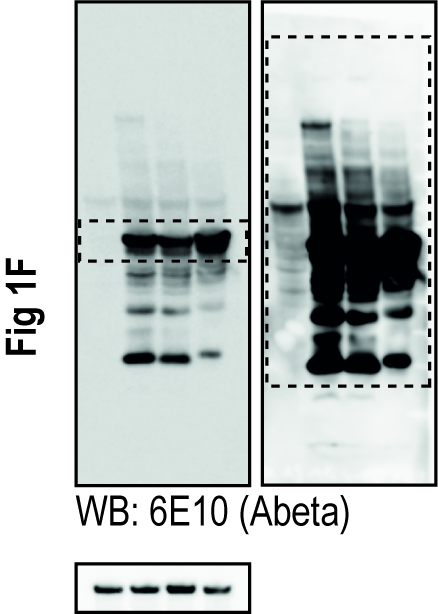

Supplement: Supplementary file 4 — Source Data for Figure 1 [file EMMM-14-e13952-s004.tif]

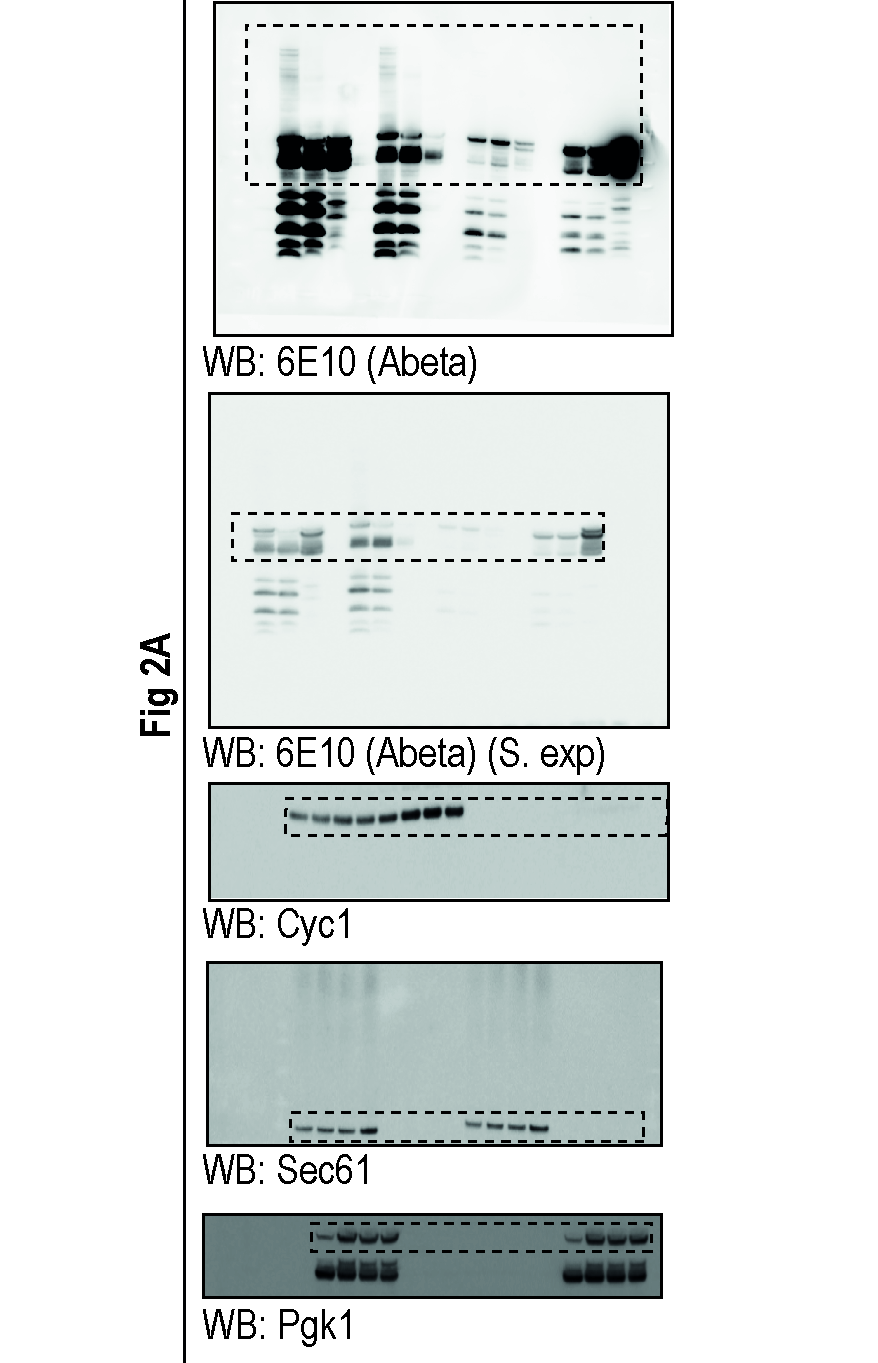

Supplement: Supplementary file 5 — Source Data for Figure 2 [file EMMM-14-e13952-s001.tif]

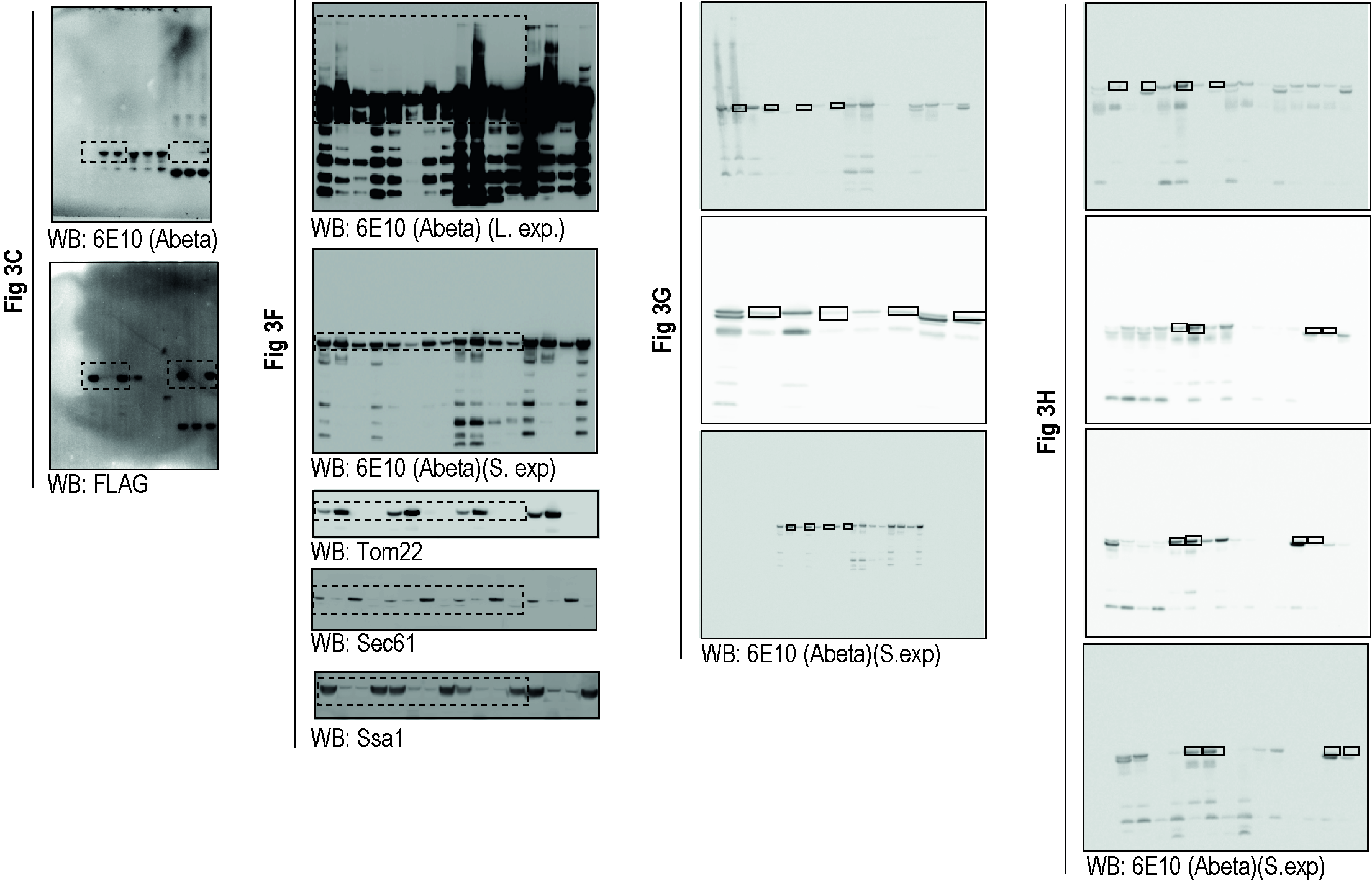

Supplement: Supplementary file 6 — Source Data for Figure 3 [file EMMM-14-e13952-s007.tif]

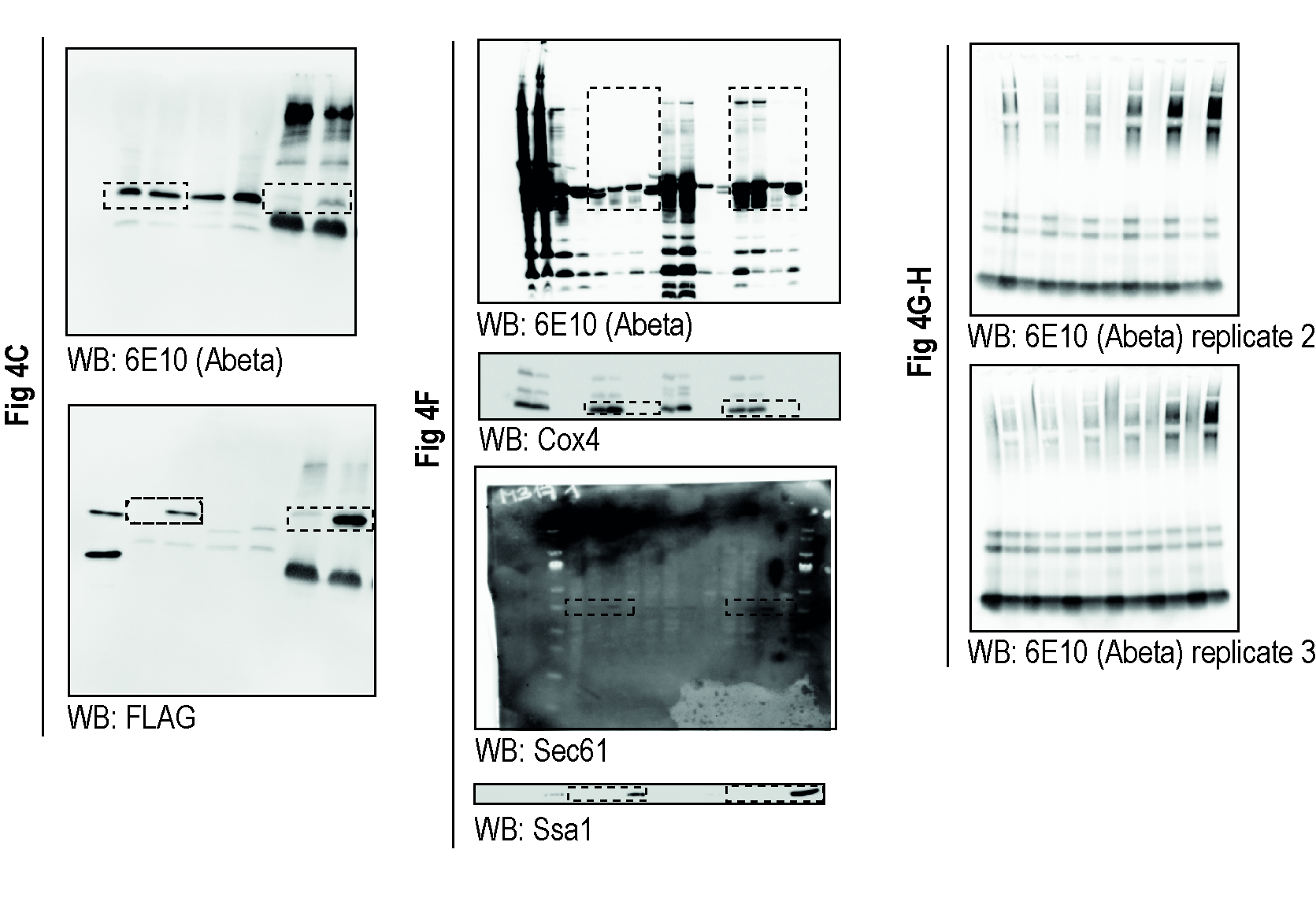

Supplement: Supplementary file 7 — Source Data for Figure 4 [file EMMM-14-e13952-s003.tif]

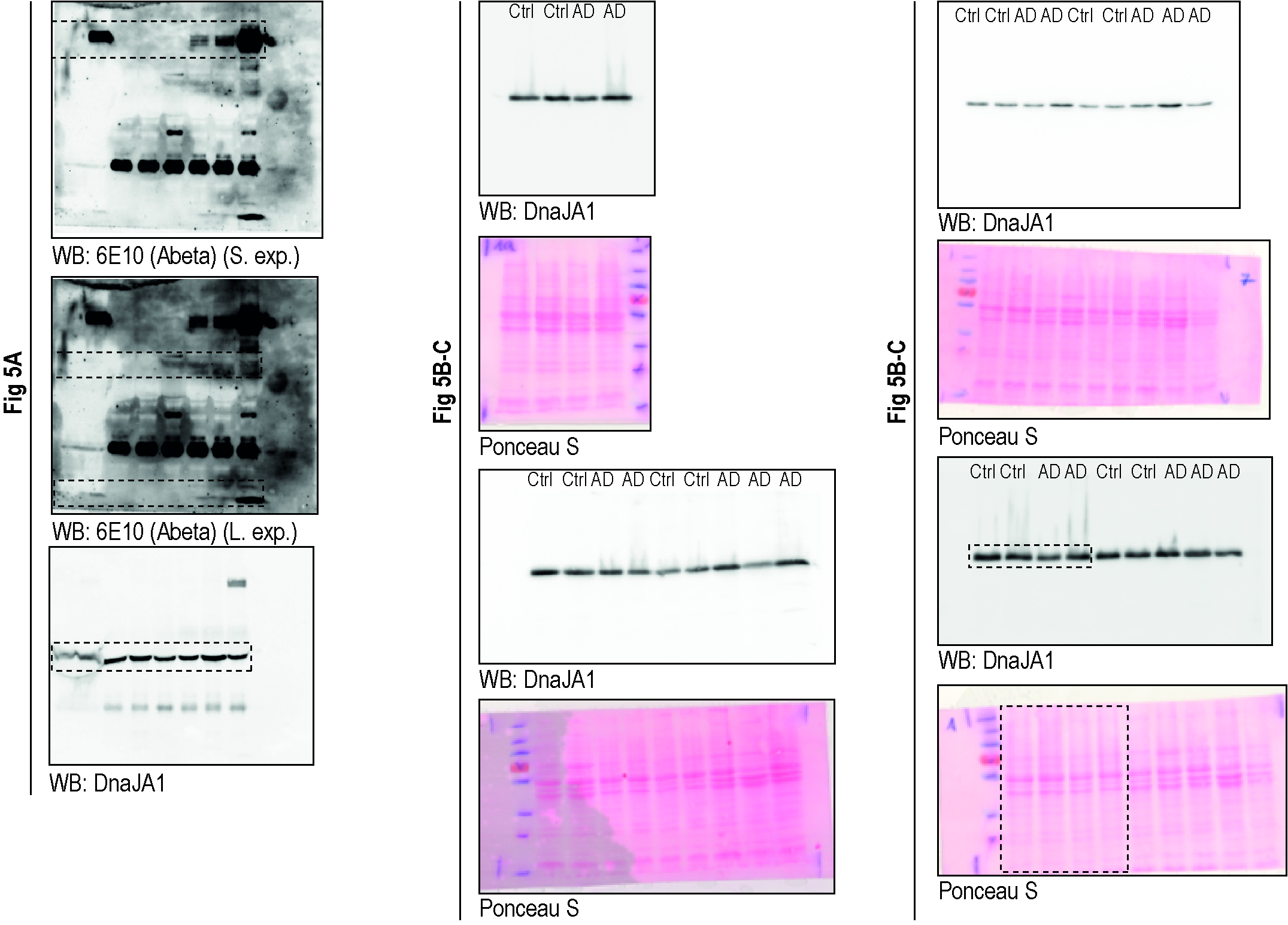

Supplement: Supplementary file 8 — Source Data for Figure 5 [file EMMM-14-e13952-s008.tif]

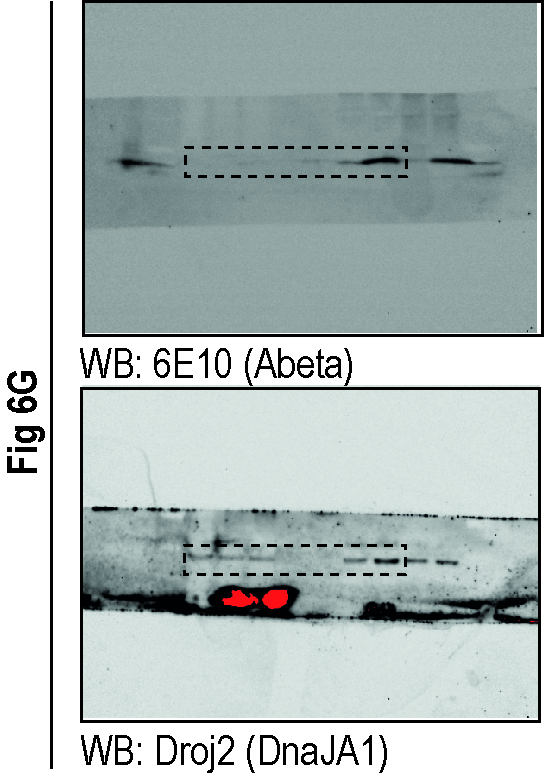

Supplement: Supplementary file 9 — Source Data for Figure 6 [file EMMM-14-e13952-s009.zip › emmm202113952-sup-0008-SDataFig6_new/EMM-2021-13952-V3-Figure_6_Source_Data-sd.tif]
