# Supplementary material for: The HSP40 chaperone Ydj1 drives amyloid beta 42 toxicity
Source: EMBO Mol Med. 2022 Apr 4;14(5):e13952. doi: 10.15252/emmm.202113952 (PMC9081910; doi:10.15252/emmm.202113952)
Supplement: Supplementary file 9 — Source Data for Figure 6 [file EMMM-14-e13952-s009.zip › emmm202113952-sup-0008-SDataFig6_new/EMM-2021-13952-V3-Figure_6F_Source_Data-sd.docx]

**Source Data for Figure 6F: CT values of qPCR.**

|  | | | | |
| --- | --- | --- | --- | --- |
| **Experiment** | **Primer** | **Strain** | **CT mean** | **Figure** |
|  |  |  |  |  |
| **12** | *Droj2* | *Droj2 +/+* | 30.824 | **6F** |
|  |  | *Droj2 +/+ UAS-*A42 | 30.548 |  |
|  | *Rpl32* | *Droj2 +/+* | 33.526 |  |
|  |  | *Droj2 +/+ UAS-*A42 | 33.824 |  |
| **13** | *Droj2* | *Droj2 +/+* | 28.908 |  |
|  |  | *Droj2 +/+ UAS-*A42 | 26.317 |  |
|  | *Rpl32* | *Droj2 +/+* | 28.033 |  |
|  |  | *Droj2 +/+ UAS-*A42 | 25.483 |  |
| **14** | *Droj2* | *Droj2 +/+* | 33.708 |  |
|  |  | *Droj2 +/+ UAS-*A42 | 30.651 |  |
|  | *Rpl32* | *Droj2 +/+* | 30.961 |  |
|  |  | *Droj2 +/+ UAS-*A42 | 28.079 |  |
