# Supplementary material for: The HSP40 chaperone Ydj1 drives amyloid beta 42 toxicity
Source: EMBO Mol Med. 2022 Apr 4;14(5):e13952. doi: 10.15252/emmm.202113952 (PMC9081910; doi:10.15252/emmm.202113952)
Supplement: Supplementary file 10 [file EMMM-14-e13952-s002.pdf]

EMBO Molecular Medicine

The HSP40 chaperone Ydj1 drives amyloid beta 42 toxicity

Julia Ring, Jelena Tadic, Selena Ristic, Michael Poglitsch, Martina Bergmann, Nemanja Radic, Dirk Mossmann, YongTian Liang, Marta Maglione, Andrea Jerkovic, Roozbeh Hajiraissi, Marcel Hanke, Victoria Küttner, Heimo Wolinski, Andreas Zimmermann,Lana Domuz, Leonie Mikolasch, Daiana Moretti, Filomena Broeskamp, Julia Westermayer, Claudia Abraham, Simon Schauer, Christopher Dammbrueck, Sebastian Hofer, Mahmoud Abdellatif, Guido Grundmeier, Guido Kroemer, Ralf Braun, Niklas Hansen, Cornelia Sommer, Mirjana Ninkovic, Sandra Seba, Patrick Rockenfeller, Friederike-Nora Voegtle, Jörn Dengjel, Chris Meisinger, Adrian Keller, Stephan Sigrist,Tobias Eisenberg, Frank Madeo
DOI: 10.15252/emmm.202113952

Corresponding author(s): Frank Madeo (frank.madeo@uni-graz.at) , Tobias Eisenberg (tobias.eisenberg@uni-graz.at)

Review Timeline:

Submission Date:	13th Jan 21
Editorial Decision:	17th Feb 21
Revision Received:	11th Jan 22
Editorial Decision:	1st Feb 22
Revision Received:	12th Feb 22
Accepted:	15th Feb 22

Editor: Jingyi Hou

Transaction Report:

(Note: With the exception of the correction of typographical or spelling errors that could be a source of ambiguity, letters and reports are not edited. Depending on transfer agreements, referee reports obtained elsewhere may or may not be included in this compilation. Referee reports are anonymous unless the Referee chooses to sign their reports.)

17th Feb 2021

Dear Prof. Madeo,

Thank you for the submission of your manuscript to EMBO Molecular Medicine. We have now heard back from the three referees whom we asked to evaluate your manuscript. As you will see from the reports below, the referees find the topic of your study of potential interest. However, they raise substantial concerns about your work, which should be convincingly addressed in a major revision of the present manuscript.

We think that it is essential to address referees' concerns with regard to the adequacy of the model systems, and some of the key findings need to be validated in additional models, as commented by Referee #3. Further, Referee #1 is concerned about the moderate impact of DNAJA1 on memory performance in flies and pointed out that inhibitor experiments would further enhance the translational implications of the study, which need to be satisfactorily addressed.

During our pre-decision cross-commenting process (in which the referees are given a chance to make additional comments, including on each other's reports), Referee #1 added, "My take on this paper is that it needs additional experiments to be performed in order to demonstrate the translational impact of the findings, particularly a stronger impact on memory performance in flies and the additional points raised by the other referees." Referee #3 said, "I think the key findings of the study need to be validated in more appropriate models (APP transgenic mice, cultured neurons) to assess the relevance for AD pathogenesis. The fly and C.elegans models used are very limited as they rely on expression of artificial Abeta constructs, and the topology of expression is not reflecting the endogenous situation." We will not ask you to validate the results in a mouse model, as this would likely not be feasible in a reasonable timeframe. However, some of the key results need to be validated in cultured neurons, mouse or human brains as suggested by Referee #3.

Overall it is clear that publication of the manuscript cannot be considered at this stage. I also note that addressing the referees' concerns in full will be necessary for further considering the manuscript in our journal and this appears to require a lot of additional work and experimentation. I am unsure whether you will be able or willing to address those and return a revised manuscript within the six months deadline. On the other hand, given the potential interest of the findings, I would be willing to consider a revised manuscript with the understanding that the referee concerns must be fully addressed and that acceptance of the manuscript would entail a second round of review. I should remind you that it is EMBO Molecular Medicine policy to allow a single round of revision only and that, therefore, acceptance or rejection of the manuscript will depend on the completeness of your responses included in the next, final version of the manuscript. For this reason, and to save you from any frustrations in the end I would strongly advise against returning an incomplete revision and would also understand your decision if you choose to rather seek rapid publication elsewhere at this stage.

Should you decide to embark in such a revision, revised manuscripts should be submitted within six months of a request for revision; they will otherwise be treated as new submissions, except under exceptional circumstances in which a short extension is obtained from the editor. Also, the length of the revised manuscript may not exceed 60,000 characters (including spaces) and, including figures, the paper must ultimately fit onto optimally ten pages of the journal. Should you find the length constraints to be a problem, you may consider including any peripheral data (but not methods in their entirety) in the form of Supplementary information.

I look forward to seeing a revised form of your manuscript as soon as possible.

Should you find that the requested revisions are not feasible within the constraints outlined here and choose, therefore, to submit your paper elsewhere, we would welcome a message to this effect.

Sincerely,
Jingyi

Jingyi Hou
Editor
EMBO Molecular Medicine

*** Instructions to submit your revised manuscript ***

IMPORTANT: EMBO Molecular Medicine is now Open Access (see Press Release https://bit.ly/AwAXU5 and updated Author

Guidelines https://www.embopress.org/page/journal/17574684/authorguide).

*** PLEASE NOTE *** As part of the EMBO Publications transparent editorial process initiative (see our Editorial at https://www.embopress.org/doi/pdf/10.1002/emmm.201000094), EMBO Molecular Medicine will publish a Review Process File online to accompany accepted manuscripts. When preparing your letter of response, please be aware that in the event of acceptance, your cover letter/point-by-point document will be included as part of this file, which will be available to the scientific community.
This would include any additional data, figures or scans made available to the editors or referees. If you do NOT want the file to be published or would like to exclude figures, please immediately inform the editorial office via e-mail. More information about this initiative is available in our Instructions to Authors. If you have any questions about this initiative, please contact the editorial office at contact@embomolmed.org.

To submit your manuscript, please follow this link:

https://embomolmed.msubmit.net/cgi-bin/main.plex

When submitting your revised manuscript, please include:

1) a .docx formatted version of the manuscript text (including Figure legends and tables). Please make sure that the changes are highlighted to be clearly visible to referees and editors alike.

2) separate figure files*

3) supplemental information as Expanded View and/or Appendix. Please carefully check the authors guidelines for formatting Expanded view and Appendix figures and tables at
https://www.embopress.org/page/journal/17574684/authorguide#expandedview

4) a letter INCLUDING the reviewer's reports and your detailed responses to their comments (as Word file).

Also, and to save some time should your paper be accepted, please read below for additional information regarding some features of our research articles:

5) The paper explained: EMBO Molecular Medicine articles are accompanied by a summary of the articles to emphasize the major findings in the paper and their medical implications for the non-specialist reader. Please provide a draft summary of your article highlighting
- the medical issue you are addressing,
- the results obtained and
- their clinical impact.
This may be edited to ensure that readers understand the significance and context of the research.
Please refer to any of our published articles for an example.

6) For more information: There is space at the end of each article to list relevant web links for further consultation by our readers. Could you identify some relevant ones and provide such information as well? Some examples are patient associations, relevant databases, OMIM/proteins/genes links, author's
websites, etc...

7) Author contributions: the contribution of every author must be detailed in a separate section.

8) EMBO Molecular Medicine now requires a complete author checklist (https://www.embopress.org/page/journal/17574684/authorguide) to be submitted with all revised manuscripts. Please use the checklist as guideline for the sort of information we need WITHIN the manuscript as well as in the checklist. This is particularly important for animal reporting, antibody dilutions (missing) and exact p-values and n that should be indicated instead of a range.

9) Every published paper now includes a 'Synopsis' to further enhance discoverability. Synopses are displayed on the journal webpage and are freely accessible to all readers. They include a short stand first (maximum of 300 characters, including space) as well as 2-5 one sentence bullet points that summarise the paper. Please write the bullet points to summarise the key NEW findings. They should be designed to be complementary to the abstract - i.e. not repeat the same text. We encourage inclusion of key acronyms and quantitative information (maximum of 30 words / bullet point). Please use the passive voice. Please attach these in a separate file or send them by email, we will incorporate them accordingly.

You are also welcome to suggest a striking image or visual abstract to illustrate your article. If you do please provide a jpeg file 550 px-wide x 400-px high.

10) A Conflict of Interest statement should be provided in the main text

11) Please note that we now mandate that all corresponding authors list an ORCID digital identifier. This takes <90 seconds to complete. We encourage all authors to supply an ORCID identifier, which will be linked to their name for unambiguous name identification.

Currently, our records indicate that there is no ORCID associated with your account.

Please click the link below to provide an ORCID:
Link Not Available

12) The system will prompt you to fill in your funding and payment information. This will allow Wiley to send you a quote for the article processing charge (APC) in case of acceptance. This quote takes into account any reduction or fee waivers that you may be eligible for. Authors do not need to pay any fees before their manuscript is accepted and transferred to our publisher.

*Additional important information regarding Figures

https://bit.ly/EMBOPressFigurePreparationGuideline

Each figure should be given in a separate file and should have the following resolution:
Graphs 800-1,200 DPI
Photos 400-800 DPI
Colour (only CMYK) 300-400 DPI"

Figures are not edited by the production team. All lettering should be the same size and style; figure panels should be indicated by capital letters (A, B, C etc). Gridlines are not allowed except for log plots.
Figures should be numbered in the order of their appearance in the text with Arabic numerals. Each Figure must have a separate legend and a caption is needed for each panel.

***** Reviewer's comments *****

Referee #1 (Comments on Novelty/Model System for Author):

The findings are novel but the medical impact is currently low to medium as more experiments are needed about testing memory in flies.

Referee #1 (Remarks for Author):

In this study, the authors performed a series of elegant experiments in yeast and fly models to conclude that DNAJA1 may function as a driver of AB42 toxicity via mitochondrial translocation. The conclusion about cytotoxicity is supported by a convincing body of data. However, additional experiments are needed to firmly establish the importance of these findings for the regulation of behavior/memory.

Major concerns

A critical question raised is whether the role of DNAJA1 in driving the cellular AB cytotoxicity as inferred using yeast and flies might be important for memory and cognitive performance. This question is solely addressed in flies (Fig. 5J) and not in higher order models such as mouse models. In Fig 5J, the increase of memory performance in Droj2+/- UAS-A42 male flies compared to control flies is moderate (small amplitude with $p < 0.05$), suggesting that the role of DNAJA1 in driving AB42 cytotoxicity may have little impact on memory loss in AD. More experiments are needed to evaluate the importance of DNAJA1 for memory, particularly as the authors did not use mice. One possibility is to search for genetic enhancers of memory defects in Droj2+/- UAS-A42 flies, for example by using a candidate gene approach (e.g. a gene network-guided approach), to determine if such enhancers might lie in the HSP pathways. Another possibility is to enhance the sensitivity of the assay in flies, testing for the speed of odor choice (using video recording) in order to complement the current data about the result of odor choice. Lastly, it is unclear why the authors elected to not test for memory in female flies: why would the increased manganese stress resistance in female flies bearing Droj2 reduction prevent testing these flies for memory performance?

Additionally, there is no evidence for the effect on memory to be mediated by the expected target, that is lesser mitochondrial translocation in the brain of AB42 flies bearing Droj2 reduction compared to that of AB42 flies.

The discussion on the unexpected role of DNAJA1 in driving AB cytotoxicity compared to that of HPSs in opposing cytotoxicity needs to be expanded, discussing this finding in the context of the knowledge on the role of Tau, notably phospo Tau, compared to that of AB42 in driving AD, also discussing this finding in the context of other well-studied neurodegenerative diseases such

as for example Huntington's disease.

Minor points:

It is a missed opportunity that the authors did not test for the effects of chemical DNAJA1 inhibitors in flies (unless there are some issues with the access to or the specificity and use of these compounds), as this would provide a basis for similar testing in mouse models of AD and other neurodegenerative diseases.

Referee #2 (Comments on Novelty/Model System for Author):

The choice of yeast model--an episomal plasmid expression system--is not very well justified and adequate controls are not included to allow the reader to interpret the data. These have been detailed in my remarks to authors below.

Referee #2 (Remarks for Author):

This paper describes work in two model systems to show that the presence of mitochondrial HSP40 chaperone, Ydj1p in yeast and DNAJA1 in humans, exacerbates the detrimental effects of amyloid-beta42, through the modulation of its aggregation state. I appreciate the use of multiple model systems to illustrate the findings in this work. This finding is potentially interesting, especially with the connection to oligomerization. However, the work could be strengthened by adding multile controls that would allow the reader to interpret the data and substantiate the authors' claims.

MAJOR POINTS
1. It appears that yeast models in this paper rely on episomal plasmids. These are inherently variable in copy number and strains expressing toxic proteins can select against high copy number clones. This makes many of the results throughout this paper difficult to interpret. That difficulty is compounded by the lack of loading controls throughout multiple western blots of amyloid protein levels and aggregation extent. The authors should justify this choice of using episomal plasmids or perform a few key experiments with copy number-controlled, integrated or centromeric strains to show a true peptide/sequence-specific effects with accompanying western blots.
2. The DHE metric for cell death/oxidative stress is relevant, but for comparison to past yeast literature, it would be helpful to provide standard growth curves (OD600) or spotting assays for all lines used throughout the paper. This would allow readers to be able to process all these finding in the context of other yeast neurodegeneration literature.
3. The authors have a very powerful set of controls in C57 , Abeta40, and Abeta42m2. However, they could use them more broadly to prove their points about Abeta42. For example, given that Abeta40 is similarly toxic to Abeta42 in their model (S1A), but doesn't have the tetrameric form that the authors suggest is important for Ydj1p activity (1F), it would be a good control to decouple oligomerization from toxicity. Also, the other forms C57, and Abeta42m2 could be used to explore effects of non-toxic cytosolic peptide expression (for example, localization etc...). Again, this is best done in a copy-number- or expression-controlled setting.
4. There have been reports of multimerization of EGFP (also evidenced in Fig S1D)-it would greatly improve the claims of multimerization if a monomeric version of GFP were used like monomeric superfolder GFP, for example. It would be more convincing if the authors could repeat a few of the imaging experiments and multimerization experiments either with antibody ICC (on an untagged strain) or another known monomeric fluorescent tag. This would suppress doubts that the EGFP tag, which far exceeds the size of the abeta peptide itself, is modifying this behavior or enhancing aggregation potential.
5. Although many of the findings in this paper are interesting, it is hard to know how many of the observations result from cytosolic expression. The Kar2-Abeta yeast model that the authors include in some selective experiments could better reflect the more physiological context for Abeta - in the secretory pathway (still intracellular)-rather than cytosolic. And differences between the Kar2-abeta and the cytosolic abeta strain can give insight into what effects are specific to the secretory pathway and which are specific to cytosolic expression. Therefore, I suggest the authors include the comparison for the Kar2-Abeta strain throughout the paper in multiple experiments to bolster their points. This will also complement well the results from the Drosophila model as it also uses a secretory-pathway directed Abeta construct.
6. There's an interesting difference between the time-dependence of Abeta42 expression on PI-positive cells (3A,B) compared to the ydj1-deletion strains. It looks like at 42h, ydj1 exacerbates the toxic abeta phenotype, whereas it may suppress at 66h. The ydj1 deletion seems to do this for the WT background too. Interestingly another interesting dynamic change in toxicity suppression can be observed in the Rho0 strains (S1G). Could the authors comment on all of these dynamics? Perhaps how they relate to respiratory state of the cells?
7. Given that ydj1 deletion seems toxic by itself (3A,B, S1J,K), how does one deconvolute these effects of toxicity additivity?
8. A missing strain from panels 3F & I is the YDJ1 overexpression in the WT background. It would be great if the authors could add this and the quantification of the blot. It seems like the authors have performed this type of experiment in some contexts (3H), but even for this context, the blot is only in S5 and is tiny without lane labels to allow for easy interpretation.
9. Sis1 conspicuously absent-Sis1 belongs to different class and suppresses toxicity of TDP-43 (Liebman lab, PLoS Genetics, 2017); would be interesting to know its effect here.
10. It would help the manuscript for authors to include a comment on the conservation between Ydj1p and DNAJA1. It appears that DNAJA1 has a weaker association with Abeta in the IP experiments.
11. In Fig 4I, please include Abeta only condition, as BSA can influence aggregation dynamics. Also, including DnaJ condition is

not particularly informative without analysis of conservation of DNAJ and DNAJA1. If DNAJA1 background is a concern, could the authors use a ThT assay at only two time points (t0 and t~1000s) and report the difference between the two timepoints (accounting for increased background in DNAJA1) rather than kinetic trace? Perhaps fibril sedimentation or filter trap or another assay could be used instead.
12. The authors do not directly address the contribution of cellular respiratory state on their phenotypes. They suggest that they are linked in multiple ways (Rho0 strains, using stationary phase timepoints for their yeast work). However, it would be really nice for the authors to tackle this head on, perhaps in the discussion.
13. For the drosophila work, mRNA level is used as a proxy for protein level. Could the authors share blots (with loading controls) to confirm that this is true?
14. There are clear sex differences observed in the fly experiments-could the authors comment on these?

MINOR POINTS:
15. Please include loading controls for 1F, S2 & S3 (many panels),otherwise the results cannot be interpreted.
16. Given that mitochondria localize throughout the cell, the imaging in S1F doesn't support the authors' statement that the EGFP+ puncta localize particularly to mitochondria.
17. It would be helpful to see the corresponding results for Abeta40 for S1L.
18. Ydj1p levels are not clear in overexpression strains. Could the authors somehow quantify this to compare to wild type levels?
19. It is important for the authors to distinguish the direct vs indirect nature of the some of the effects they attribute to Ydj1p. For example, when referring to Ydj1p mediating translocation to the mitochondria, the authors could instead use mitochondria-proximal localization. Also, please add the costained control to 3E.
20. Figures 3-5 are referred to incorrectly multiple times in the text. Please revise.
21. Panel 4G is a beautiful experiment-very clear.
22. The staining for 6E10 in panel 5A lacks other cellular markers - this panel could be greatly strengthened by the addition of other neuronal markers to show intracellular or extracellular staining of Abeta.
23. To support one of the authors' points in the discussion, clusterin and crystallin have also been shown to stabilize oligomeric Abeta (PMIDS: 22179788, PMID: 17412999, PMID: 23106396), and inhibit fibrillization (PMID: 20632140) though shown in these studies for Abeta40, not 42.

Referee #3 (Comments on Novelty/Model System for Author):

While the data are overall conclusive and experiments well controlled, the pathophysiological relevance of these findings for Alzheimer's disease are unclear given the artificial nature of the experimental systems used in this study. It would be important to validate the findings in a more appropriate model of AD

Referee #3 (Remarks for Author):

Ring et al. describe the identification of the HSP40 family member Ydj1 as a modulator of aggregation and cellular toxicity in yeast and fly models of Aβ42 related toxicity. Ydj1 was identified in a screen with yeast deletion mutants that rescue toxicity of an EGFP-Aβ42. Interaction of EGFP-Aβ42 and Ydj1 was confirmed by pulldown assays. Biochemical and cell biological experiments suggest that Ydj1 interferes with fibril formation of EGFP-Aβ42, but stabilizes oligomeric assemblies and promotes association of this fusion protein with mitochondria. Knockdown of the Drosophila Ydj1 homolog decreased mortality and reduced Aβ42 induced impairment of olfactory short term memory.
While the data are overall conclusive and experiments well controlled, the pathophysiological relevance of these findings for Alzheimer's disease are unclear given the artificial nature of the experimental systems used in this study. It would be important to validate the findings in a more appropriate model of AD.
Specific comments:
1. Authors used an EGFP-Aβ42 fusion construct to induce toxicity in yeast and show that EGFP-Aβ42 forms aggregates in the cytosol. Although intraneuronal Aβ is commonly detected in AD brains, these deposits appear to be mainly vesicular. It would be important to demonstrate Aβ peptides in the cytosol and the interaction with DnaJ with more appropriate neuronal model with either endogenous or transgenic expression of APP or addition of extracellular Aβ. The Aβ42 transgenic Drosophila model and Kar2-A42 yeast model also have limitations as there is very high expression of the peptide not derived from its precursor APP in the secretory pathway causing aggregation in the lumen of vesicular compartment and cell stress with unclear relation to the situation in vivo.
2. Authors identified an Aβ reactive band at 100 kDa and mentioned this could be a specific tetrameric assembly of EGFP-Aβ42 that preferentially associates mitochondria. It should be tested whether such a tetramer also associates with Ydj1/DnaJA1 in

above mentioned neuronal models and mouse or human brains.
3. The effect of Ydj1/DnaJA1 overexpression and deletion on Aβ induced toxicity and mitochondial activity should be tested in neurons. Primary cultures or iPSC models could be used.
4. Ydj1/DnaJA1 coimmunoprecipited with EGFP-Aβ42 monomers (Fig.3c). Does it also coprecipitate the tetramer?
DnaJA1 seems not to promote formation of a tetramer from synthetic Aβ42, but rather of higher oligomeric or fibrillar structures (Fig.4G). How does this relate to the tetramer found with the EGFP- Aβ42 construct? It would be important to characterise these structures (e.g. by TEM or AFM).
5. Authors mention that DnaJA1 interferes with ThT readings. Congo red could be used as an alternative compound to assess formation of β sheet aggregates.
Additional points.
- References to figures 3 and 4 are erroneously asigned in the text.
- Uathurs mention in the discussion „Nevertheless, Abeta can be produced by proteolysis of APP in intracellular space as well (Wilson et al., 1999), where it can escape the secretory pathway (Bückig et al., 2002) and be transferred from intracellular membranes (e.g. the ER) into the cytosol (Skovronsky et al., 1998; Wild-Bode et al., 1997)."
It is unclear what is meant with „proteolysis of APP in intracellular space"? The escape of Aβ from the secretory pathay is very controversial. As mentioned above it would be crucial to validate the present findings in a more appropriate neuronal model.

Point-by-Point Reply to the Reviewer's comments

Referee #1 (Comments on Novelty/Model System for Author):

Please note that all ***page and line*** *cross references refer to the final version of the manuscript (not to the track change file)*

The findings are novel but the medical impact is currently low to medium as more experiments are needed about testing memory in flies.

The primary aim of our study was to use genetically tractable model systems that allow mechanistic research, which we believe has strong potential for direct translational impact in follow up studies. Alzheimer's disease (AD) is still an untreatable disease. Due to beneficial effects of specific molecular chaperones in neurodegeneration, chaperone-based therapy is on a rise. Nevertheless, chaperone networks, which serve as guardian of cellular proteostasis, are profoundly complex and in certain conditions can act as a "double-edged sword" (Tittelmeier et al., 2020). We think that detailed evaluation of different heat shock proteins is fundamental in order to develop high precision therapeutic strategies, considering the low rate of success in the development of AD therapies. The biggest challenge on a way to find treatment or prevention of this disease is the decades-long phase before the development of severe AD pathology.

At this point, our research mainly relied on appropriate model systems. However, as suggested, we have further strengthened our data in the fly model, but we also now include observations from mammalian models and human samples as detailed below. Given the importance of this topic, we hope that the referee agrees with us on the potential medical impact of our paper.

Referee #1 (Remarks for Author):

In this study, the authors performed a series of elegant experiments in yeast and fly models to conclude that DNAJA1 may function as a driver of AB42 toxicity via mitochondrial translocation. The conclusion about cytotoxicity is supported by a convincing body of data. However, additional experiments are needed to firmly establish the importance of these findings for the regulation of behavior/memory.

Major concerns

A critical question raised is whether the role of DNAJA1 in driving the cellular AB cytotoxicity as inferred using yeast and flies might be important for memory and cognitive performance. This question is solely addressed in flies (Fig. 5J) and not in higher order models such as mouse models.

We agree with the referee in terms of model limitations used in this study. However, the fact that AD is exclusively a human disease (except for some other primate species) (Finch and Austad, 2015) none of the models can fully mirror events taking place in human brain tissue during the lifetime, but the models we used can help us dissecting conserved molecular mechanisms.

The lack of specific DnaJA1 inhibitors (see also response to the respective *Minor point* below) complicates the quick and easy implementation of an established AD mouse model into our research. Nonetheless, in the scope of this revision, we were able to utilize brain homogenates of 15 months old female mice from the 3xTg mouse model (Oddo et al., 2003) to investigate DnaJA1 binding properties towards Abeta from *ex vivo* extracts. We demonstrate that DnaJA1 co-precipitates with different Abeta oligomeric species as well as full length APP from enriched cytosolic fractions (Fig 5A of the revised manuscript).

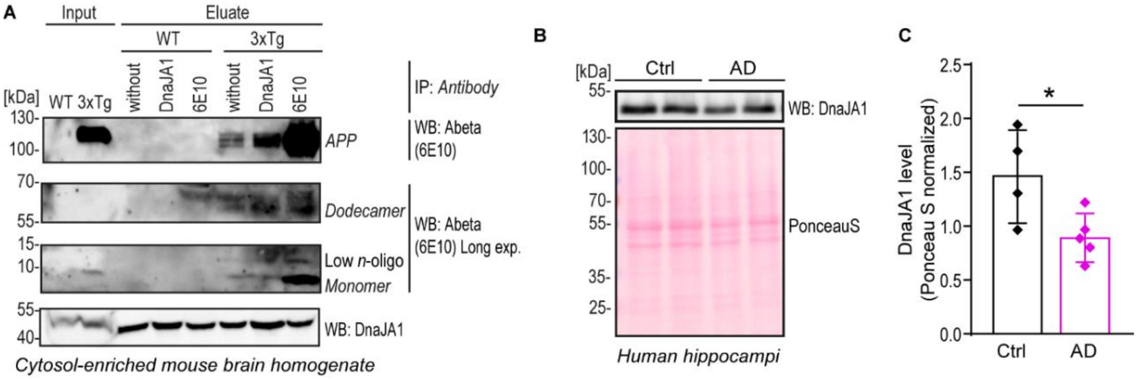

Figure 5

Importantly, in order to translate our findings to human relevance, we investigated DnaJA1 protein levels in post-mortem human hippocampi of AD patients. We could demonstrate that DnaJA1 is downregulated in AD post-mortem brain tissue (Fig 5B-C of the revised manuscript) and further discuss in the revised manuscript potential relations of this finding to our results from model systems (*Page 13, lines 14-26, see below)*. Moreover, DnaJA1 has been also recognized as a major 'pathogenic gene' (Tao et al., 2020), arguing for a DnaJA1's crucial role in AD, while the exact dynamics of DnaJA1 in aging and AD in humans is yet to be resolved.

Page 13, lines 14-26:

"Interestingly, DnaJA1 is downregulated in post-mortem brain samples of patients who suffered from AD (Abisambra et al., 2012; Sorrentino et al., 2017) and has been postulated as one of the major AD- and MCI-associated genes using bioinformatic meta-analysis (Tao et al., 2020). In a similar fashion to our results, this particular chaperone was upregulated as part of an early heat-shock response in spinocerebellar ataxia-7 (SCA7) -patient derived fibroblasts (Scholefield et al., 2014) and in young SCA7 transgenic mouse model, while it was downregulated in older SCA7 mice (Chou et al., 2010). Age-dependent regulation of DnaJA1 levels in cortical tissue has been already reported with highest expression in teenagers and young adults (16 – 23 years) (Breen et al., 2018). At the same time, oligomeric Abeta shows temporal profiles throughout aging, some being already present at early age long before first symptoms occur (Lesne, 2014). Accordingly, AD progression most likely starts at least two or three decades before actual diagnosis. These data together with our results suggest that DnaJA1 plays a crucial role in AD, but it remains yet to be determined at which stage of this malady DnaJA1 may exert its pathological actions."

In Fig 5J, the increase of memory performance in Droj2+/- UAS-A42 male flies compared to control flies is moderate (small amplitude with $p < 0.05$), suggesting that the role of DNAJA1 in driving AB42 cytotoxicity may have little impact on memory loss in AD. More experiments are needed to evaluate the importance of DNAJA1 for memory, particularly as the authors did not use mice. One possibility is to search for genetic enhancers of memory defects in Droj2+/- UAS-A42 flies, for example by using a candidate gene approach (e.g. a gene network-guided approach), to determine if such enhancers might lie in the HSP pathways.

We acknowledge candidate gene approach as an excellent idea. However, due to the extensive nature of this experimental approach (such as crossing additional fly lines), the time and working possibilities in the frame of this revision (also in light of restrictions we were facing during the pandemic) made this approach impossible and beyond the scope of this article. However, we will pursue this idea in following and ongoing projects.

We do agree that Droj2 knockdown induces rather moderate effects toward Abeta induced pathologies in the fly model. Nevertheless, one should keep in mind that the used Droj2 mutation line has only ~40 % of *Droj2* mRNA reduction (Fig EV4F). Thus, the modest effects that we observe are correlating to Droj2 levels in the system.

In order to further dissect Droj2 functions in the fly AD model, in scope of this revision, we performed additional experiments as suggested. We investigated Abeta burden in Droj2+/- and Droj2+/+ background by analyzing confocal images of fly brains stained with the Abeta-specific 6E10 antibody (Fig 6A-B, EV4A-B of the revised manuscript), revealing a decrease of Abeta burden upon Droj2 reduction in male, but not female flies (Fig 6C-D, EV4CD of the revised manuscript). In addition, we evaluated mitochondrial morphology by utilizing gSTED microscopy by ATP5A staining in Abeta expressing male flies, demonstrating that mitochondrial morphology phenotypes are more similar to the wild type control condition (without Abeta) upon Droj2 knockdown compared to flies with normal levels of Droj2 (Fig 7D-F, EV5D-H of the revised manuscript).

Another possibility is to enhance the sensitivity of the assay in flies, testing for the speed of odor choice (using video recording) in order to complement the current data about the result of odor choice. Lastly, it is unclear why the authors elected to not test for memory in female flies: why would the increased manganese stress resistance in female flies bearing Droj2 reduction prevent testing these flies for memory performance?

Thank you for the interesting suggestion to optimize our memory assay by additional video recording. We are indeed currently optimizing similar assays in our lab, by adding movement tracking to most behavioral assays. Nevertheless, we still did not succeed with this in our memory assay, due to technical difficulties.

We indeed agree with the suggestion to add female flies to our memory assay (Fig EV4G of the revised manuscript). Female flies, however, did not show memory protection upon Droj2

mutation, going in line with rather moderate and non-significant changes in Abeta burden in these flies (Fig EV4C-D of the revised manuscript).

This argues for sex specific Droj2 effects at least on memory performance in this model. Interestingly, we also observed sex-specific Droj2 responses to Abeta expression, with males having significantly higher Droj2 mRNA levels in AD flies compared to the wild type control (Fig 6F), whereas female flies only showed a trend (Fig 6E). Whether this represents a true sex-specificity or results from suboptimal knockdown efficiency of Droj2 in female flies remains to be studied.

<u>We added the following paragraphs in the text to account for this new data and to discuss potential sex-specificity:</u>

Page 9, lines 24-29

:"…To assess if reduced levels of Droj2 influence Abeta burden across the brain in mid-aged flies, we quantified the average intensity and total area of Abeta (6E10) signals using confocal images. Significant reduction in Abeta accumulation was detected in Droj2 knockdown male flies (Droj2+/- UAS-A42) (Fig 6C-D), albeit unchanged Abeta42 mRNA levels (Fig S1G). However, this effect was moderate to absent in female flies (Fig EV4C-D), indicating sex-specific effects.

Page 10, lines 1-6:

"Since Abeta42 expressing flies develop memory deficits (Iijima et al., 2008), we aimed to investigate whether Droj2 reduction can mitigate loss of memory in aged AD flies. Indeed, Droj2 reduction significantly improved short-term olfactory memory (STM) in aged (18-day-old) Abeta42-expressing male, but not female flies (Fig 7C, EV4G), correlating with Abeta burden in male and female flies and again, suggesting sex-specific effects in this model system (Fig 6C-D, EV4C-D). "

Page 12, lines 8-15:

"Using an AD fly model, we demonstrated phylogenetic conservation of our findings. Depletion of the Ydj1/DnaJA1 fly homologue, Droj2, improved mitochondrial morphology, diminished Abeta42-mediated toxicity upon manganese stress and partly reestablished Abeta42-induced memory loss in aged flies in a sex specific manner. Droj2 was significantly upregulated in response to Abeta42 expression in males, but to a lesser extent in female flies. Moreover, Droj2 knock down male flies accumulated less Abeta burden during aging compared to Droj2 proficient controls, once again an effect that was less pronounced or absent in females. One may speculate that the fine tuning of Hsp40 levels could be responsible for the observed sex differences, however future studies are needed to corroborate this. "

Additionally, there is no evidence for the effect on memory to be mediated by the expected target, that is lesser mitochondrial translocation in the brain of AB42 flies bearing Droj2 reduction compared to that of AB42 flies.

With the newly performed quantitative analysis of Abeta levels (Fig 6C-D, EV4C-D of the revised manuscript), we do have first evidence that the memory effect in male flies might come from lowered Abeta burden in mid aged flies upon knockdown of Droj2, which goes in hand with less signs of mitochondrial fragmentation/ morphological alterations (Fig. 7D-F of the revised manuscript). Future work has to test now, whether these alterations can be explained by changes in Abeta mitochondrial localization. We indeed made an effort during this revision already, to resolve Abeta localization in the fly model, presenting for the first time a high resolution gSTED Abeta imaging (Fig 6B, 7D, EV5B-C of the revised manuscript). We do observe mitochondrial contact sites (ATP5A staining) with Abeta (6E10 staining) signals (Fig EV5C of the revised manuscript).

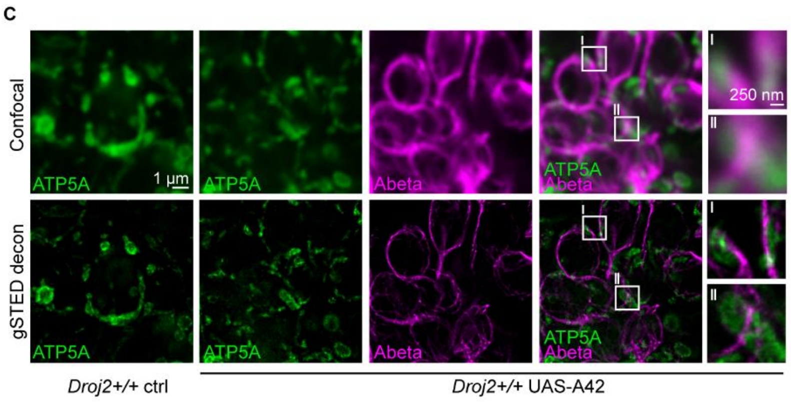

Expanded view 5

However, we are currently limited in quantification of distinct Abeta distribution between Droj2 (+/-) and Droj2 (+/+) lines, because of the present predominant ER localization of the Abeta signal (Fig EV5A of the revised manuscript). This fact currently prohibits discrimination between Mitochondria-ER contact sites versus Abeta to mitochondria translocation events. Therefore, further optimization (e.g. by developing a multi-color gSTED protocol including ER co-staining) of this method is planned in future studies.

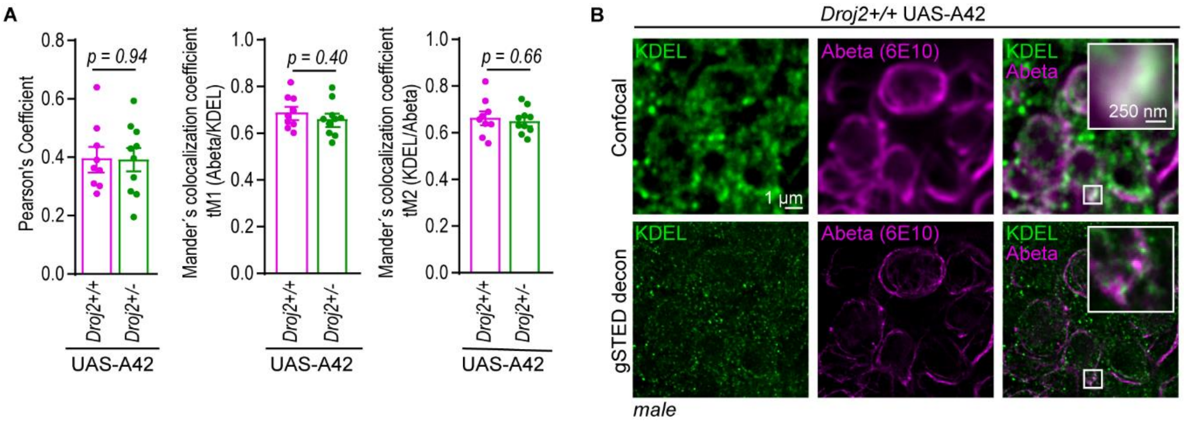

Expanded view 5

The discussion on the unexpected role of DNAJA1 in driving AB cytotoxicity compared to that of HPSs in opposing cytotoxicity needs to be expanded, discussing this finding in the context of the knowledge on the role of Tau, notably phospo Tau, compared to that of AB42 in driving AD, also discussing this finding in the context of other well-studied neurodegenerative diseases such as for example Huntington's disease.

We truly agree with the reviewer that the discussion of distinct DnaJA1 functions in different neurodegenerative diseases needs to be expanded, thus we extended our discussion regarding this topic.

Page 12, lines 32-40:

"…. In parallel, another study tested HSP40 effects on the AD-associated protein tau showing that over-expression of DnaJA1 can favor both tau clearance and stabilization dependent on Hsp70 levels in M17 neuroblastoma cells (Abisambra et al., 2012). A recent study reported similar findings, whereby inducing DnaJA1 activity by CRBN (endogenous substrate of cerebelon) downregulation, decreased phosphorylation and aggregation of tau was detected in vivo and in vitro (Akber et al., 2021). Furthermore, Abisambra et al. also demonstrated DnaJA1-induced polyQ clearance, while alpha-synuclein stability was unaffected in a model of Parkinson's disease. This goes in line with our observation that YDJ1 does not influence alpha-synuclein toxicity in yeast, …"

Minor points

It is a missed opportunity that the authors did not test for the effects of chemical DNAJA1 inhibitors in flies (unless there are some issues with the access to or the specificity and use of these compounds), as this would provide a basis for similar testing in mouse models of AD and other neurodegenerative diseases.

We thank the reviewer for this idea, since utilization of a HSP40-specific inhibitor would allow further translational studies in other model systems (including mice).

Unfortunately, we were not able to find specific DnaJA1 or HSP40 chemical inhibitors. Nevertheless, we utilized the commercially available chemical inhibitor 116-9e, which targets the Hsp70/Hsp40 interaction, already tested in a yeast model (Wisén et al., 2010). 116-9e acts via binding to Hsp70, thus inhibits the formation of Hsp40/Hsp70 complex resulting in a reduction of (Hsp40-activated) Hsp70 ATPase activity.

Applying this inhibitor to yeast cells expressing EGFP-A42, we did not observe rescuing effects on cell death (Fig EV2F-G of the revised manuscript). This argues for an Hsp70-independent function of Hsp40 towards Abeta toxicity. Therefore, the inhibitor 116-9e appears not suitable for use in higher models in order to mimicry DnaJA1 disruption.

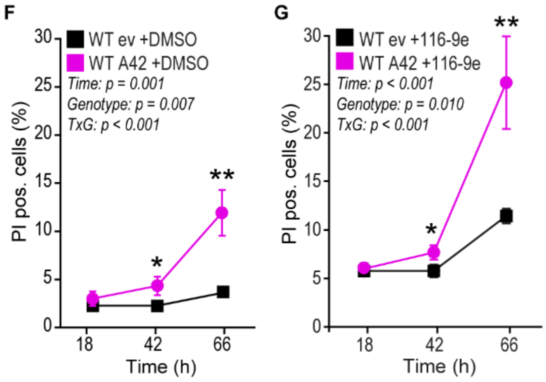

Expanded view 2

<u>We added the following paragraph in the text to account for this new data</u>:

Page 6, lines 14-19:

Ydj1 has been reported to act in part through interaction with Hsp70, activating Hsp70's ATPase activity. Therefore, in an attempt to mimic the effects of YDJ1 deletion, we used the Hsp40-Hsp70 interaction inhibitor 116-9e (Wisén et al., 2010). Treatment with 116-9e did not lower but rather increased cell death in wild type cells expressing EGFP-42 compared to vehicle (DMSO) treated controls (Fig EV2F-G), arguing for an Hsp70-independent function of Ydj1 to be crucial for Abeta42 toxicity.

Referee #2 (Comments on Novelty/Model System for Author):

The choice of yeast model--an episomal plasmid expression system--is not very well justified and adequate controls are not included to allow the reader to interpret the data. These have been detailed in my remarks to authors below.

Thank you for your comment. In addition to our replies to the specific points below, the choice of specific expression system is justified by following reasons:

Initially, in order to establish an AD yeast model mimicking intracellular Abeta toxicity, different expression constructs were tested. Out of the tested constructs, yeast cells expressing Abeta42 linked to an N-terminal EGFP tag with a linker sequence in between, yielded best results in terms

of producing cytotoxicity. Importantly, cell death induced by this construct was only achieved with an episomal plasmid system containing a strong inducible GAL4 promotor. Therefore, we chose to use this system for further studying pathological pathways behind this cytotoxicity. Nevertheless, we agree with the referee that a centromeric expression system may have advantages above episomal plasmid-driven expression and therefore did generate a centromeric plasmid carrying our specific EGFP-Abeta fusion construct (including also the GAL4 promotor) and added the results in this rebuttal letter (*see below*). Surprisingly, the centromeric plasmid did not result in a more homogenous expression among cells of a given culture and also lacked pronounced toxicity, justifying the need for an episomal high-copy number system.

Referee #2 (Remarks for Author):

*Please note that all **page and line** cross references refer to the final version of the manuscript (not to the track change file)*

This paper describes work in two model systems to show that the presence of mitochondrial HSP40 chaperone, Ydj1p in yeast and DNAJA1 in humans, exacerbates the detrimental effects of amyloid-beta42, through the modulation of its aggregation state. I appreciate the use of multiple model systems to illustrate the findings in this work. This finding is potentially interesting, especially with the connection to oligomerization. However, the work could be strengthened by adding multile controls that would allow the reader to interpret the data and substantiate the authors' claims.

MAJOR POINTS

1. It appears that yeast models in this paper rely on episomal plasmids. These are inherently variable in copy number and strains expressing toxic proteins can select against high copy number clones. This makes many of the results throughout this paper difficult to interpret. That difficulty is compounded by the lack of loading controls throughout multiple western blots of amyloid protein levels and aggregation extent. The authors should justify this choice of using episomal plasmids or perform a few key experiments with copy number-controlled, integrated or centromeric strains to show a true peptide/sequence-specific effects with accompanying western blots.

We fully concur with the referee that episomal plasmids may not be ideal in all cases. However, when using centromeric plasmid used for cytosolic Abeta expression, it appears that yeast cells are even more capable of adapting to or escaping Abeta toxicity: the centromeric plasmid p416 carrying Kar2-A42 (Chen and Petranovic, 2015) was tested in our yeast strain (BY4741) and culturing conditions on cell stress (DHE staining) and analyzed using immunoblots (Point-by-Point Fig IA-B). We were not able to see Abeta42 induced cytotoxic phenotypes in our conditions. Further, we have now cloned our specific EGFP-A42 construct including the galactose promotor into the centromeric plasmid p416. We compared wild type cells expressing EGFP vector control (ev) with EGFP-A42 expressing cells (Point-by-Point Fig IC-E). Surprisingly, only a minor fraction of cells showed clear GFP positivity and cultures appeared even more heterogenous compared to our episomal system (Point-by-Point Fig IC). Again, we did not observe an increase in cell death

(PI positive cells) when expressing EGFP-A42 by the p416 centromeric plasmid (Point-by-Point Fig ID). We conclude that a quick and strong expression of Abeta42 may be necessary to counteract its fast degradation and allow toxicity, which we achieve using the episomal pESC system.

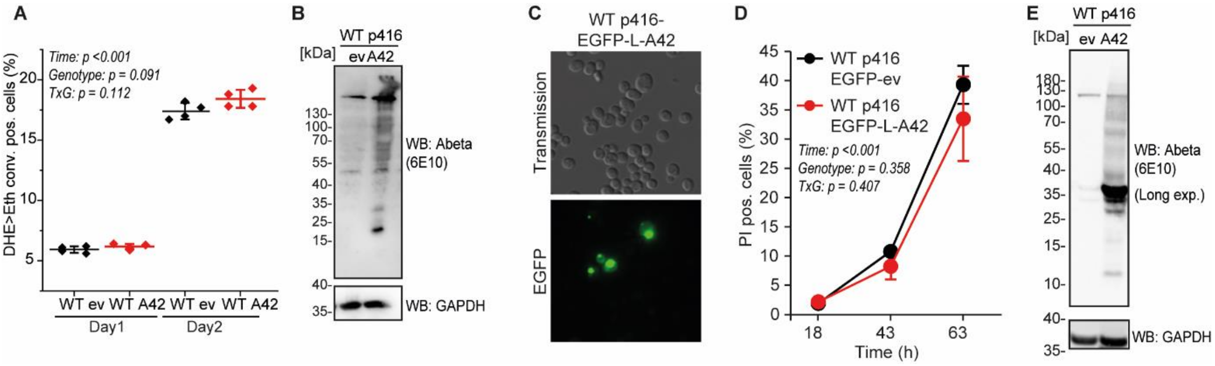

Point-by-Point Fig I

We thank the reviewer for pointing out the missing loading controls. We have now added all corresponding loading controls (Fig 1F, EV1B (former S1F), EV2C (former S2L), EV3A-E (former S2A-D, S4A).

2. The DHE metric for cell death/oxidative stress is relevant, but for comparison to past yeast literature, it would be helpful to provide standard growth curves (OD600) or spotting assays for all lines used throughout the paper. This would allow readers to be able to process all these finding in the context of other yeast neurodegeneration literature.

In order to evaluate EGFP-A42 influence on yeast growth, we assessed growth curves of wild type cells expressing A42 and corresponding empty vector expressing EGFP only. We did not observe any effect on yeast growth monitoring OD_{600} using the automated Bioscreen C MBR system for 48 hours after shift to galactose containing media. We have included the growth curve to the revised manuscript (Fig S1A of the revised manuscript). Notably, assessing cell death can be a more sensitive method in certain conditions and it was our initial aim to evaluate Abeta cytotoxicity with a true cell death phenotype, since AD is characterized by neuronal cell death as one important hallmark.

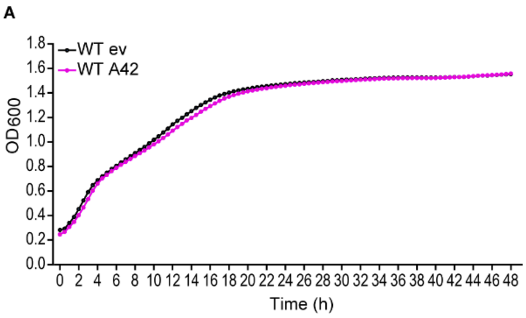

Figure S1

<u>We added the following paragraph in the text to account for this new data:</u>

Page 4, lines 6-9:

Expression of EGFP-A42 did not impair growth (Fig S1A), but led to an increase in the fraction of dead cells or cells exhibiting oxidative stress, as assessed by the number of dihydroethidium to ethidium (DHE>Eth.) positive cells compared to all corresponding non-toxic controls (ev, EGFP-C57 and EGFP-A42m2) (Fig 1B-D).

3. The authors have a very powerful set of controls in C57, Abeta40, and Abeta42m2. However, they could use them more broadly to prove their points about Abeta42. For example, given that Abeta40 is similarly toxic to Abeta42 in their model (S1A), but doesn't have the tetrameric form that the authors suggest is important for Ydj1p activity (1F), it would be a good control to decouple oligomerization from toxicity. Also, the other forms C57, and Abeta42m2 could be used to explore effects of non-toxic cytosolic peptide expression (for example, localization etc...). Again, this is best done in a copy-number- or expression-controlled setting.

We think that this is an important concept that the reviewer points out here. Indeed, we used the different forms/mutants of Abeta peptide sequences to control our Abeta42 yeast expression model, with regard to cell stress (Fig1B-D), their cellular localization (Fig 2A), induction of Ydj1 expression (Fig EV1F) and their oligomerization properties (Fig1F-G).

To complete this as suggested, we performed an additional assay evaluating the localization of the non-toxic C57 peptide control (Fig EV1A of the revised manuscript). We observe rather cytosolic distribution of EGFP-C57, in comparison to the primarily mitochondrial localization of Abeta42 and Abeta40 peptides (Fig EV1A). This goes in line with cell stress data showing no toxicity of C57 (Fig 1C). Further, even though interacting with Ydj1 (Fig EV2H), C57 did not lead to Ydj1 induction response (Fig EV1F). Consistently, the mutated form of Abeta42 (m2) has been found mostly in the cytosol (Fig 2A), promoting no cellular stress (Fig 1D) and no induction of Ydj1 levels (Fig EV1F), including no Ydj1 interaction (Fig EV2I).

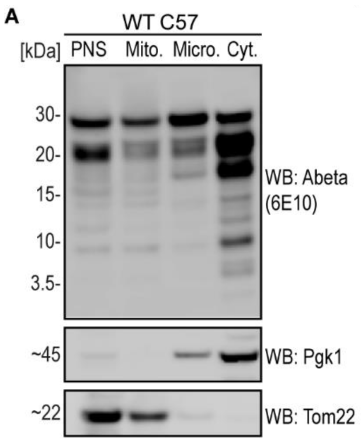

Expanded view 1

It has been shown by others, that Abeta40 might be less toxic (Chen et al., 2021; Dahlgren et al., 2002), possibly due to different oligomerization properties (Bitan et al., 2003), which we also observed in our experiments (Fig 1F, 2A). Nevertheless, our present experiments were not powered enough to detect small differences in toxicity (Fig 1B, *p-value* = 0.121 comparing A42 and A40) and therefore many more experiments would be needed to evaluate these potential differences, which we decided was beyond the scope of this study. However, we discuss now more carefully that Ydj1/DnaJA1 affects several oligomeric forms, including the tetramer.

4. There have been reports of multimerization of EGFP (also evidenced in Fig S1D)-it would greatly improve the claims of multimerization if a monomeric version of GFP were used like monomeric superfolder GFP, for example. It would be more convincing if the authors could repeat a few of the imaging experiments and multimerization experiments either with antibody ICC (on an untagged strain) or another known monomeric fluorescent tag. This would suppress doubts that the EGFP tag, which far exceeds the size of the abeta peptide itself, is modifying this behavior or enhancing aggregation potential.

We share the opinion with the reviewer, thus we aimed to validate our findings using different AD models. In this study, we utilized the Kar2-A42 expressing yeast strain to study Abeta oligomerization, Abeta-dependent Ydj1 interactions and Abeta-induced cell death. Using this strain, we could show that Abeta can be cytotoxic also without an EGFP fusion and validate Ydj1's role in Abeta oligomerization and toxicity in this cellular system (*see also below, response to point 5*).

In addition, to ensure that the observed Abeta effects are independent of the EGFP tag itself, we have cloned the same construct using monomeric mGFP, containing a point mutation (A206K) preventing self-dimerization (Zacharias et al., 2002). Using this construct, we repeated a set of experiments including immunoblotting and cell death (PI positive cells) assay, validating Abeta-mediated cell death induction (Point-by-Point Reply Fig IIA) and oligomerization properties (Point-by-Point Reply Fig IIB).

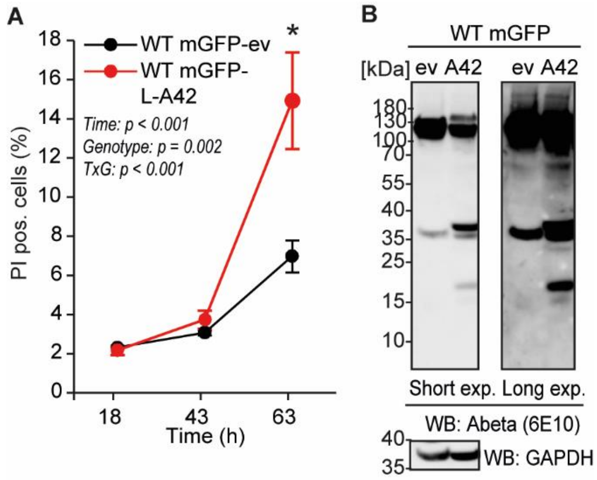

Point-by-Point Reply Fig II

5. Although many of the findings in this paper are interesting, it is hard to know how many of the observations result from cytosolic expression. The Kar2-Abeta yeast model that the authors include in some selective experiments could better reflect the more physiological context for Abeta - in the secretory pathway (still intracellular)-rather than cytosolic. And differences between the Kar2-abeta and the cytosolic abeta strain can give insight into what effects are specific to the secretory pathway and which are specific to cytosolic expression. Therefore, I suggest the authors include the comparison for the Kar2-Abeta strain throughout the paper in multiple experiments to bolster their points. This will also complement well the results from the Drosophila model as it also uses a secretory-pathway directed Abeta construct.

We agree with the reviewer, since we think that these additional experiments strengthen our observations in the yeast model, thus we have added crucial experiments using Kar2-A42 construct (now collectively shown in Fig EV2A-E of the revised manuscript). In summary, both A42 constructs (EGFP-A42 and Kar2-A42) cause stress in wild type cells leading to cell death after 66 hours of expression and in both systems deletion of *YDJ1* rescues the phenotype (Fig EV2A-B of the revised manuscript). Both EGFP-A42 and Kar2-A42 lead to the presence of immunoblot detectable oligomers (Fig EV2C of the revised manuscript), which in both systems are shifted towards low *n*-oligomers in Ydj1 dependent manner (Fig EV2D of the revised manuscript). Last but not least, similar to direct cytosolic expression of EGFP-A42, a portion of Abeta was detected associated to mitochondrial cell fractions in spite of Kar2 sequence directing Abeta to secretory pathway (Fig EV2D of the revised manuscript). Thus overall, our results seem relevant for intracellular Abeta independent of the initial route of expression.

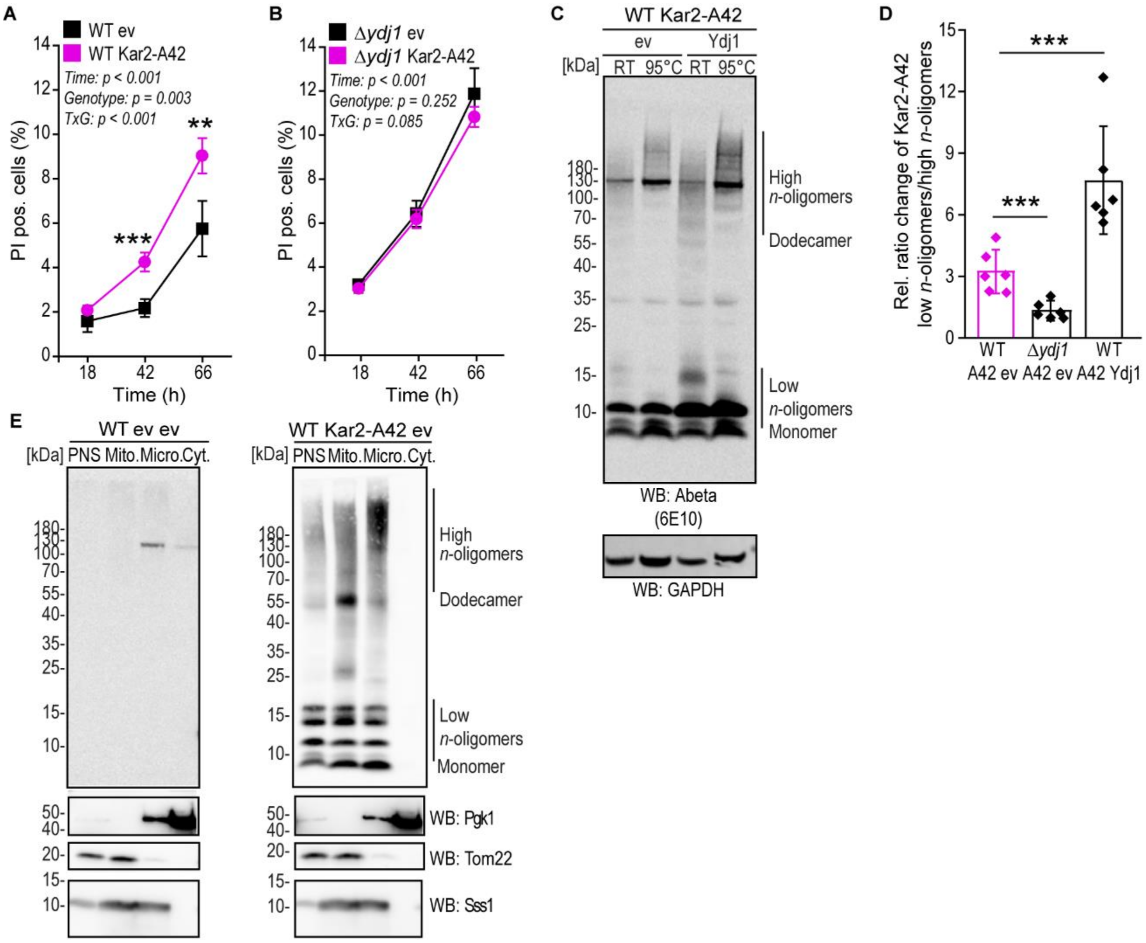

Expanded view 2

<u>We added the following paragraph in the text to account for this new data:</u>

Page 6, lines 21-23:

Importantly, deletion of YDJ1 also abolished Abeta42 induced cell death in an alternative yeast model where Abeta42 was fused to the Kar2 localization sequence, which directs the Abeta42 peptide to the secretory pathway (Treusch et al., 2011) (Fig EV2A-B).

Page 7, lines 29-36:

The Abeta42 stabilizing effect of Ydj1 could be confirmed in Kar2-A42 expressing yeast cells, again showing stabilization of low-n oligomers (Fig EV2C, D). Inspection of Abeta42 localization by cellular fractionation in this model revealed Abeta42 deposition in both mitochondrial and microsomal fraction, which can be explained by the initial Kar2-driven expression of Abeta42 towards the ER/secretory pathway. Importantly, the oligomer distribution differed between mitochondrial- and microsomal-enriched fraction in spite of a moderate microsomal contamination in mitochondrial fractions (Fig EV2E). Evidently, high-n oligomers were enriched in microsomal fraction, whereas the dodecamer and some low-n oligomers were rather detected in mitochondrial fraction.

6. There's an interesting difference between the time-dependence of Abeta42 expression on PI-positive cells (3A,B) compared to the ydj1-deletion strains. It looks like at 42h, ydj1 exacerbates the toxic abeta phenotype, whereas it may suppress at 66h. The ydj1 deletion seems to do this for the WT background too. Interestingly another interesting dynamic change in toxicity suppression can be observed in the Rho0 strains (S1G). Could the authors comment on all of these dynamics? Perhaps how they relate to respiratory state of the cells?

Ydj1 executes different functions in the cell, thus its absence may cause this intriguing aging/survival dynamics. Since many different strains have specific survival patterns in our conditions, we always compared Abeta expression strains directly to the respective background strain (empty vector control of a given mutant). The most important outcome is that the *YDJ1* deletion rescues Abeta induced toxicity irrespective of temporal changes in survival phenotypes. In addition, in our genetic screen, we observed different survival behaviors of different strains. Some of the strains that *per se* showed higher DHE positivity than wild type cells, still exhibited higher Abeta toxicity, indicating that a higher stress rate does not principally mask Abeta induced cell death.

As the reviewer speculates, some of these effects might partially depend on the respiratory state of *YDJ1* deletion strain. Even though this might be an appealing mechanism to investigate, we did not focus on this part, since we have confirmed that the *YDJ1* deletion strain is not respiratory deficient. This strain can grow on glycerol plates and thus differs from our findings obtained with rho0 strains or other respiratory deficient strains. We decided to focus on respiratory aspects in more detail in following work based on this project. However, following this important comment we have now included an extended part to this topic in our discussion:

Page 11 lines 7-10:

"…*Besides, we obtained evidence that Abeta42 triggers oxidative stress leading to cell death only if mitochondria are respiration-competent. This goes in line with previous findings that the key enzymes mediating the Warburg effect play a central role in mediating neuronal resistance to Abeta by decreasing mitochondrial activity (Newington et al., 2011, 2012)*…."

7. Given that ydj1 deletion seems toxic by itself (3A,B, S1J,K), how does one deconvolute these effects of toxicity additivity?

As outlined above in response to point 5, we think the most important outcome is that the *YDJ1* deletion rescues Abeta induced toxicity irrespective of temporal changes in survival phenotypes (Fig. 3A-B, EV2A-B of the revised manuscript). In addition, in our genetic screen, we observed different survival behaviors of different strains, of which some showed higher DHE positivity than wild type cells, but still exhibited Abeta toxicity, in clear contrast to the findings after *YDJ1* deletion. See 3 examples (NUC, SIR2 and ECM10) in Point-by-Point Figure III.

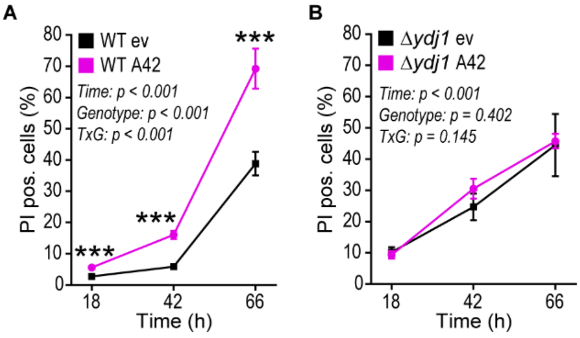

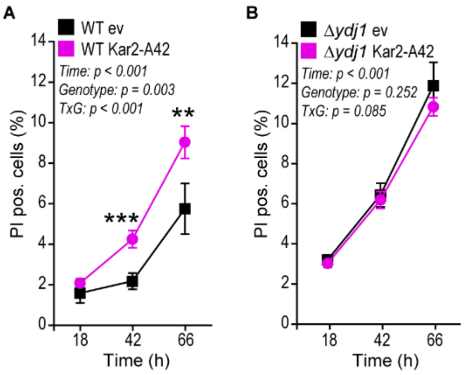

Figure 3 **Expanded view 4**

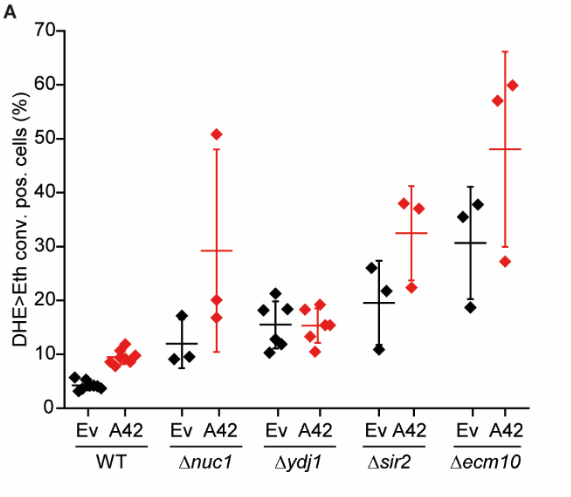

Point-by-Point Figure III

We agree that the effects observed in the *YDJ1* deletion strain have to be controlled, thus we performed Ydj1 overexpression experiments, which clearly show that Ydj1 increases Abeta mitochondrial localization (Fig 3G-H), as well as Abeta stabilization (Fig 3I) and oligomerization (Fig 3J-K). Additional support of Ydj1 being a *bona fide* modulator of Abeta toxicity is the fact that it interacts with Abeta (Fig 3C).

8. A missing strain from panels 3F & I is the YDJ1 overexpression in the WT background. It would be great if the authors could add this and the quantification of the blot. It seems like the authors have performed this type of experiment in some contexts (3H), but even for this context, the blot is only in S5 and is tiny without lane labels to allow for easy interpretation.

We apologize for the misleading of the data. Yes, indeed we have done cell fraction with the Ydj1 overexpression in the wild type background and also used it for quantification of the Abeta distribution (mito/cytosol ratio). We have now added the representative cell fractionation of WT cells expressing EGFP-A42 and the corresponding strain co-overexpression Ydj1 with all labels and in a reasonable size to supplemental figure S1 (Fig S1C of the revised manuscript).

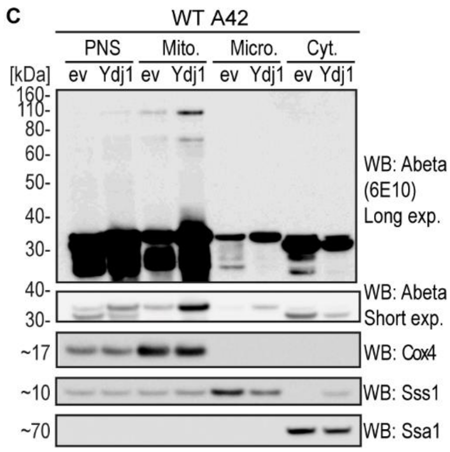

Figure S1

9. Sis1 conspicuously absent-Sis1 belongs to different class and suppresses toxicity of TDP-43 (Liebman lab, PLoS Genetics, 2017); would be interesting to know its effect here.

Since Δ*sis1* is not viable as a haploid deletion strain, it was initially not part of our knockout screen and was therefore not evaluated in this study. Important to mention is that both Ydj1 and Sis1 belong to the major HSP40 chaperone family in yeast. Ydj1 is an HSP40 class I and is the homologue of human DnaJA, whereas Sis1 belongs to the HSP40 class II subgroup, which is the homologue of the human DnaJB (Park et al., 2018; Stein et al., 2014). Strikingly, DnaJB6 has been already implicated in Alzheimer´s disease (Månsson et al., 2014; Österlund et al., 2020). However, it seems that DnaJB6 exhibits an opposite function in comparison to DnaJA1 in regard of polyglutamine aggregation (Rodríguez-González et al., 2020) and behaves differently towards yeast prion propagation (Barbitoff et al., 2020). Nevertheless, due to already comprehensive data load in this manuscript, we did not perform an additional evaluation of this protein, for which we do agree to be important in relation to Abeta aggregation, but discuss a possible role of DnaJB in the discussion part of our manuscript:

Page 12, line 41; page 12-13, lines 41-5:

"…Vice versa, divergent functions of Hsp40 chaperones have been reported. For instance, opposite actions of DnaJA1 and DnaJB6, another Hsp40 family member were demonstrated in an in vitro model of Huntington´s disease (Rodríguez-González et al., 2020). Interestingly, DnaJB6 has shown to inhibit the primary nucleation of Abeta40 oligomer formation and inhibits fibril formation of Abeta42 (Månsson et al., 2014; Österlund et al., 2020), It will be interesting to study commonalities and potential differences of DnaJA1 and DnaJB6 regarding their role in Abeta toxicity."

10. It would help the manuscript for authors to include a comment on the conservation between Ydj1p and DNAJA1. It appears that DNAJA1 has a weaker association with Abeta in the IP experiments.

We agree with the reviewer's comment, thus, we now included the citation of the study which evaluated human homologues of Ydj1 in yeast (Whitmore et al., 2020).

Nevertheless, we do not know if DnaJA1 completely complements all Ydj1 functions in a *YDJ1* deletion strain. In the pull-down assay we had shown (old Fig 4C), the expression of Ydj1-Flag in yeast seems to be stronger, compared to DnaJA1-Flag. Thus, we cannot clearly distinguish if DnaJA1 has a weaker association with Abeta in this pull-down assay. In order to investigate this possibility, more assays would be necessary. As this immunoblot of the immunoprecipitation experiment may be misleading to the readers, we changed this blot showing only the outcome of DnaJA1-Abeta interaction (Fig 4C of the revised manuscript). This should avoid direct comparisons of the two homologous proteins, which we did not address explicitly and need to be studied in the future by more appropriate methods. Please note, that the results of Ydj1 interaction to Abeta of course remain in the manuscript as they have been shown already before in figure (Fig 3C).

Fig 4C OLD

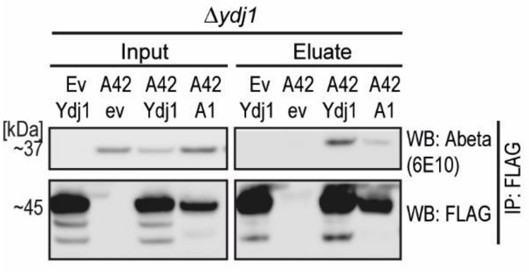

Fig 4C NEW

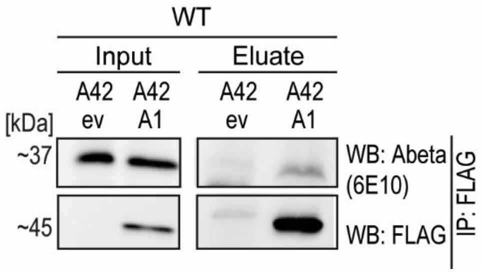

C

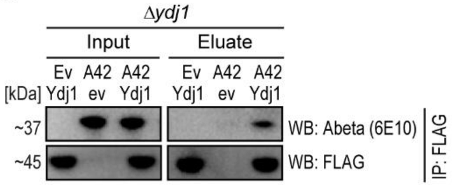

Figure 3

11. In Fig 4I, please include Abeta only condition, as BSA can influence aggregation dynamics. Also, including DnaJ condition is not particularly informative without analysis of conservation of DNAJ and DNAJA1. If DNAJA1 background is a concern, could the authors use a ThT assay at only two time points (t0 and t~1000s) and report the difference between the two timepoints (accounting for increased background in DNAJA1) rather than kinetic trace? Perhaps fibril sedimentation or filter trap or another assay could be used instead.

This of course can be a valid point and please find below the graph including the requested control without BSA (shown here in the Point-by-Point Reply Fig IVA). Importantly, even though BSA similar to DnaJ affected maximal ThT intensities, there has been no difference observed regarding the lag phase between BSA and the control condition lacking BSA. In contrast (as has

been shown in our initial manuscript already), DnaJ specifically delayed ThT fluorescence increase. We therefore conclude that BSA is the more appropriate protein-containing control and decided to show only the two comparable curves (Fig 4I). Of note, we do not know why lack of additional protein (BSA or DnaJ) reaches an overall lower ThT signal and think adding the non BSA control may rather confuse readers. However, if the reviewer believes that this is important for clarity and transparency, would of course agree to include this.

Regarding DnaJA1, we have already tried to use DnaJA1 in the ThT assay, nevertheless, this protein interacted strongly with ThT creating background problems as the reviewer correctly noticed. For transparency we included the respective graph now in the revised manuscript (Fig S1F of the revised manuscript). Moreover, we share the opinion with the reviewer regarding performing an additional assay (independent of ThT) to substantiate DnaJA1 effects towards Abeta aggregation properties. Therefore, we performed western blotting analysis of synthetic Abeta aggregation kinetics, evaluating specific oligomeric formation over time with and without presence of DnaJA1 (Fig 4G-H).

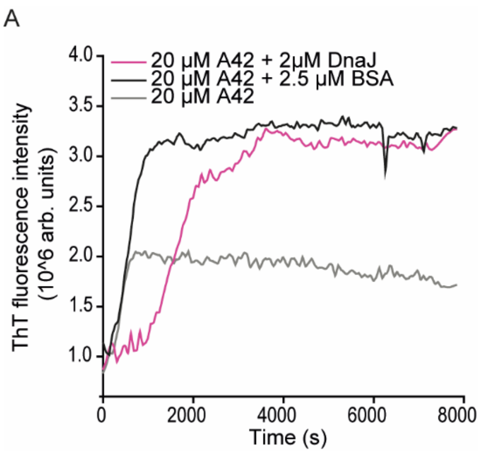

Figure IV

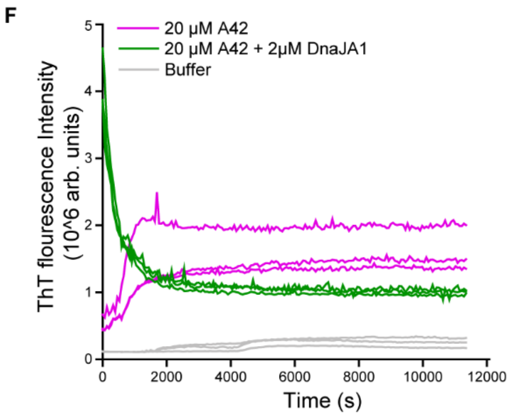

Figure S1

12. The authors do not directly address the contribution of cellular respiratory state on their phenotypes. They suggest that they are linked in multiple ways (Rho0 strains, using stationary phase timepoints for their yeast work). However, it would be really nice for the authors to tackle this head on, perhaps in the discussion.

Thank you for this idea and as the reviewer suggested we now shortly tackle the potential importance of respiration or the respiratory state of cells in AD and in light of our results in the discussion paragraph.

<u>We added the following paragraph in the text:</u>

(page 11, lines 7-10)

"…*Besides, we obtained evidence that Abeta42 triggers oxidative stress leading to cell death only if mitochondria are respiration-competent. This goes in line with previous findings that the key enzymes mediating the Warburg effect play a central role in mediating neuronal resistance to Abeta by decreasing mitochondrial activity (Newington et al., 2011, 2012)*…."

13. For the drosophila work, mRNA level is used as a proxy for protein level. Could the authors share blots (with loading controls) to confirm that this is true?

We performed these blot analyses confirming the mRNA results. They are now included in Fig EV4E of the revised manuscript.

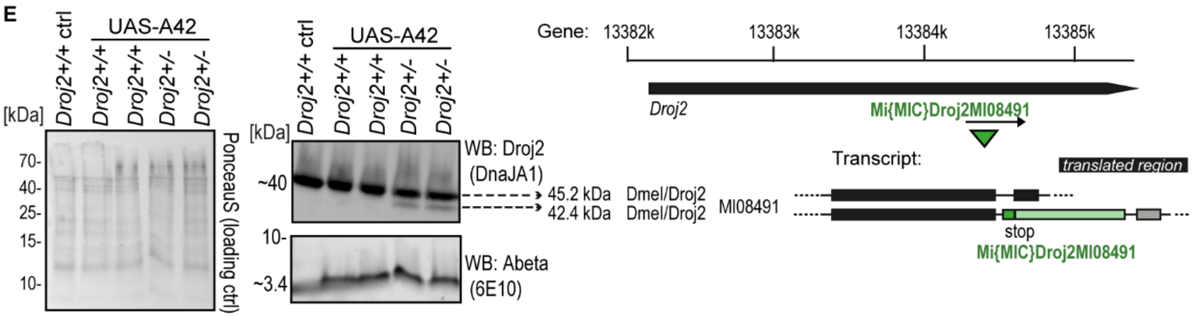

Expanded view 4

14. There are clear sex differences observed in the fly experiments-could the authors comment on these?

Undoubtedly, there are clear sex differences in performed fly experiments. Now, we extended our analysis and added new data underlying the sex-differences regarding the degree of Abeta-associated phenotypes reversed by knockdown of *Droj2*. We observed less Abeta burden in mid-aged male flies upon Droj2 downregulation (Fig 6C-D of the revised manuscript), with moderate or absent effects in female flies (Fig EV4C-D of the revised manuscript). Of note, Abeta mRNA levels in young flies were not significantly different between control and Droj2 knockdown flies expressing Abeta (Fig S1G). This observation goes in line with observed increased memory performance in male flies (Fig 7C), but not in female flies upon Droj2 reduction (Fig EV4G of the revised manuscript). Furthermore, we observed differential Droj2 response in a sex-dependent manner, with males having significant higher Droj2 upregulation (Fig 6E), and females exhibiting only a trend (Fig 6F). Nevertheless, both males and females Abeta expressing flies in Droj2 knockdown background showed rescuing effects upon manganese stress. Why this sex differences occur remains to be studied. We know that AD is sex specific disease (Paranjpe et al., 2021), with females having higher prevalence of getting this disease. We only can speculate that the fine tuning of DnaJA1 levels could be responsible for the observed differences, however future studies are needed to confront this issue. Notably, a study evaluating sex specific differences in AD, reported downregulation of DnajA1 in woman suffering from AD in whole blood, but this was not observed in man (Paranjpe et al., 2021).

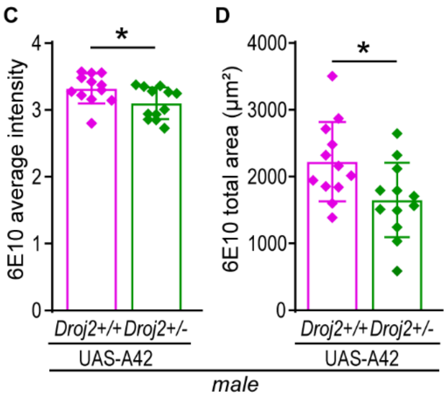

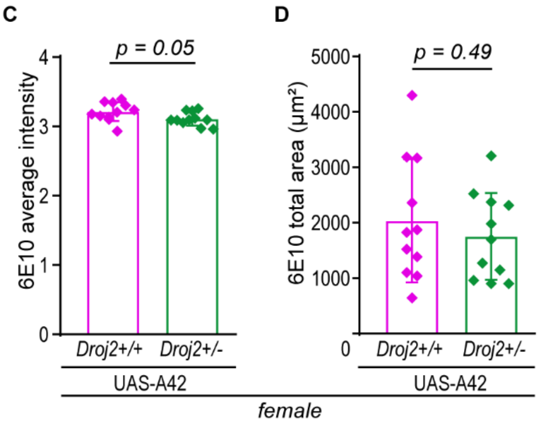

Figure 6 **Expanded view 4**

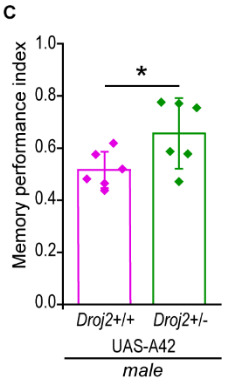

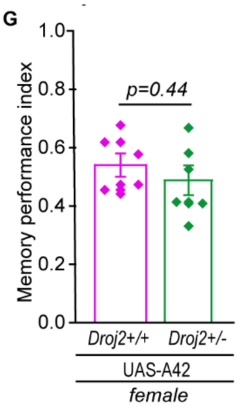

Figure 7 **Expanded view 4**

<u>We adapted the discussion at following points to account for this new data and to briefly discuss sex-specificity:</u>

Page 9, lines 26-29:

"Significant reduction in Abeta accumulation was detected in Droj2 knockdown male flies (Droj2+/- UAS-A42) (Fig 6C-D), albeit unchanged Abeta42 mRNA levels (Fig S1G). However, this effect was moderate to absent in female flies (Fig EV4C-D), indicating sex-specific effects."

Page 10, lines 1-6:

"Since Abeta42 expressing flies develop memory deficits (Iijima et al., 2008), we aimed to investigate whether Droj2 reduction can mitigate loss of memory in aged AD flies. Indeed, Droj2 reduction significantly improved short-term olfactory memory (STM) in aged (18-day-old) Abeta42-expressing male, but not female flies (Fig 7C, EV4G), correlating with Abeta burden in male and female flies and again, suggesting sex-specific effects in this model system (Fig 6C-D, EV4C-D)."

Page 12, lines 8-15:

"Using an AD fly model, we demonstrated phylogenetic conservation of our findings. Depletion of the Ydj1/DnaJA1 fly homologue, Droj2, improved mitochondrial morphology, diminished Abeta42-mediated toxicity upon manganese stress and partly reestablished Abeta42-induced memory loss in aged flies in a sex specific manner. Droj2 was significantly upregulated in response to Abeta42 expression in males, but to a lesser extent in female flies. Moreover, Droj2 knock down male flies accumulated less Abeta burden during aging compared to Droj2 proficient controls, once again an effect that was less pronounced or absent in females. One may speculate that the fine tuning of Hsp40 levels could be responsible for the observed sex differences, however future studies are needed to corroborate this."

MINOR POINTS:

15. Please include loading controls for 1F, S2 & S3 (many panels),otherwise the results cannot be interpreted.

We thank the reviewer for pointing this out and now include the loading controls to the revised manuscript (Fig 1F, EV1E (former S1F), EV2C (former S2L), EV3A-E (former S2A-D, S4A), EV4E (former S4C).

16. Given that mitochondria localize throughout the cell, the imaging in S1F doesn't support the authors' statement that the EGFP+ puncta localize particularly to mitochondria.

Localization of EGFP-A42 was analyzed by microscopy (Fig 3E, EV1C, EV2J), cellular fractionation (Fig 2A, 3F, EV2E, S1C), and quantification of mitochondria/cytosol ratio using Abeta-specific immunoblots after cellular fractionation (Fig 3G-H), showing indeed a partial rather than complete mitochondrial localization in all assays. To avoid misleading data, we have now added more confocal images (Fig EV1C of the revised manuscript) of yeast wild type cells expressing EGFP-A42 co-stained with MitoTracker Red for mitochondrial staining confirming partial co-localization of Abeta and mitochondria.

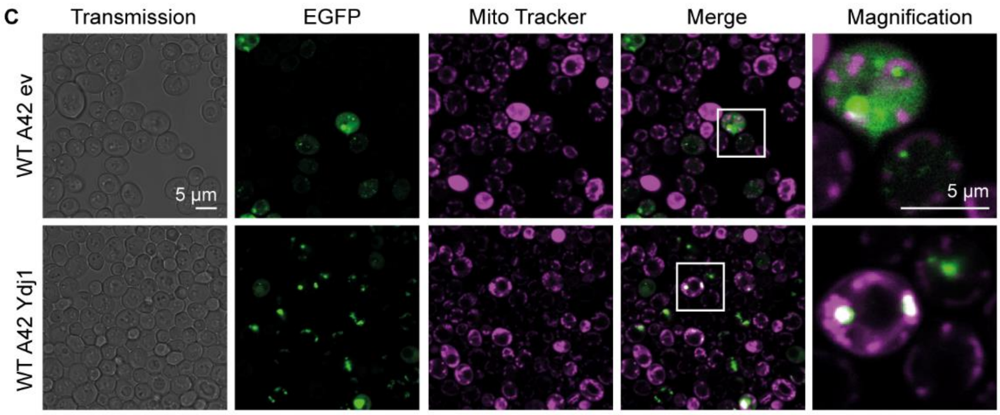

Expanded view 1

17. It would be helpful to see the corresponding results for Abeta40 for S1L.

We agree with the reviewer that this could be interesting, however, due to the extensive number of data already and the fact that Abeta40 shows similar, but less pronounced toxicity, we did not include Abeta40 effects after Ydj1 perturbations into revised manuscript.

18. Ydj1p levels are not clear in overexpression strains. Could the authors somehow quantify this to compare to wild type levels?

We thank the reviewer to pointing this out, we now included immunoblots showing Ydj1 levels in strains used in this study (Fig EV1H).

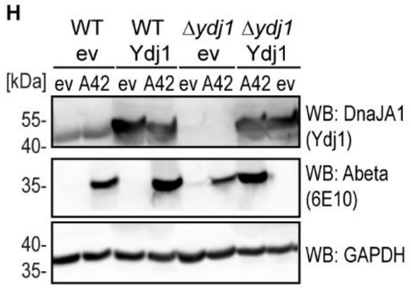

Expanded view 1

19. It is important for the authors to distinguish the direct vs indirect nature of the some of the effects they attribute to Ydj1p. For example, when referring to Ydj1p mediating translocation to the mitochondria, the authors could instead use mitochondria-proximal localization. Also, please add the costained control to 3E.

We agree and as suggested, we adapted the revised manuscript text referring more carefully to “mitochondria-proximity” wherever possible.

We do apologize for the wrong labeling, the co-stained controls are now found and correctly labeled in Fig EV2J.

20. Figures 3-5 are referred to incorrectly multiple times in the text. Please revise.

We thank the reviewer for pointing this out, we revised the referring and corrected it.

21. Panel 4G is a beautiful experiment-very clear.

Thank you for this encouraging comment.

22. The staining for 6E10 in panel 5A lacks other cellular markers - this panel could be greatly strengthened by the addition of other neuronal markers to show intracellular or extracellular staining of Abeta.

Unfortunately, in scope of this revision we did not manage to fully assess Abeta localization in this model. However, we successfully implemented gSTED microscopy allowing us to report for the first-time high resolution of intracellular Abeta in flies. (Fig 6B, 7D, EV5B-C of the revised

manuscript). We do observe mitochondrial contact sites (ATP5A staining) to Abeta signal (Fig EV5C of the revised manuscript), but we are currently limited in quantification of distinct Abeta oligomer distribution between Droj2 (+/-) and Droj2 (+/+) lines, because of the present predominant ER localization of the Abeta signal (Fig. EV5A-B of the revised manuscript) that currently does not allow to discriminate between Mitochondria-ER contact sites versus Abeta to mitochondria translocation events. Therefore, further optimization (e.g. by developing a multi-color gSTED protocol including ER co-staining) of this method is planned in future studies.

<u>We added the following paragraph in the text:</u>

Page 10, lines 7-26:

"In order to assess the effects of Droj2 downregulation on cellular Abeta distribution in male flies, we utilized gSTED microscopy, a super-resolution technique allowing a lateral resolution of approximately 40 nm (Pooryasin et al., 2021). Double staining for the ER marker KDEL and 6E10 antibodies in fly whole mount brains revealed partial co-localization of Abeta with ER in Kenyon cells (intrinsic neurons of the Mushroom Body), based on Pearson's and Mander's coefficients, in line with the expression of Abeta42 within the ER/secretory pathway in this model. No significant differences in co-localization were observed between wildtype and Droj2 knockdown (Fig EV5A-B). Comprehensive gSTED assessment of Abeta cellular localization relative to the ATP synthase subunit ATP5A (an inner mitochondrial membrane marker), revealed mitochondrial-Abeta contact sites (Fig EV5C). This observation goes in line with our cell fractionation data obtained utilizing Kar2-Abeta42 yeast model (Fig EV2E). Even though detected Abeta in close proximity to mitochondria, a quantitative analysis to determine whether this could be affected by Droj2 is technically challenging. This would require to discrimination ER-mitochondria contact sites, considering the high degree of Abeta/ER co-localization observed in both Abeta expressing wild type and Droj2 knockdown flies. Previous reports showed Abeta induced alterations in mitochondrial morphology in Mushroom Body neurons (Wang and Davis, 2021), we therefore decided to continue analyzing mitochondrial morphology changes upon Droj2 downregulation by gSTED microscopy. In Abeta expressing flies, Droj2 knock down (Droj2+/- compared to Droj2+/+) decreased solidity (Fig 7D, E) (Napoli et al., 2021) and circularity (Fig 7D, F) (Kalkhoran et al., 2017), resulting in a mitochondrial morphology more similar to wild type flies (Fig 7D-F, EV5D-H). These, at first sight rather moderate effects, are in line with the 40% reduction of Droj2 mRNA in the Droj2+/- strain."

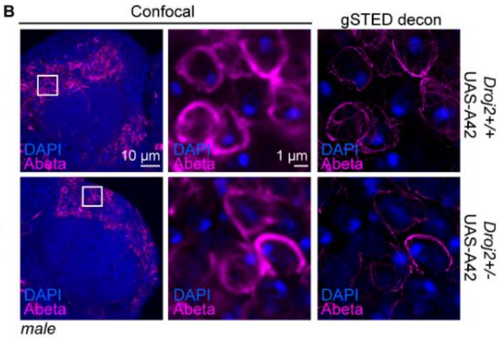

Figure 6

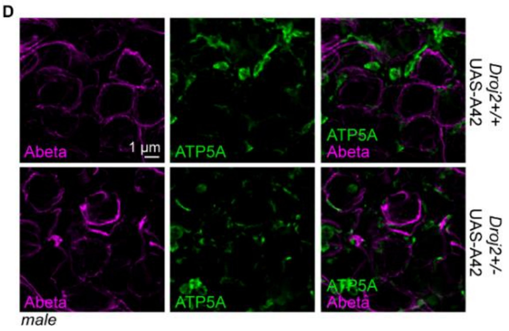

Figure 7

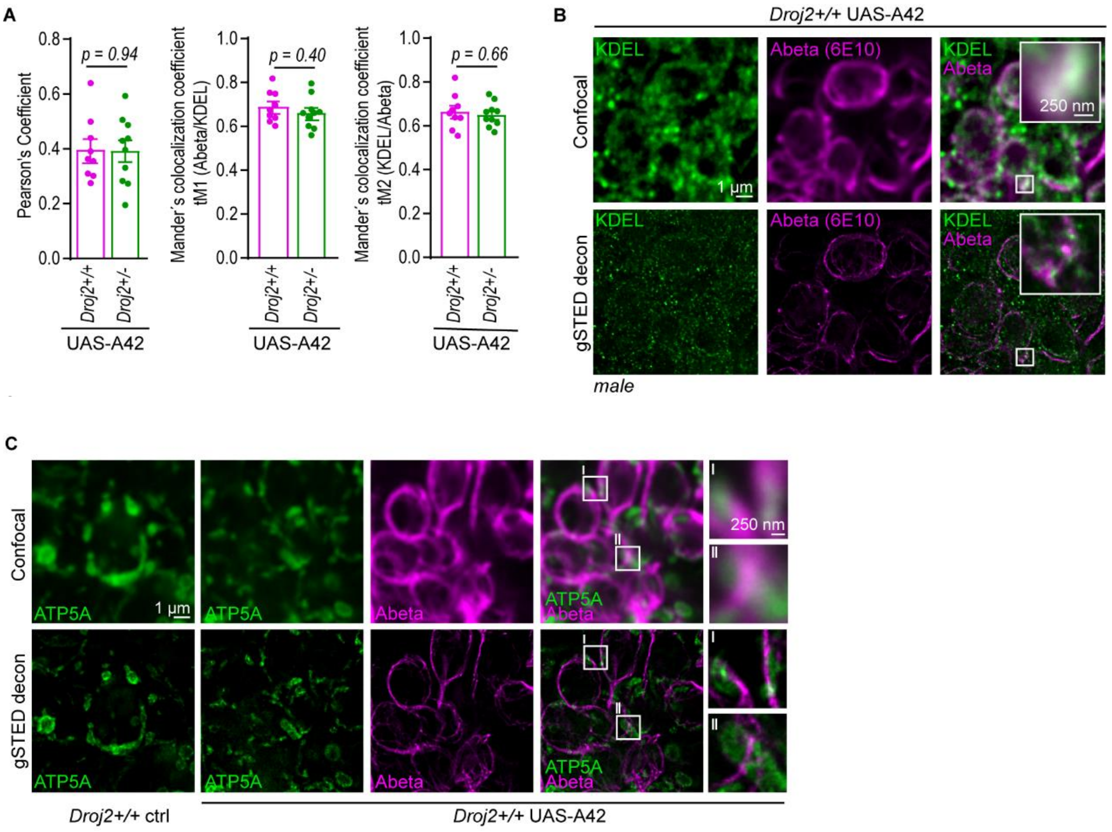

Expanded view 5

23. To support one of the authors' points in the discussion, clusterin and crystallin have also been shown to stabilize oligomeric Abeta (PMIDS: 22179788, PMID: 17412999, PMID: 23106396), and inhibit fibrillization (PMID: 20632140) though shown in these studies for Abeta40, not 42.

We agree with this statement, and therefore have discussed this interesting relation on the page line:

Page 13, lines 6-13:

"…*Recent progress in understanding amyloid kinetics enabled resolving the effects of well-known chaperones on specific microscopic stages of Abeta42 aggregation. Inhibitory effects of the chaperone clusterin on fibril elongation have been reported (Scheidt et al., 2019). Simultaneously, another group provided evidence of clusterin involvement in early stages of AD using the 5×FAD mouse model. They postulated that clusterin binding to Abeta42 oligomers might protect soluble toxic intermediates from enzymatic degradation and thus stabilize them (Oh et al., 2019). In addition, another study performed by Stege and colleagues demonstrated that the small HSP alphaB-crystallin is able to prevent Abeta fibrillization, stabilizing non-fibrillar neurotoxic species (Stege et al., 1999)*. …"

Referee #3 (Comments on Novelty/Model System for Author):

Please note that all ***page and line*** *cross references refer to the final version of the manuscript (not to the track change file)*

While the data are overall conclusive and experiments well controlled, the pathophysiological relevance of these findings for Alzheimer's disease are unclear given the artificial nature of the experimental systems used in this study. It would be important to validate the findings in a more appropriate model of AD

We fully agree with the Referee that our employed models have limitations. Please find below a more detailed response to this important point and describing our efforts to gain first results from mouse models and human brain samples.

Referee #3 (Remarks for Author):

Ring et al. describe the identification of the HSP40 family member Ydj1 as a modulator of aggregation and cellular toxicity in yeast and fly models of Aβ42 related toxicity. Ydj1 was identified in a screen with yeast deletion mutants that rescue toxicity of an EGFP-Aβ42. Interaction of EGFP-Aβ42 and Ydj1 was confirmed by pulldown assays. Biochemical and cell biological experiments suggest that Ydj1 interferes with fibril formation of EGFP-Aβ42, but stabilizes oligomeric assemblies and promotes association of this fusion protein with mitochondria. Knockdown of the Drosophila Ydj1 homolog decreased mortality and reduced Aβ42 induced impairment of olfactory short term memory.

While the data are overall conclusive and experiments well controlled, the pathophysiological relevance of these findings for Alzheimer's disease are unclear given the artificial nature of the experimental systems used in this study. It would be important to validate the findings in a more appropriate model of AD.

We fully agree that the employed models have their limitations and may not model the full spectrum of the human disease. However, Alzheimer’s disease is still an untreatable disease and genetically tractable models may help us dissecting conserved molecular mechanisms of Abeta toxicity and thus provide valuable insights for developing new concepts for AD intervention. In principle, AD mouse models would be a logic next step on a way to untangle DnaJA1 functions towards Abeta toxicity. However, the given time frame and current resources does not allow us to establish a DnaJA1 knock down mouse line in an AD model background, which can only be pursued in follow up studies at this point. The lack of specific DnaJA1 inhibitors (see also responses to *referee #1, Minor Points* for details) further complicates alternative strategies that would be possible in shorter time frames.

Importantly, in the scope of this revision, we were able to utilize brain homogenates of 15 months old female mice from the 3xTg mouse model (Oddo et al., 2003) to investigate DnaJA1 binding properties towards Abeta. We demonstrate that DnaJA1 co-precipitates with different Abeta

oligomeric species as well as full length APP from enriched cytosolic fractions (see also below our response to the specific points and Fig 5A of the revised manuscript).

Furthermore, in order to translate our findings to human relevance, we investigated DnaJA1 protein levels in post-mortem human hippocampi of AD patients. We could demonstrate that DnaJA1 is downregulated in AD post-mortem brain tissue (Fig 5B-C of the revised manuscript) and further discuss in the revised manuscript potential relations of this finding to our results from model systems (*page 13, lines 14-26 of the revised manuscript*). Moreover, DnaJA1 has been recognized as a major ‘pathogenic gene’ (Tao et al., 2020), arguing for a DnaJA1’s crucial role in AD, while the exact dynamics of DnaJA1 in aging and AD in humans is yet to be resolved.

<u>We added the following paragraph in the text:</u>

Page 13, lines 14-26:

“…Interestingly, DnaJA1 is downregulated in post-mortem brain samples of patients who suffered from AD (Abisambra et al., 2012; Sorrentino et al., 2017) and has been postulated as one of the major AD- and MCI-associated genes using bioinformatic meta-analysis (Tao et al., 2020). In a similar fashion to our results, this particular chaperone was upregulated as part of an early heat-shock response in spinocerebellar ataxia-7 (SCA7) -patient derived fibroblasts (Scholefield et al., 2014) and in young SCA7 transgenic mouse model, while it was downregulated in older SCA7 mice (Chou et al., 2010). Age-dependent regulation of DnaJA1 levels in cortical tissue has been already reported with highest expression in teenagers and young adults (16 – 23 years) (Breen et al., 2018). At the same time, oligomeric Abeta shows temporal profiles throughout aging, some being already present at early age long before first symptoms occur (Lesne, 2014). Accordingly, AD progression most likely starts at least two or three decades before actual diagnosis. These data together with our results suggest that DnaJA1 plays a crucial role in AD, but it remains yet to be determined at which stage of this malady DnaJA1 may exert its pathological actions.”

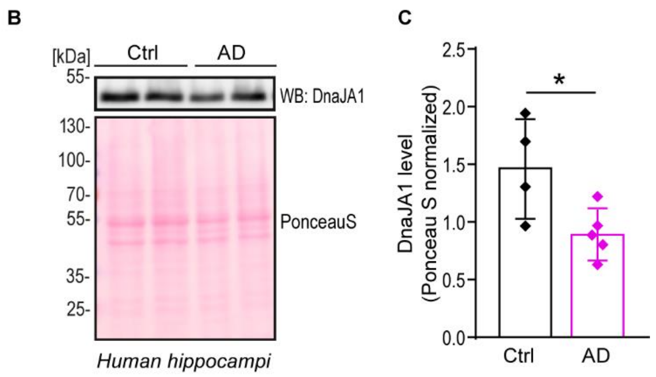

Figure 5

Specific comments:

1. Authors used an EGFP-Aβ42 fusion construct to induce toxicity in yeast and show that EGFP-Aβ42 forms aggregates in the cytosol. Although intraneuronal Aβ is commonly detected in AD brains, these deposits appear to be mainly vesicular. It would be important to demonstrate Aβ peptides in the cytosol and the interaction with DnaJ with more appropriate neuronal model with either endogenous or transgenic expression of APP or addition of extracellular Aβ. The Aβ42 transgenic Drosophila model and Kar2-A42 yeast model also have limitations as there is very high expression of the peptide not derived from its precursor APP in the secretory pathway causing aggregation in the lumen of vesicular compartment and cell stress with unclear relation to the situation in vivo.

As suggested, we utilized the 3xTg mouse model (Oddo et al., 2003) and analyzed brain homogenates of 15 months old female mice to investigate DnaJA1 binding properties towards Abeta species produced by APP proteolysis, following a previously published procedure by Walls et al. (Walls et al., 2012). We demonstrate that DnaJA1 co-precipitates with different Abeta oligomeric species as well as full length APP from enriched cytosolic fractions (Fig 5A of the revised manuscript).

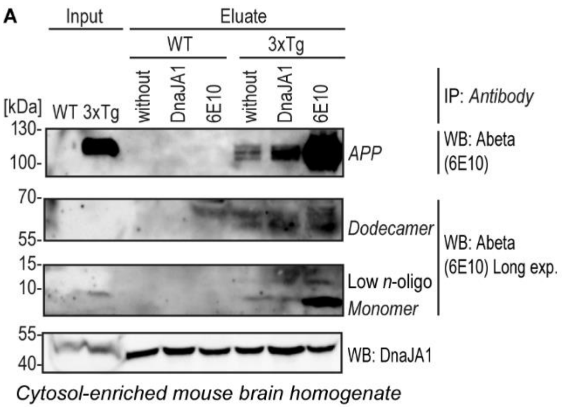

Figure 5

2. Authors identified an Aβ reactive band at 100 kDa and mentioned this could be a specific tetrameric assembly of EGFP-Aβ42 that preferentially associates mitochondria. It should be tested whether such a tetramer also associates with Ydj1/DnaJA1 in above mentioned neuronal models and mouse or human brains.

We would like to point out that we observed DnaJA1 affecting not only tetramer formation (Fig. 4F, Fig 4G-H), but also Abeta oligomerization in general (Fig EV2C-D, Fig 4G-H) (see below also our response to point 4). We apologize if this was misleading in the original manuscript and have adapted our text to better reflect presence and impact on other oligomeric forms.

Utilizing the 3xTg mouse model, we could demonstrate that DnaJA1 interacts with the Abeta peptide sequence, by showing DnaJA1 binding to low-n oligomers and the dodecamer (Fig 5A of

the revised manuscript). However, in this assay we were unable to detect the tetrameric form, which is likely due to technical limitations that arose from our aim to perform this assay from appropriate cell fractions:
Extraction of Abeta oligomers demands highly sophisticated protocols (Casali and Landreth, 2016), which we were not able to fully adapt to cell fractionation and following pull down assays, due to the need of combining 3 individual protocols, demanding very different buffer conditions. Due to such method limitations, we cannot rule out that the tetrameric form of Abeta has been lost during extraction, fractionation and pull-down procedures. Nevertheless, we did quantify tetramer abundance with and without DnaJA1 incubation in our *in vitro* assay (Fig S2E), showing DnaJA1 effects towards formation of this oligomer *in vitro*.

3. The effect of Ydj1/DnaJA1 overexpression and deletion on Aβ induced toxicity and mitochondial activity should be tested in neurons. Primary cultures or iPSC models could be used.

We do agree that appropriate cell culture experiments could add value to our findings, but are afraid that at the moment this is beyond our capabilities and also was not possible to achieve in collaboration at present. Nevertheless, as stated above in more detail at our response to the referees' general comments, we instead investigated DnaJA1 levels in post mortem human hippocampus, where we found downregulation of this protein, arguing for DnaJA1 involvement in AD (Fig 5B-C of the revised manuscript).

4. Ydj1/DnaJA1 coimmunoprecipited with EGFP-Aβ42 monomers (Fig.3c). Does it also coprecipitate the tetramer?

The protein extraction protocol used for immunoprecipitation differs from our standard cell lysis protocol used for immunoblotting. As we did not detect the tetrameric species in the input of the immunoprecipitation experiment (Point-by-Point Fig VA), the absence of the tetrameric form limits to draw any conclusion at present about whether DNAJA1 would also coprecipitate the tetrameric form of Abeta 42.

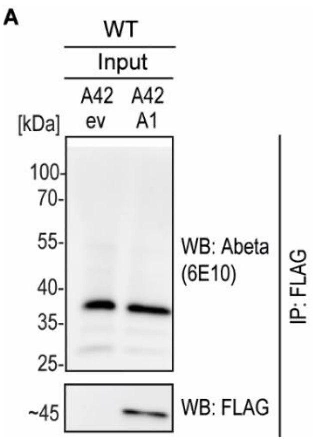

Point-by-Point Fig V

DnaJA1 seems not to promote formation of a tetramer from synthetic Aβ42, but rather of higher oligomeric or fibrillar structures (Fig.4G). How does this relate to the tetramer found with the EGFP-Aβ42 construct? It would be important to characterise these structures (e.g. by TEM or AFM).

We thank the reviewer for pointing this out. Indeed, the most prominent impact of DnaJA1 is observed on higher-n oligomeric structures in this assay. We would like to note that such high-n oligomers cannot be observed on immunoblots with the larger protein size when an EGFP tag is introduced, for which reason we used primarily the tetrameric form for quantification of oligomer abundances in our EGFP-based yeast system. Importantly, using the Kar2-based yeast system as well as in the in vitro oligomerization assays, both the tetrameric and higher-n oligomers are visible and revealed that Ydj1/DnaJA1 stabilized all of these forms.

Encouraged by this comment, we quantified formation of the tetramer by evaluating tetramer/monomer ratio, showing that there is actually a moderate increase in tetramer abundance when DnaJA1 protein is added. Therefore, we can carefully draw the conclusion that tetrameric form may be one of the Abeta species also being targeted by DnaJA1, even though there is a stronger increase in high-n oligomer species over time. We introduced this new quantification to the revised manuscript (Fig 4G, Supplemental Fig S2E).

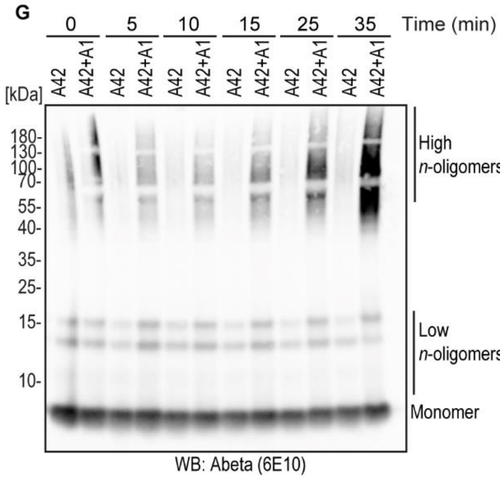

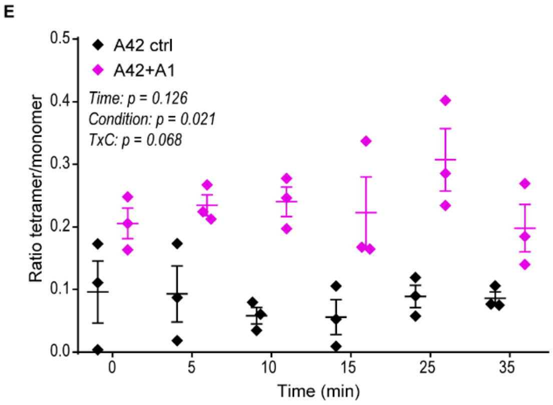

Figure 4 **Supplemental Fig S2**

<u>For clarification, we also introduced a brief discussion of this in the text of the revised manuscript</u> (page 8, lines 14-20):

"*…Of note, both low- and high-n oligomers of up to 180 kDa in size were visible in this assay, while higher fibrils and large aggregates were not detected. Abeta42 oligomerization was observed shortly after the beginning of the incubation period independently of the presence of DnaJA1 (Fig 4G). Nevertheless, the presence of DnaJA1 strongly accelerated oligomer (low- and high-n) formation as shown by an increased total oligomer/monomer ratio (Fig 4H) as well as tetramer/monomer ratio (Fig S2E) throughout the time course experiment.*"

Figure removed upon request from Authors

5. Authors mention that DnaJA1 interferes with ThT readings. Congo red could be used as an alternative compound to assess formation of β sheet aggregates.

In principle, this would be a good idea to try, however, unfortunately, Congo Red cannot be used in our set up, due to device limitations that do not allow appropriate detection of Congo Red.

Additional points.

- References to figures 3 and 4 are erroneously asigned in the text.

We are grateful for pointing this out, we corrected the referring in the text.

- Uathurs mention in the discussion „Nevertheless, Abeta can be produced by proteolysis of APP in intracellular space as well (Wilson et al., 1999), where it can escape the secretory pathway (Bückig et

al., 2002) and be transferred from intracellular membranes (e.g. the ER) into the cytosol (Skovronsky et al., 1998; Wild-Bode et al., 1997)."

It is unclear what is meant with „proteolysis of APP in intracellular space"? The escape of Aβ from the secretory pathay is very controversial. As mentioned above it would be crucial to validate the present findings in a more appropriate neuronal model.

We agree that many of the aspects of the source and generation of intracellular Abeta are yet unclear. To better account for some controversy in this field, we adapted our text:

Page 2, lines 27-33:

Abeta has also been reported to be produced by proteolysis of APP from membranes inside the cell such as the ER or the trans-Golgi network (Greenfield et al., 1999; Hartmann et al., 1997; Wilson et al., 1999). As a consequence, although still speculative, Abeta may be able to escape the secretory pathway (Bückig et al., 2002; Umeda et al., 2011) ending up in the cytosol. Intracellular Abeta has been found all over the cytoplasm, including endosomes, multivesicular bodies, lysosomes, mitochondria, ER, Golgi and the cytosol, where it interferes with the function of diverse organelles (Goldstein et al., 2003; Gouras et al., 2000; Hansson Petersen et al., 2008; Skovronsky et al., 1998; Takahashi et al., 2017).

References

Abisambra, J.F., Jinwal, U.K., Suntharalingam, A., Arulselvam, K., Brady, S., Cockman, M., Jin, Y., Zhang, B., and Dickey, C.A. (2012). DnaJA1 Antagonizes Constitutive Hsp70-Mediated Stabilization of Tau. Journal of Molecular Biology *421*, 653–661.

Akber, U., Jo, H., Jeon, S., Yang, S.-J., Bong, S., Lim, S., Kim, Y.K., Park, Z.-Y., and Park, C.-S. (2021). Cereblon Regulates the Proteotoxicity of Tau by Tuning the Chaperone Activity of DNAJA1. J. Neurosci. *41*, 5138–5156.

Barbitoff, Y.A., Matveenko, A.G., Bondarev, S.A., Maksiutenko, E.M., Kulikova, A.V., and Zhouravleva, G.A. (2020). Quantitative assessment of chaperone binding to amyloid aggregates identifies specificity of Hsp40 interaction with yeast prion fibrils. FEMS Yeast Res *20*, foaa025.

Bitan, G., Kirkitadze, M.D., Lomakin, A., Vollers, S.S., Benedek, G.B., and Teplow, D.B. (2003). Amyloid β-protein (Aβ) assembly: Aβ40 and Aβ42 oligomerize through distinct pathways. PNAS *100*, 330–335.

Breen, M.S., Ozcan, S., Ramsey, J.M., Wang, Z., Ma'ayan, A., Rustogi, N., Gottschalk, M.G., Webster, M.J., Weickert, C.S., Buxbaum, J.D., et al. (2018). Temporal proteomic profiling of postnatal human cortical development. Transl Psychiatry *8*, 1–14.

Bueckig, A., Tikkanen, R., Herzog, V., and Schmitz, A. (2002). Cytosolic and nuclear aggregation of the amyloid ?-peptide following its expression in the endoplasmic reticulum. Histochemistry and Cell Biology *118*, 353–360.

Casali, B.T., and Landreth, G.E. (2016). Aβ Extraction from Murine Brain Homogenates. Bio Protoc *6*, e1787.

Chen, X., and Petranovic, D. (2015). Amyloid-β peptide-induced cytotoxicity and mitochondrial dysfunction in yeast. FEMS Yeast Res *15*, fov061.

Chen, X., Muñoz-Arellano, A.J., and Petranovic, D. (2021). UBB+1 reduces amyloid-β cytotoxicity by activation of autophagy in yeast. Aging (Albany NY) *13*, 23953–23980.

Chou, A.-H., Chen, C.-Y., Chen, S.-Y., Chen, W.-J., Chen, Y.-L., Weng, Y.-S., and Wang, H.-L. (2010). Polyglutamine-expanded ataxin-7 causes cerebellar dysfunction by inducing transcriptional dysregulation. Neurochemistry International *56*, 329–339.

Dahlgren, K.N., Manelli, A.M., Stine, W.B., Baker, L.K., Krafft, G.A., and LaDu, M.J. (2002). Oligomeric and fibrillar species of amyloid-beta peptides differentially affect neuronal viability. J Biol Chem *277*, 32046–32053.

Finch, C.E., and Austad, S.N. (2015). Commentary: is Alzheimer's disease uniquely human? Neurobiol Aging *36*, 553–555.

Goldstein, L.E., Muffat, J.A., Cherny, R.A., Moir, R.D., Ericsson, M.H., Huang, X., Mavros, C., Coccia, J.A., Faget, K.Y., Fitch, K.A., et al. (2003). Cytosolic β-amyloid deposition and supranuclear cataracts in lenses from people with Alzheimer's disease. The Lancet *361*, 1258–1265.

Gouras, G.K., Tsai, J., Naslund, J., Vincent, B., Edgar, M., Checler, F., Greenfield, J.P., Haroutunian, V., Buxbaum, J.D., Xu, H., et al. (2000). Intraneuronal Aβ42 Accumulation in Human Brain. The American Journal of Pathology *156*, 15–20.

Greenfield, J.P., Tsai, J., Gouras, G.K., Hai, B., Thinakaran, G., Checler, F., Sisodia, S.S., Greengard, P., and Xu, H. (1999). Endoplasmic reticulum and trans-Golgi network generate distinct populations of Alzheimer beta -amyloid peptides. Proceedings of the National Academy of Sciences *96*, 742–747.

Hansson Petersen, C.A., Alikhani, N., Behbahani, H., Wiehager, B., Pavlov, P.F., Alafuzoff, I., Leinonen, V., Ito, A., Winblad, B., Glaser, E., et al. (2008). The amyloid -peptide is imported into mitochondria via the TOM import machinery and localized to mitochondrial cristae. Proceedings of the National Academy of Sciences *105*, 13145–13150.

Hartmann, T., Bieger, S.C., Brühl, B., Tienari, P.J., Ida, N., Allsop, D., Roberts, G.W., Masters, C.L., Dotti, C.G., Unsicker, K., et al. (1997). Distinct sites of intracellular production for Alzheimer's disease Aβ40/42 amyloid peptides. Nat Med *3*, 1016–1020.

Iijima, K., Chiang, H.-C., Hearn, S.A., Hakker, I., Gatt, A., Shenton, C., Granger, L., Leung, A., Iijima-Ando, K., and Zhong, Y. (2008). Aβ42 Mutants with Different Aggregation Profiles Induce Distinct Pathologies in Drosophila. PLoS ONE *3*, e1703.

Kalkhoran, S.B., Munro, P., Qiao, F., Ong, S.-B., Hall, A.R., Cabrera-Fuentes, H., Chakraborty, B., Boisvert, W.A., Yellon, D.M., and Hausenloy, D.J. (2017). Unique morphological characteristics of mitochondrial subtypes in the heart: the effect of ischemia and ischemic preconditioning. Discoveries (Craiova) *5*, e71.

Lesne, S.E. (2014). Toxic oligomer species of amyloid-β in Alzheimer's disease, a timing issue. Swiss Med Wkly *144*, w14021.

Månsson, C., Arosio, P., Hussein, R., Kampinga, H.H., Hashem, R.M., Boelens, W.C., Dobson, C.M., Knowles, T.P.J., Linse, S., and Emanuelsson, C. (2014). Interaction of the Molecular Chaperone DNAJB6 with Growing Amyloid-beta 42 (Aβ42) Aggregates Leads to Sub-stoichiometric Inhibition of Amyloid Formation *. Journal of Biological Chemistry *289*, 31066–31076.

Napoli, E., Panoutsopoulos, A.A., Kysar, P., Satriya, N., Sterling, K., Shibata, B., Imai, D., Ruskin, D.N., Zarbalis, K.S., and Giulivi, C. (2021). Wdfy3 regulates glycophagy, mitophagy, and synaptic plasticity. J Cereb Blood Flow Metab 0271678X211027384.

Newington, J.T., Pitts, A., Chien, A., Arseneault, R., Schubert, D., and Cumming, R.C. (2011). Amyloid beta resistance in nerve cell lines is mediated by the Warburg effect. PLoS One *6*, e19191.

Newington, J.T., Rappon, T., Albers, S., Wong, D.Y., Rylett, R.J., and Cumming, R.C. (2012). Overexpression of pyruvate dehydrogenase kinase 1 and lactate dehydrogenase A in nerve cells

confers resistance to amyloid β and other toxins by decreasing mitochondrial respiration and reactive oxygen species production. J Biol Chem *287*, 37245–37258.

Oddo, S., Caccamo, A., Shepherd, J.D., Murphy, M.P., Golde, T.E., Kayed, R., Metherate, R., Mattson, M.P., Akbari, Y., and LaFerla, F.M. (2003). Triple-Transgenic Model of Alzheimer’s Disease with Plaques and Tangles: Intracellular Aβ and Synaptic Dysfunction. Neuron *39*, 409–421.

Oh, S.-B., Kim, M.S., Park, S., Son, H., Kim, S.-Y., Kim, M.-S., Jo, D.-G., Tak, E., and Lee, J.-Y. (2019). Clusterin contributes to early stage of Alzheimer’s disease pathogenesis: Clusterin in Alzheimer’s disease pathogenesis. Brain Pathology *29*, 217–231.

Österlund, N., Lundqvist, M., Ilag, L.L., Gräslund, A., and Emanuelsson, C. (2020). Amyloid-β oligomers are captured by the DNAJB6 chaperone: Direct detection of interactions that can prevent primary nucleation. J Biol Chem *295*, 8135–8144.

Paranjpe, M.D., Belonwu, S., Wang, J.K., Oskotsky, T., Gupta, A., Taubes, A., Zalocusky, K.A., Paranjpe, I., Glicksberg, B.S., Huang, Y., et al. (2021). Sex-Specific Cross Tissue Meta-Analysis Identifies Immune Dysregulation in Women With Alzheimer’s Disease. Front Aging Neurosci *13*, 735611.

Park, S.-K., Arslan, F., Kanneganti, V., Barmada, S.J., Purushothaman, P., Verma, S.C., and Liebman, S.W. (2018). Overexpression of a conserved HSP40 chaperone reduces toxicity of several neurodegenerative disease proteins. Prion *12*, 16–22.

Pooryasin, A., Maglione, M., Schubert, M., Matkovic-Rachid, T., Hasheminasab, S.-M., Pech, U., Fiala, A., Mielke, T., and Sigrist, S.J. (2021). Unc13A and Unc13B contribute to the decoding of distinct sensory information in Drosophila. Nat Commun *12*, 1932.

Rodríguez-González, C., Lin, S., Arkan, S., and Hansen, C. (2020). Co-chaperones DNAJA1 and DNAJB6 are critical for regulation of polyglutamine aggregation. Sci Rep *10*, 8130.

Scheidt, T., Łapińska, U., Kumita, J.R., Whiten, D.R., Klenerman, D., Wilson, M.R., Cohen, S.I.A., Linse, S., Vendruscolo, M., Dobson, C.M., et al. (2019). Secondary nucleation and elongation occur at different sites on Alzheimer’s amyloid-β aggregates. Science Advances *5*, eaau3112.

Scholefield, J., Watson, L., Smith, D., Greenberg, J., and Wood, M.J. (2014). Allele-specific silencing of mutant Ataxin-7 in SCA7 patient-derived fibroblasts. Eur J Hum Genet *22*, 1369–1375.

Skovronsky, D.M., Doms, R.W., and Lee, V.M.-Y. (1998). Detection of a Novel Intraneuronal Pool of Insoluble Amyloid β Protein that Accumulates with Time in Culture. Journal of Cell Biology *141*, 1031–1039.

Sorrentino, V., Romani, M., Mouchiroud, L., Beck, J.S., Zhang, H., D’Amico, D., Moullan, N., Potenza, F., Schmid, A.W., Rietsch, S., et al. (2017). Enhancing mitochondrial proteostasis reduces amyloid-β proteotoxicity. Nature.

Stege, G.J.J., Renkawek, K., Overkamp, P.S.G., Verschuure, P., van Rijk, A.F., Reijnen-Aalbers, A., Boelens, W.C., Bosman, G.J.C.G.M., and de Jong, W.W. (1999). The Molecular Chaperone αB-crystallin Enhances Amyloid β Neurotoxicity. Biochemical and Biophysical Research Communications *262*, 152–156.

Stein, K.C., Bengoechea, R., Harms, M.B., Weihl, C.C., and True, H.L. (2014). Myopathy-causing Mutations in an HSP40 Chaperone Disrupt Processing of Specific Client Conformers *. Journal of Biological Chemistry *289*, 21120–21130.

Takahashi, R.H., Nagao, T., and Gouras, G.K. (2017). Plaque formation and the intraneuronal accumulation of β-amyloid in Alzheimer’s disease: Intraneuronal accumulation of β-amyloid. Pathol Int *67*, 185–193.

Tao, Y., Han, Y., Yu, L., Wang, Q., Leng, S.X., and Zhang, H. (2020a). The Predicted Key Molecules, Functions, and Pathways That Bridge Mild Cognitive Impairment (MCI) and Alzheimer’s Disease (AD). Frontiers in Neurology *11*, 233.

Tao, Y., Han, Y., Yu, L., Wang, Q., Leng, S.X., and Zhang, H. (2020b). The Predicted Key Molecules, Functions, and Pathways That Bridge Mild Cognitive Impairment (MCI) and Alzheimer's Disease (AD). Front. Neurol. *11*, 233.

Tittelmeier, J., Nachman, E., and Nussbaum-Krammer, C. (2020). Molecular Chaperones: A Double-Edged Sword in Neurodegenerative Diseases. Frontiers in Aging Neuroscience *12*, 321.

Treusch, S., Hamamichi, S., Goodman, J.L., Matlack, K.E.S., Chung, C.Y., Baru, V., Shulman, J.M., Parrado, A., Bevis, B.J., Valastyan, J.S., et al. (2011). Functional Links Between A Toxicity, Endocytic Trafficking, and Alzheimer's Disease Risk Factors in Yeast. Science *334*, 1241–1245.

Umeda, T., Tomiyama, T., Sakama, N., Tanaka, S., Lambert, M.P., Klein, W.L., and Mori, H. (2011). Intraneuronal amyloid β oligomers cause cell death via endoplasmic reticulum stress, endosomal/lysosomal leakage, and mitochondrial dysfunction in vivo. J. Neurosci. Res. *89*, 1031–1042.

Walls, K.C., Coskun, P., Gallegos-Perez, J.L., Zadourian, N., Freude, K., Rasool, S., Blurton-Jones, M., Green, K.N., and LaFerla, F.M. (2012). Swedish Alzheimer mutation induces mitochondrial dysfunction mediated by HSP60 mislocalization of amyloid precursor protein (APP) and beta-amyloid. J Biol Chem *287*, 30317–30327.

Wang, X., and Davis, R.L. (2021). Early Mitochondrial Fragmentation and Dysfunction in a Drosophila Model for Alzheimer's Disease. Mol Neurobiol *58*, 143–155.

Whitmore, A., Freeny, D., Sojourner, S.J., Miles, J.S., Graham, W.M., and Flores-Rozas, H. (2020). Evaluation of the Role of Human DNAJAs in the Response to Cytotoxic Chemotherapeutic Agents in a Yeast Model System. BioMed Research International *2020*, e9097638.

Wilson, C.A., Doms, R.W., and Lee, V.M.-Y. (1999). Intracellular APP Processing and Aβ Production in Alzheimer Disease: Journal of Neuropathology and Experimental Neurology *58*, 787–794.

Wisén, S., Bertelsen, E.B., Thompson, A.D., Patury, S., Ung, P., Chang, L., Evans, C.G., Walter, G.M., Wipf, P., Carlson, H.A., et al. (2010). Binding of a small molecule at a protein-protein interface regulates the chaperone activity of hsp70-hsp40. ACS Chem Biol *5*, 611–622.

Zacharias, D.A., Violin, J.D., Newton, A.C., and Tsien, R.Y. (2002). Partitioning of Lipid-Modified Monomeric GFPs into Membrane Microdomains of Live Cells. Science *296*, 913–916.

1st Feb 2022

Dear Prof. Madeo,

Thank you for the submission of your revised manuscript to EMBO Molecular Medicine. We have now received the enclosed report from the two referees who were asked to re-assess it. As you will see, the referees are now supportive, and I am pleased to inform you that we will be able to accept your manuscript pending the following amendments:

On a more editorial level, please do the following:

I look forward to reading a new revised version of your manuscript as soon as possible.

Kind regards,
Jingyi

Jingyi Hou
Editor
EMBO Molecular Medicine

*** Instructions to submit your revised manuscript ***

*** PLEASE NOTE *** As part of the EMBO Publications transparent editorial process initiative (see our Editorial at https://www.embopress.org/doi/pdf/10.1002/emmm.201000094), EMBO Molecular Medicine will publish online a Review Process File to accompany accepted manuscripts.

In the event of acceptance, this file will be published in conjunction with your paper and will include the anonymous referee reports, your point-by-point response and all pertinent correspondence relating to the manuscript. If you do NOT want this file to be published, please inform the editorial office at contact@embomolmed.org.

To submit your manuscript, please follow this link:

https://embomolmed.msubmit.net/cgi-bin/main.plex

When submitting your revised manuscript, please include:

1) a .docx formatted version of the manuscript text (including Figure legends and tables)

2) Separate figure files*

3) supplemental information as Expanded View and/or Appendix. Please carefully check the authors guidelines for formatting Expanded view and Appendix figures and tables at https://www.embopress.org/page/journal/17574684/authorguide#expandedview

4) a letter INCLUDING the reviewer's reports and your detailed responses to their comments (as Word file).

5) The paper explained: EMBO Molecular Medicine articles are accompanied by a summary of the articles to emphasize the major findings in the paper and their medical implications for the non-specialist reader. Please provide a draft summary of your article highlighting
- the medical issue you are addressing,
- the results obtained and
- their clinical impact.
This may be edited to ensure that readers understand the significance and context of the research.
Please refer to any of our published articles for an example.

6) For more information: There is space at the end of each article to list relevant web links for further consultation by our readers. Could you identify some relevant ones and provide such information as well? Some examples are patient associations, relevant databases, OMIM/proteins/genes links, author's websites, etc...

7) Author contributions: the contribution of every author must be detailed in a separate section.

8) EMBO Molecular Medicine now requires a complete author checklist (https://www.embopress.org/page/journal/17574684/authorguide) to be submitted with all revised manuscripts. Please use the checklist as guideline for the sort of information we need WITHIN the manuscript. The checklist should only be filled with page numbers were the information can be found. This is particularly important for animal reporting, antibody dilutions (missing) and exact values and n that should be indicted instead of a range.

9) Every published paper now includes a 'Synopsis' to further enhance discoverability. Synopses are displayed on the journal webpage and are freely accessible to all readers. They include a short stand first (maximum of 300 characters, including space) as well as 2-5 one sentence bullet points that summarise the paper. Please write the bullet points to summarise the key NEW findings. They should be designed to be complementary to the abstract - i.e. not repeat the same text. We encourage inclusion of key acronyms and quantitative information (maximum of 30 words / bullet point). Please use the passive voice. Please attach these in a separate file or send them by email, we will incorporate them accordingly.

You are also welcome to suggest a striking image or visual abstract to illustrate your article. If you do please provide a jpeg file 550 px-wide x 400-px high.

10) A Conflict of Interest statement should be provided in the main text

11) Please note that we now mandate that all corresponding authors list an ORCID digital identifier. This takes <90 seconds to complete. We encourage all authors to supply an ORCID identifier, which will be linked to their name for unambiguous name identification.

Currently, our records indicate that the ORCID for your account is 0000-0002-5070-1329.

Please click the link below to modify this ORCID:
Link Not Available

12) The system will prompt you to fill in your funding and payment information. This will allow Wiley to send you a quote for the article processing charge (APC) in case of acceptance. This quote takes into account any reduction or fee waivers that you may be eligible for. Authors do not need to pay any fees before their manuscript is accepted and transferred to our publisher.

*Additional important information regarding Figures

Each figure should be given in a separate file and should have the following resolution:
Graphs 800-1,200 DPI
Photos 400-800 DPI
Colour (only CMYK) 300-400 DPI"

Figures are not edited by the production team. All lettering should be the same size and style; figure panels should be indicated by capital letters (A, B, C etc). Gridlines are not allowed except for log plots. Figures should be numbered in the order of their appearance in the text with Arabic numerals. Each Figure must have a separate legend and a caption is needed for each panel.

*Additional important information regarding figures and illustrations can be found at https://bit.ly/EMBOPressFigurePreparationGuideline. See also figure legend preparation guidelines: https://www.embopress.org/page/journal/17574684/authorguide#figureformat

The system will prompt you to fill in your funding and payment information. This will allow Wiley to send you a quote for the article processing charge (APC) in case of acceptance. This quote takes into account any reduction or fee waivers that you may be eligible for. Authors do not need to pay any fees before their manuscript is accepted and transferred to our publisher.

***** Reviewer's comments *****

Referee #1 (Comments on Novelty/Model System for Author):

Adequate considering the exploratory and early stage PoC nature of the study.

Referee #1 (Remarks for Author):

The authors addressed my concerns to satisfaction and they did it as much as can be considering the tools available for research on this topic.

Referee #2 (Remarks for Author):

The authors have responded to most of our prior critiques. We support publication.

The authors have made all requested editorial changes.

15th Feb 2022

We are pleased to inform you that your manuscript is accepted for publication and is now being sent to our publisher to be included in the next available issue of EMBO Molecular Medicine.

We would like to remind you that as part of the EMBO Publications transparent editorial process initiative, EMBO Molecular Medicine will publish a Review Process File online to accompany accepted manuscripts. If you do NOT want the file to be published or would like to exclude figures, please immediately inform the editorial office via e-mail.

Please read below for additional IMPORTANT information regarding your article, its publication and the production process.

Congratulations on your interesting work,
Jingyi

Jingyi Hou
Editor
EMBO Molecular Medicine

Follow us on Twitter @EmboMolMed
Sign up for eTOCs at embopress.org/alertsfeeds

*** *** *** IMPORTANT INFORMATION *** *** ***

SPEED OF PUBLICATION
The journal aims for rapid publication of papers, using the advance online publication "Early View" to expedite the process: A properly copy-edited and formatted version will be published as "Early View" after the proofs have been corrected. Please help the Editors and publisher avoid delays by providing e-mail address(es), telephone and fax numbers at which author(s) can be contacted.

Should you be planning a Press Release on your article, please get in contact with embomolmed@wiley.com as early as possible, in order to coordinate publication and release dates.

LICENSE AND PAYMENT:

All articles published in EMBO Molecular Medicine are fully open access: immediately and freely available to read, download and share.

EMBO Molecular Medicine charges an article processing charge (APC) to cover the publication costs. You, as the corresponding author for this manuscript, should have already received a quote with the article processing fee separately. Please let us know in case this quote has not been received.

Once your article is at Wiley for editorial production you will receive an email from Wiley's Author Services system, which will ask you to log in and will present you with the publication license form for completion. Within the same system the publication fee can be paid by credit card, an invoice, pro forma invoice or purchase order can be requested.

Payment of the publication charge and the signed Open Access Agreement form must be received before the article can be published online.

PROOFS

You will receive the proofs by e-mail approximately 2 weeks after all relevant files have been sent o our Production Office. Please return them within 48 hours and if there should be any problems, please contact the production office at embopressproduction@wiley.com.

Please inform us if there is likely to be any difficulty in reaching you at the above address at that time. Failure to meet our deadlines may result in a delay of publication.

All further communications concerning your paper proofs should quote reference number EMM-2021-13952-V3 and be directed to the production office at embopressproduction@wiley.com.

Thank you,

Jingyi Hou
Editor
EMBO Molecular Medicine

EMBO PRESS

YOU MUST COMPLETE ALL CELLS WITH A PINK BACKGROUND

PLEASE NOTE THAT THIS CHECKLIST WILL BE PUBLISHED ALONGSIDE YOUR PAPER

Corresponding Author Name: Frank Madeo and Tobias Eisenberg

Journal Submitted to: EMBO Molecular Medicine
Manuscript Number: EMM-2021-13952-V2

USEFUL LINKS FOR COMPLETING THIS FORM

http://www.antibodypedia.com
http://1degreebio.org
http://www.equator-network.org/reporting-guidelines/improving-bioscience-research-repor
http://grants.nih.gov/grants/olaw/olaw.htm
http://www.mrc.ac.uk/Ourresearch/Ethicsresearchguidance/Useofanimals/index.htm
http://ClinicalTrials.gov
http://www.consort-statement.org
http://www.consort-statement.org/checklists/view/32-consort/66-title
http://www.equator-network.org/reporting-guidelines/reporting-recommendations-for-tum
http://datadryad.org
http://figshare.com
http://www.ncbi.nlm.nih.gov/gap
http://www.ebi.ac.uk/ega
http://biomodels.net/
http://biomodels.net/miriam/
http://jjj.biochem.sun.ac.za
https://osp.od.nih.gov/biosafety-biosecurity-and-emerging-biotechnology/
http://www.selectagents.gov/

Reporting Checklist For Life Sciences Articles (Rev. June 2017)

This checklist is used to ensure good reporting standards and to improve the reproducibility of published results. These guidelines are consistent with the Principles and Guidelines for Reporting Preclinical Research issued by the NIH in 2014. Please follow the journal's authorship guidelines in preparing your manuscript.

A- Figures
1. Data
The data shown in figures should satisfy the following conditions:

- the data were obtained and processed according to the field's best practice and are presented to reflect the results of the experiments in an accurate and unbiased manner.
- figure panels include only data points, measurements or observations that can be compared to each other in a scientifically meaningful way.
- graphs include clearly labeled error bars for independent experiments and sample sizes. Unless justified, error bars should not be shown for technical replicates.
- if n< 5, the individual data points from each experiment should be plotted and any statistical test employed should be justified
- Source Data should be included to report the data underlying graphs. Please follow the guidelines set out in the author ship guidelines on Data Presentation.

2. Captions

Each figure caption should contain the following information, for each panel where they are relevant:

- a specification of the experimental system investigated (eg cell line, species name).
- the assay(s) and method(s) used to carry out the reported observations and measurements
- an explicit mention of the biological and chemical entity(ies) that are being measured.
- an explicit mention of the biological and chemical entity(ies) that are altered/varied/perturbed in a controlled manner.
- the exact sample size (n) for each experimental group/condition, given as a number, not a range;
- a description of the sample collection allowing the reader to understand whether the samples represent technical or biological replicates (including how many animals, litters, cultures, etc.).
- a statement of how many times the experiment shown was independently replicated in the laboratory.
- definitions of statistical methods and measures:
  - common tests, such as t-test (please specify whether paired vs. unpaired), simple $\chi 2$ tests, Wilcoxon and Mann-Whitney tests, can be unambiguously identified by name only, but more complex techniques should be described in the methods section;
  - are tests one-sided or two-sided?
  - are there adjustments for multiple comparisons?
  - exact statistical test results, e.g., P values = x but not P values < x;
  - definition of 'center values' as median or average;
  - definition of error bars as s.d. or s.e.m.

Any descriptions too long for the figure legend should be included in the methods section and/or with the source data.

In the pink boxes below, please ensure that the answers to the following questions are reported in the manuscript itself. Every question should be answered. If the question is not relevant to your research, please write NA (non applicable). We encourage you to include a specific subsection in the methods section for statistics, reagents, animal models and human subjects.

B- Statistics and general methods

Please fill out these boxes (Do not worry if you cannot see all your text once you press return)

1.a. How was the sample size chosen to ensure adequate power to detect a pre-specified effect size?	Sample sizes of yeast and fly experiments were chosen based on standard power analysis (statistical power: ≥ 0.8 and α value: < 0.05) or on previous published studies (Pooryasin et al., 2021 and Chen et al. 2020). Human tissue sample analysis was explorative and conducted without pre-specified effect size.
1.b. For animal studies, include a statement about sample size estimate even if no statistical methods were used.	Analysis of mouse brain homogenates was of explorative nature and therefore no sample size estimates were calculated.
2. Describe inclusion/exclusion criteria if samples or animals were excluded from the analysis. Were the criteria pre-established?	No samples or data points were excluded from any analysis, except for technical reasons.
3. Were any steps taken to minimize the effects of subjective bias when allocating animals/samples to treatment (e.g. randomization procedure)? If yes, please describe.	NA
For animal studies, include a statement about randomization even if no randomization was used.	NA
4.a. Were any steps taken to minimize the effects of subjective bias during group allocation or/and when assessing results (e.g. blinding of the investigator)? If yes please describe.	In case of potential subjective bias (e.g. microscopic analysis of fly brain regions, memory experiments in flies, etc.) experiments as well as analysis were done blinded.
4.b. For animal studies, include a statement about blinding even if no blinding was done	NA
5. For every figure, are statistical tests justified as appropriate?	Yes. Details are included in Statistical Section of the Methods and the employed tests for each Figure subpanel stated in the respective legends.
Do the data meet the assumptions of the tests (e.g., normal distribution)? Describe any methods used to assess it.	Yes. Alle data have been statistically analysed. Statistical testing was performed using OriginPro 2016 statistic software, GraphPad Prism™ 9 software or IBM SPSS statistics software - Version 25. Normal distribution of data was confirmed using Shapiro-Wilk's test. Homogeneity of variance was tested using Levene's test or by visual inspection of QQ-plots of residuals. Data violating these assumptions were transformed to meet the assumptions of linear models. In case of a 2-way repeated measures ANOVA with the time factor set as the repeated variable data were Greenhouse-Geisser-corrected in case of sphericity violation (using GraphPad Prism Software, Version 9).
Is there an estimate of variation within each group of data?	Yes. Whereever appropriate, standard deviation (s.d.) is shown for descritpive statistics of data and all data points are plotted.

Is the variance similar between the groups that are being statistically compared?	Variance was tested using Levene's test and variance was not significantly different between groups that have been compared using ANOVA or t-test analysis. In case of sphericity violation in 2-way repeated measure ANOVA analysis, Greenhouse-Geisser correction was applied.

C- Reagents

6. To show that antibodies were profiled for use in the system under study (assay and species), provide a citation, catalog number and/or clone number, supplementary information or reference to an antibody validation profile. e.g., Antibodypedia (see link list at top right), 1DegreeBio (see link list at top right).	Antibodies were all described in Material&Methods section including used dilutions as well as catalog number or citation if not commercially available.
7. Identify the source of cell lines and report if they were recently authenticated (e.g., by STR profiling) and tested for mycoplasma contamination.	NA

* for all hyperlinks, please see the table at the top right of the document

D- Animal Models

8. Report species, strain, gender, age of animals and genetic modification status where applicable. Please detail housing and husbandry conditions and the source of animals.	Breeding pairs of triple transgenic mice (3xTg), carrying the PS1M146V, APPSwe, and tauP301L transgenes (Oddo et al., 2003) were initially purchased from The Jackson Laboratories (JAX), United States of America. Animals used in this study were bred in the animal facility of the Institute of Molecular Biosciences, Graz, Austria using the breeding pairs from Charles River Laboratories. The control strain used for the AD disease model was B6129SF2/J, also bred in-house with breeding pairs from Charles River Laboratories. Briefly, animals were housed in groups of two to four animals per cage under specific-pathogen-free (SPF) conditions in a 14h/10h light/dark cycle with ad libitum access to standard chow (Ssniff, cat. #V1536) and autoclaved tap water. Autoclaved nest material and one paper house per cage served as cage enrichment.
9. For experiments involving live vertebrates, include a statement of compliance with ethical regulations and identify the committee(s) approving the experiments.	All animal experiments were performed in accordance with national and European ethical regulation (Directive 2010/63/EU) and approved by the responsible institutional or government agencies (Bundesministerium für Wissenschaft, Forschung und Wirtschaft, BMWFW, Austria: BMWFW-66.007/0032-V/3b/2019).
10. We recommend consulting the ARRIVE guidelines (see link list at top right) (PLoS Biol. 8(6), e1000412, 2010) to ensure that other relevant aspects of animal studies are adequately reported. See author guidelines, under 'Reporting Guidelines'. See also: NIH (see link list at top right) and MRC (see link list at top right) recommendations. Please confirm compliance.	We confirm compliance to ARRIVE guidelines for animal breeding, housing and tissue collection.

E- Human Subjects

11. Identify the committee(s) approving the study protocol.	Experiments with human materials were in accordance with the ethical committee at the University of Bayreuth (Germany). Postmortem tissues of hippocampi from AD patients and non-demented controls were obtained from the Netherlands Brain Bank (Amsterdam, The Netherlands). Experiments were carried out in laboratories at the University of Bayreuth.
12. Include a statement confirming that informed consent was obtained from all subjects and that the experiments conformed to the principles set out in the WMA Declaration of Helsinki and the Department of Health and Human Services Belmont Report.	For human materials informed consent was obtained from all subjects and the experiments conformed to the principles set out in the WMA Declaration of Helsinki and the Department of Health and Human Services Belmont Report
13. For publication of patient photos, include a statement confirming that consent to publish was obtained.	NA
14. Report any restrictions on the availability (and/or on the use) of human data or samples.	NA
15. Report the clinical trial registration number (at ClinicalTrials.gov or equivalent), where applicable.	NA
16. For phase II and III randomized controlled trials, please refer to the CONSORT flow diagram (see link list at top right) and submit the CONSORT checklist (see link list at top right) with your submission. See author guidelines, under 'Reporting Guidelines'. Please confirm you have submitted this list.	NA
17. For tumor marker prognostic studies, we recommend that you follow the REMARK reporting guidelines (see link list at top right). See author guidelines, under 'Reporting Guidelines'. Please confirm you have followed these guidelines.	NA

F- Data Accessibility

18: Provide a "Data Availability" section at the end of the Materials & Methods, listing the accession codes for data generated in this study and deposited in a public database (e.g. RNA-Seq data: Gene Expression Omnibus GSE39462, Proteomics data: PRIDE PXD000208 etc.) Please refer to our author guidelines for 'Data Deposition'.<br><br>Data deposition in a public repository is mandatory for:<br>a. Protein, DNA and RNA sequences<br>b. Macromolecular structures<br>c. Crystallographic data for small molecules<br>d. Functional genomics data<br>e. Proteomics and molecular interactions	The mass spectrometry proteomics data have been deposited to the ProteomeXchange Consortium via the PRIDE (Perez-Riverol et al., 2019) partner repository with the dataset identifier PXD012612 (Project accession number).
19. Deposition is strongly recommended for any datasets that are central and integral to the study; please consider the journal's data policy. If no structured public repository exists for a given data type, we encourage the provision of datasets in the manuscript as a Supplementary Document (see author guidelines under 'Expanded View' or in unstructured repositories such as Dryad (see link list at top right) or Figshare (see link list at top right).	The mass spectrometry proteomics data have been deposited to the ProteomeXchange Consortium via the PRIDE (Perez-Riverol et al., 2019) partner repository with the dataset identifier PXD012612 (Project accession number).
20. Access to human clinical and genomic datasets should be provided with as few restrictions as possible while respecting ethical obligations to the patients and relevant medical and legal issues. If practically possible and compatible with the individual consent agreement used in the study, such data should be deposited in one of the major public access-controlled repositories such as dbGAP (see link list at top right) or EGA (see link list at top right).	NA
21. Computational models that are central and integral to a study should be shared without restrictions and provided in a machine-readable form. The relevant accession numbers or links should be provided. When possible, standardized format (SBML, CellML) should be used instead of scripts (e.g. MATLAB). Authors are strongly encouraged to follow the MIRIAM guidelines (see link list at top right) and deposit their model in a public database such as Biomodels (see link list at top right) or JWS Online (see link list at top right). If computer source code is provided with the paper, it should be deposited in a public repository or included in supplementary information.	yes

G- Dual use research of concern

22. Could your study fall under dual use research restrictions? Please check biosecurity documents (see link list at top right) and list of select agents and toxins (APHIS/CDC) (see link list at top right). According to our biosecurity guidelines, provide a statement only if it could.	no
