# Supplementary material for: The HSP40 chaperone Ydj1 drives amyloid beta 42 toxicity
Source: EMBO Mol Med. 2022 Apr 4;14(5):e13952. doi: 10.15252/emmm.202113952 (PMC9081910; doi:10.15252/emmm.202113952)
Supplement: Supplementary file 3 — Source Data for Expanded View and Appendix [file EMMM-14-e13952-s010.zip › EMM-2021-13952_Figure_EV4_Source_Data.zip!/EMM-2021-13952-V3-Figure_EV4F_Source_Data-sd.docx]

**Source Data for Figure EV4F: CT values of qPCR.**

| **Experiment** | **Primer** | **Strain** | **CT mean** | **Figure** |
| --- | --- | --- | --- | --- |
|  |  |  |  |  |
| **1** | *Droj2* | *Droj2 +/+* | 27.514 | **EV4F** |
|  |  | *Droj2 +/-* | 28.179 |  |
|  | *Rpl32* | *Droj2 +/+* | 18.535 |  |
|  |  | *Droj2 +/-* | 18.553 |  |
| **2** | *Droj2* | *Droj2 +/+* | 27.865 |  |
|  |  | *Droj2 +/-* | 29.232 |  |
|  | *Rpl32* | *Droj2 +/+* | 18.923 |  |
|  |  | *Droj2 +/-* | 19.008 |  |
| **3** | *Droj2* | *Droj2 +/+* | 27.003 |  |
|  |  | *Droj2 +/-* | 27.466 |  |
|  | *Rpl32* | *Droj2 +/+* | 23.291 |  |
|  |  | *Droj2 +/-* | 23.210 |  |
